# Supplementary material for: Optimization of mtDNA-targeted platinum TALENs for bi-directionally modifying heteroplasmy levels in patient-derived m.3243A>G-iPSCs
Source: Mol Ther Nucleic Acids. 2025 Mar 19;36(2):102521. doi: 10.1016/j.omtn.2025.102521 (PMC12002989; doi:10.1016/j.omtn.2025.102521)
Supplement: Document S1. Figures S1–S15 and Table S1 [file mmc1.pdf]

OMTN, Volume *36*

Supplemental information

Optimization of mtDNA-targeted platinum TALENs for bi-directionally modifying heteroplasmy levels in patient-derived m.3243A>G-iPSCs

Naoki Yahata, Yu-ichi Goto, and Ryuji Hata

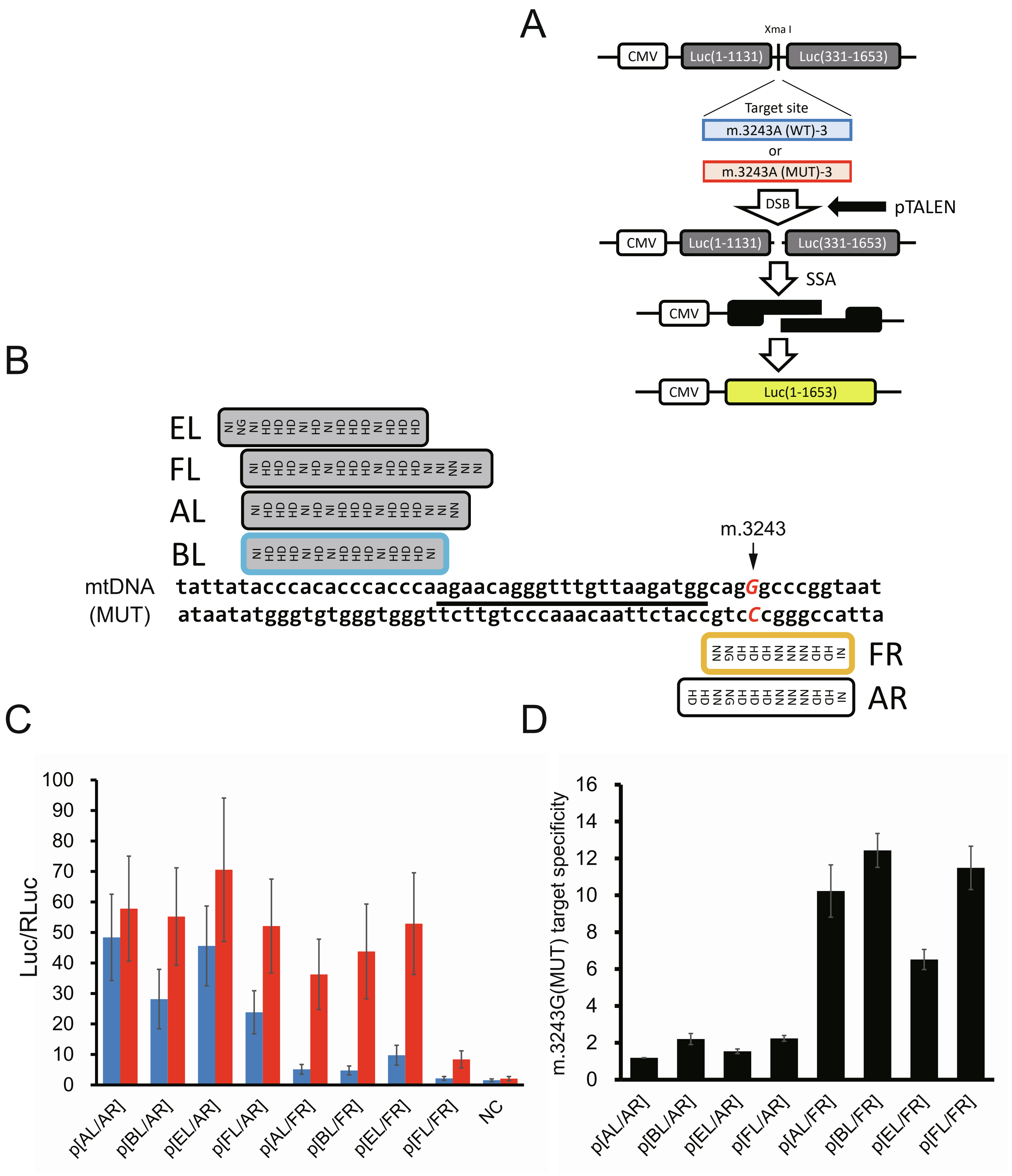

Figure S1

Functional evaluation of engineered m.3243G(MUT)-pTALENs using an SSA assay.

(A) Scheme of the SSA assay. The reporter plasmid encodes a part of the mtDNA sequence including m.3243A or m.3243G, named m.3243A(WT)-3 or m.3243G(MUT)-3, which is sandwiched by two split inactive parts of the luciferase gene with overlapping repeated sequences. Following a double-strand break caused by pTALENs, a functional luciferase gene is generated by an SSA reaction.

(B) Schematic design of m.3243G(MUT)-pTALENs. Gray and White boxes indicate left-pTALENs and right-pTALENs, respectively. Letters beside the boxes indicate the TALEs' names. The black bar indicates the spacer region bracketed by the pTALEN(BL)/pTALEN (FR) pair, abbreviated as p[BL/FR].

(C) Evaluation of the SSA activity (Luc/RLuc) of pTALEN pairs. Blue and red bars reflect the cleaving activities against m.3243A(WT)-3 and m.3243G(MUT)-3, respectively. NC, negative control. Data are expressed as mean ± SEM (n = 3).

(D) m.3243G(MUT) target specificity of each pTALEN pair. Data are expressed as means ± SEM (n = 3).

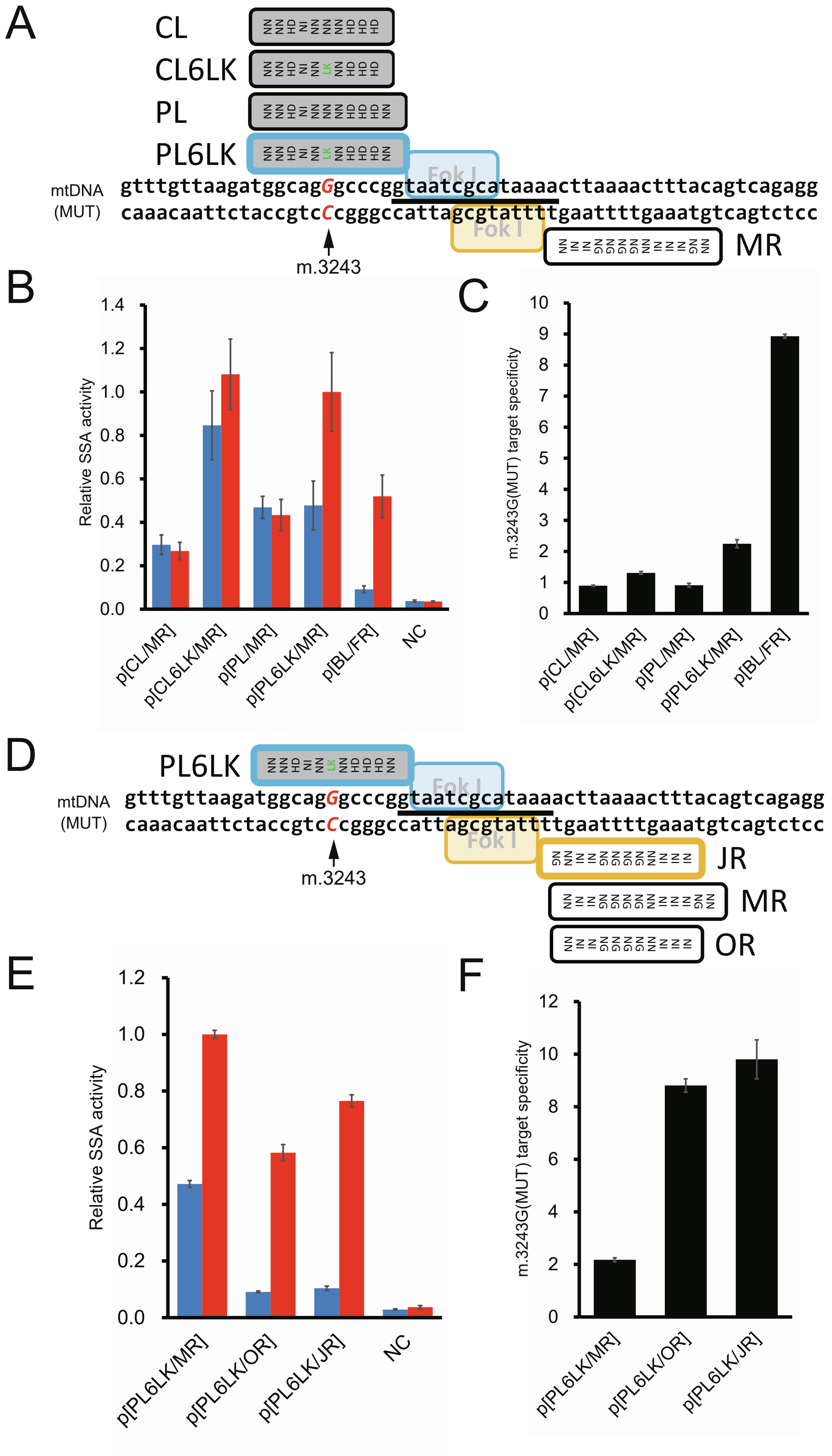

Figure S2

Functional evaluation of engineered m.3243G(MUT)-pTALENs with ncRVDs using an SSA assay.

(A, D) Schematic design of m.3243G(MUT)-pTALENs. Gray and white boxes indicate the left-pTALENs and right-pTALENs, respectively. Letters beside the boxes indicate the TALEs' names. The black bars indicate the spacer regions bracketed by the pTALEN (PL6LK)/pTALEN(MR) or pTALEN(PL6LK)/pTALEN(JR) pair, abbreviated as p[PL6LK/MR] (A) and p[PL6LK/JR] (D), respectively. Green letters indicate ncRVDs.

(B, E) Evaluation of the SSA activity (Luc/RLuc) of pTALEN pairs. Blue and red bars reflect the cleaving activities against human mtDNA sequences, including m.3243A(WT) and m.3243G(MUT), respectively. Relative SSA activity is defined as the ratio of the measured activity to the activity score of p[PL6LK/MR] against mutant mtDNA sequence. Data are expressed as the mean ± SEM (n = 3). NC, negative control.

(C, F) Specificity of each pTALEN pair toward the m.3243G(MUT). Data are expressed as the means ± SEM (n = 3).

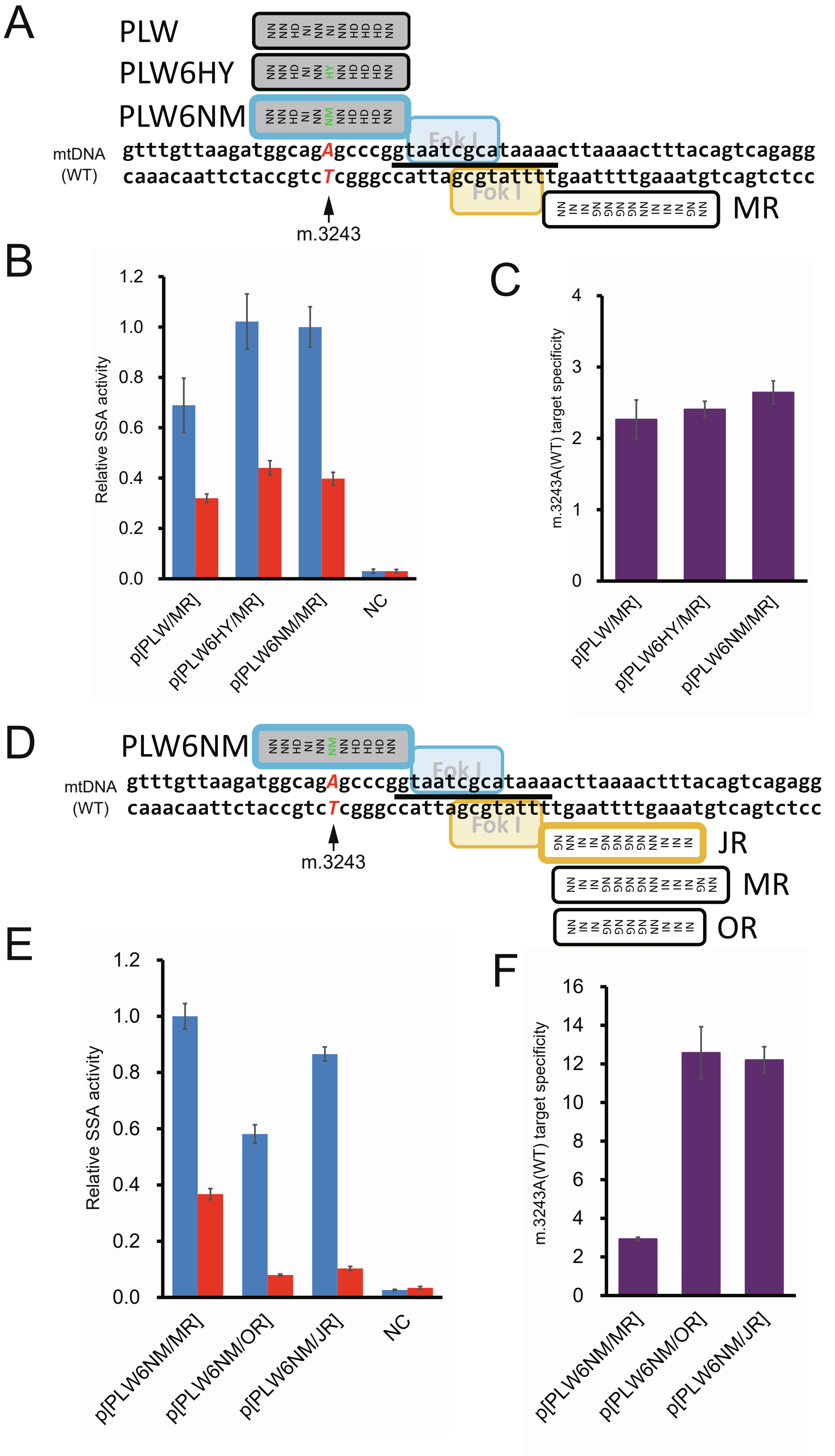

Figure S3

Functional evaluation of engineered m.3243A(WT)-pTALEN with ncRVDs using an SSA assay.

(A, D) Schematic design of m.3243A(WT)-pTALENs. Gray and white boxes indicate left-pTALENs and right-pTALENs, respectively. Letters beside the boxes indicate the TALEs' names. The black bars indicate the spacer regions bracketed by the pTALEN (PLW6NM)/pTALEN(MR) or pTALEN(PLW6NM)/pTALEN(JR) pair, abbreviated as p[PLW6NM/MR] (A) and p[PLW6NM/JR] (D), respectively. Green letters indicate ncRVDs.

(B, E) Evaluation of the SSA activity (Luc/RLuc) of pTALEN pairs. Blue and red bars reflect cleaving activity against human mtDNA sequences, including m.3243A(WT) and m.3243G(MUT), respectively. Relative SSA activity is defined as the ratio of the measured activity to the activity score of p[PLW6NM/MR] against wild-type mtDNA sequence. Data are expressed as the mean ± SEM (n = 3). NC, negative control.

(C, F) Specificity of each pTALEN pair toward the m.3243A(WT). Data are expressed as the means ± SEM (n = 3).

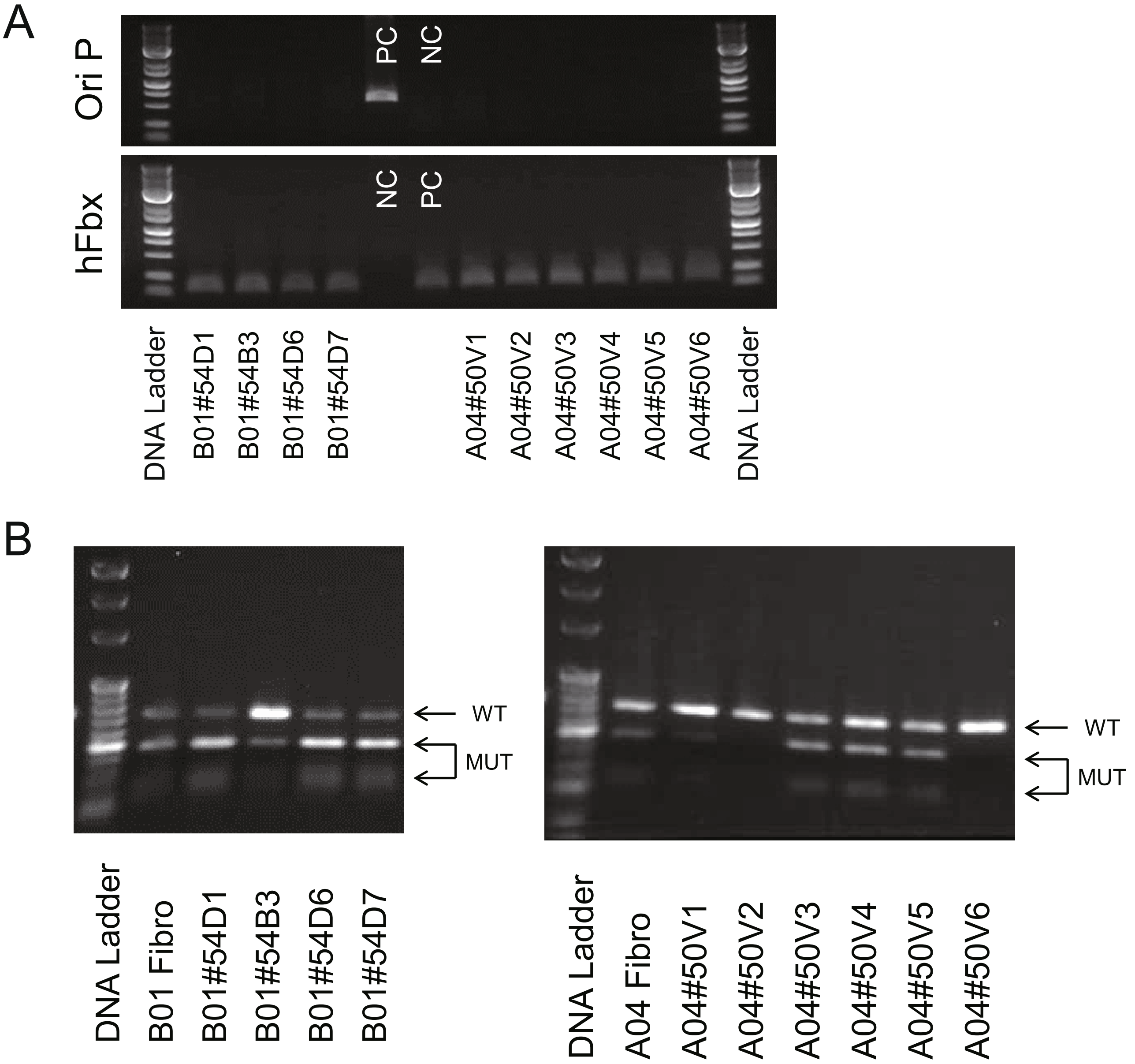

Figure S4

Validation of lack of transgene integration and m.3243A>G heteroplasmy level in isolated iPSC clones.

(A) PCR analysis for confirmation of transgene integration in isolated iPSC clones. In the top lane labeled OriP, PCR primer pair detected the sequence of OriP region in episomal vectors. The bottom lane labeled hFbx showed endogenous allele detection. Lane 6: reprogramming vector mix (pCXLE-hOCT3/4-shp53, pCXLE-hSK and pCXLE-hUL). Lane 7: genomic DNA of human skin fibroblasts. PC, positive control. NC, negative control.

(B) m.3243A>G heteroplasmy levels in mitochondrial disease patients' fibroblasts and isolated iPSC clones were analyzed by PCR-RFLP. The 145 bp and 95 bp + 50 bp fragments indicate the presence of wild-type and mutant mtDNA, respectively.

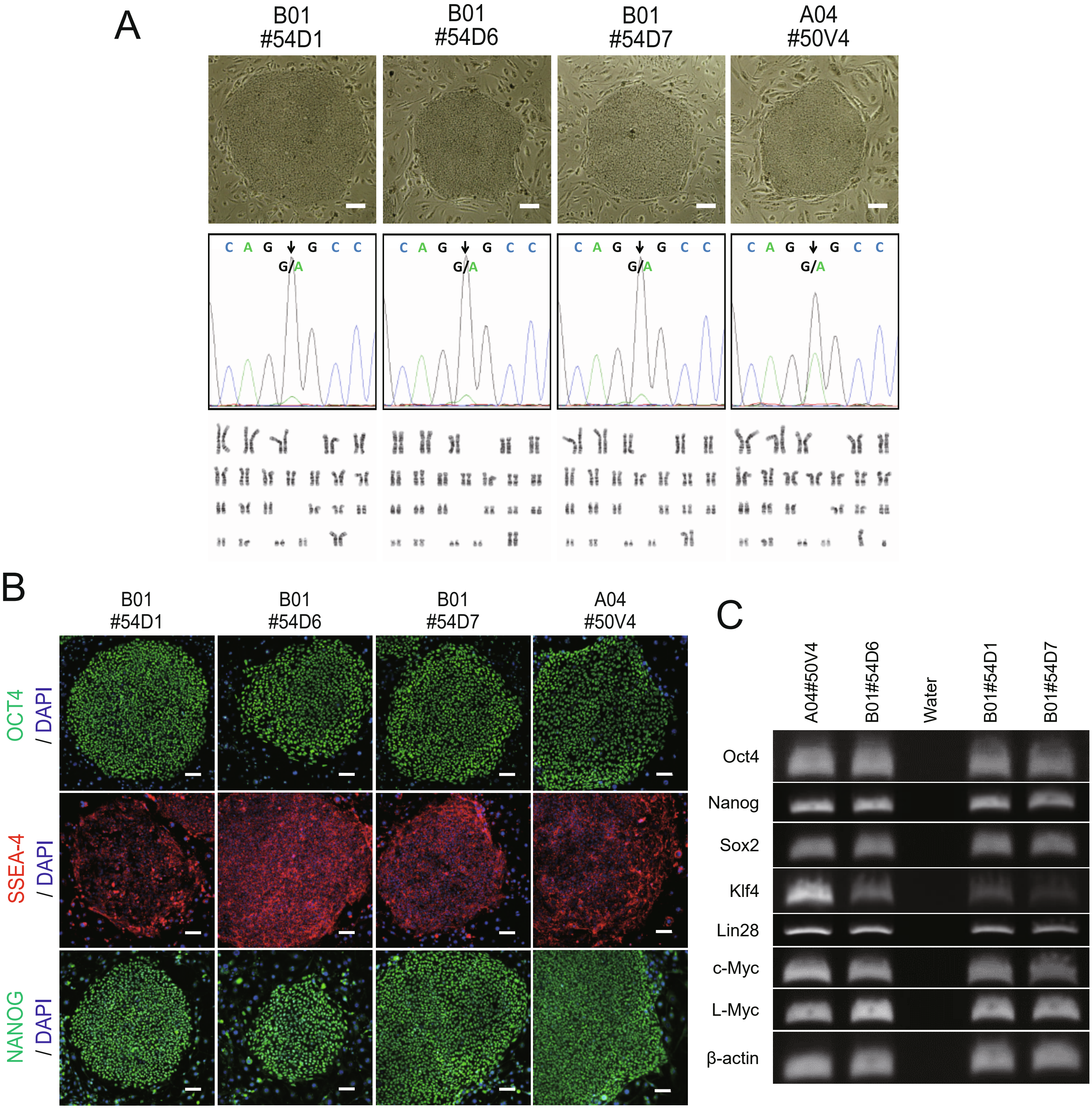

Figure S5

Establishment of human iPSCs from mitochondrial disease patients with m.3243A>G mutation.

(A) Top, phase-contrast images of established m.3243A>G-iPSC clones (B01_#54D1, #54D6, #54D7, and A04_#50V4). Scale bar, 200 μm. Middle, sequences of mtDNA extracted from the four iPSC clones. Arrows indicate the m.3243 position. Bottom, karyotype analysis revealed that these iPSC clones preserved a normal karyotype.

(B) These iPSC clones expressed the human ESC markers Oct4 (green), SSEA-4 (red), and Nanog (green) as indicated by immuno-cytochemical analysis. Scale bar, 200 μm.

(C) RT-PCR analyses of pluripotency markers and reprogramming factors (*Oct4*, *Nanog*, *Sox2*, *Klf4*, *Lin28*, *c-Myc*, and *L-Myc*). *β-actin* serves as a loading control.

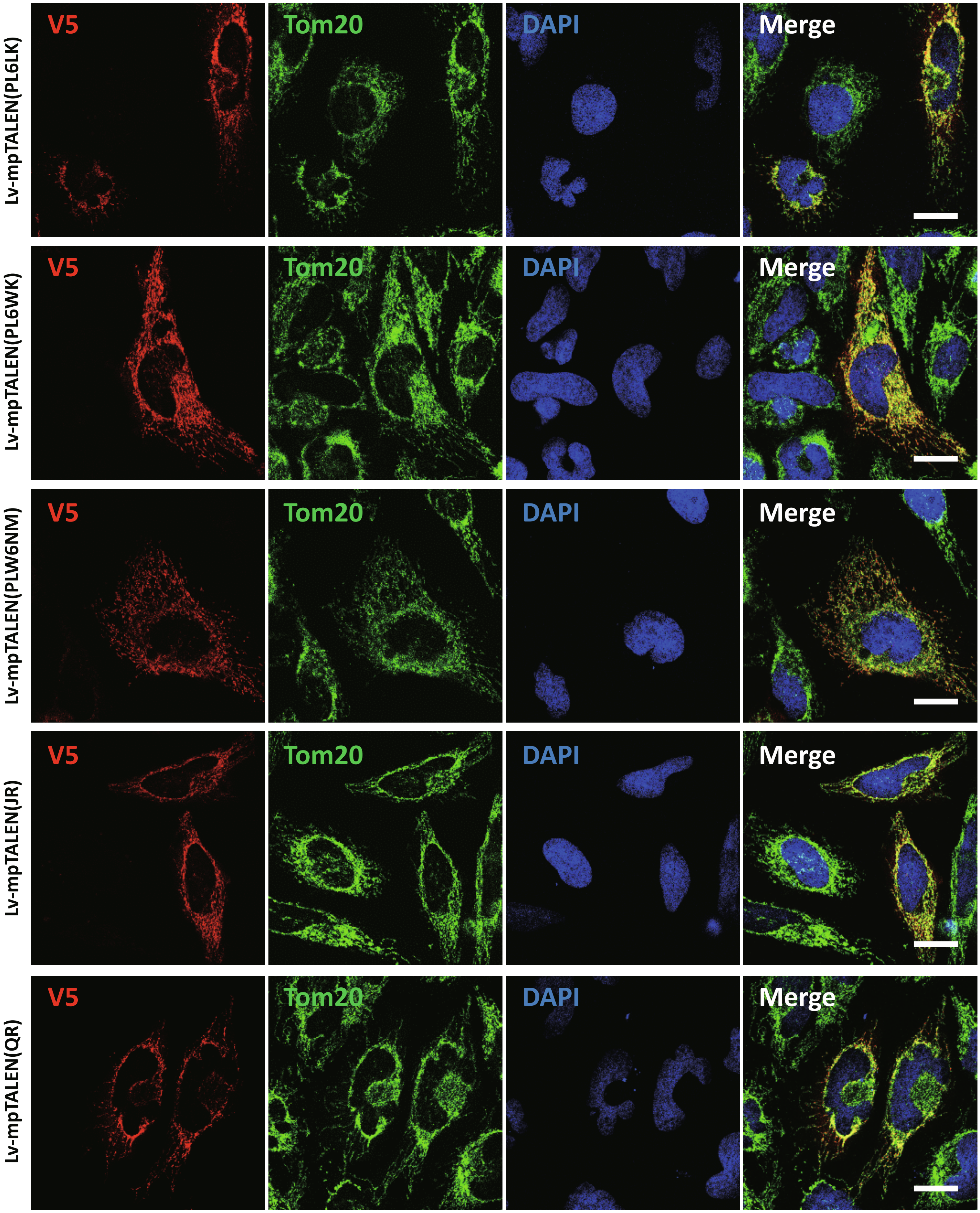

Figure S6

Mitochondrial localization of Lv-mpTALENs analyzed by immunocytochemistry.

The Lv-mpTALEN monomers were transiently expressed in HeLa cells. Two days after transfection, Lv-mp-TALEN(PL6LK), Lv-mpTALEN(PL6WK), Lv-mpTALEN(PLW6NM), Lv-mpTALEN(JR), and Lv-mpTALEN(QR) were stained using an anti-V5 antibody (red). Mitochondria were stained using an anti-TOM20 antibody (green). Nuclei were stained with DAPI (blue). Scale bar, 20 μm.

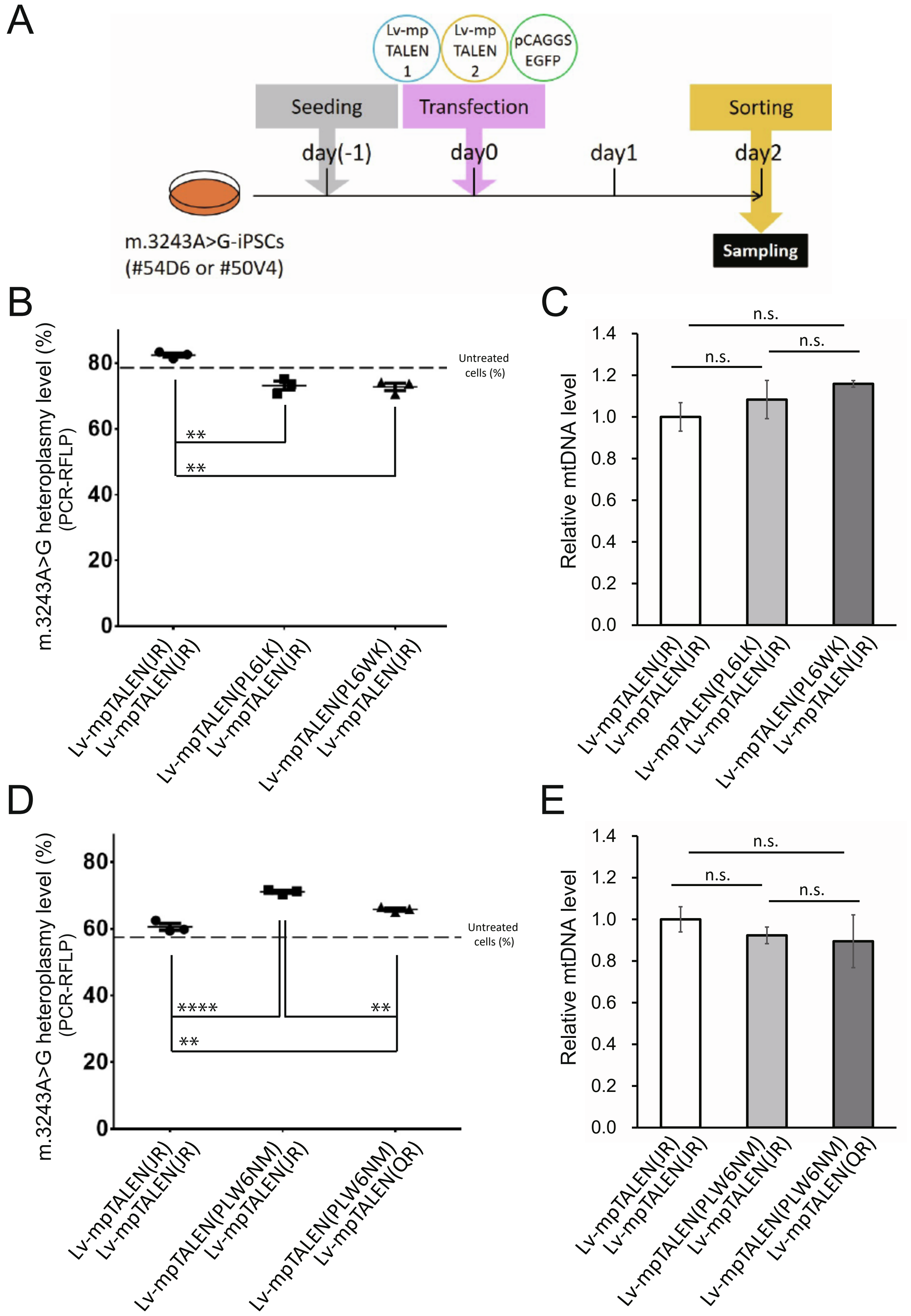

Figure S7

Effects of selected mpTALEN pairs on heteroplasmy levels in m.3243A>G-iPSCs.

(A) Experimental scheme for the application of mpTALEN pairs to m.3243A>G-iPSCs. m.3243A>G-iPSCs (#54D6 or #50V4) were transfected with plasmids coding left- and right-mpTALENs and EGFP. EGFP-positive and living cells were analyzed on day 2 after transfection.
(B, D) m.3243A>G heteroplasmy level in #54D6-iPSCs transfected with m.3243G(MUT)-mpTALEN pairs (B) and in #50V4-iPSCs transfected with m.3243A(WT)-mpTALEN pairs (D), respectively. Data are expressed as the means ± SEM (n = 3). Dotted lines indicate the heteroplasmy level in untreated cells on day 2. **p < 0.01, ****p < 0.0001 (one-way ANOVA, followed by Tukey' s multiple comparison test).
(C, E) Relative mtDNA levels (correlating with mtDNA copy number) in #54D6-iPSCs (C) and in #50V4-iPSCs (E), respectively. Data are presented as the ratio of the measured number to that in "mock-treated" controls (Lv-mpTALEN(JR)/Lv-mpTALEN(JR)) and expressed as the mean ± SEM (n = 3). n.s., not significant (one-way ANOVA, followed by Tukey' s multiple comparison test).

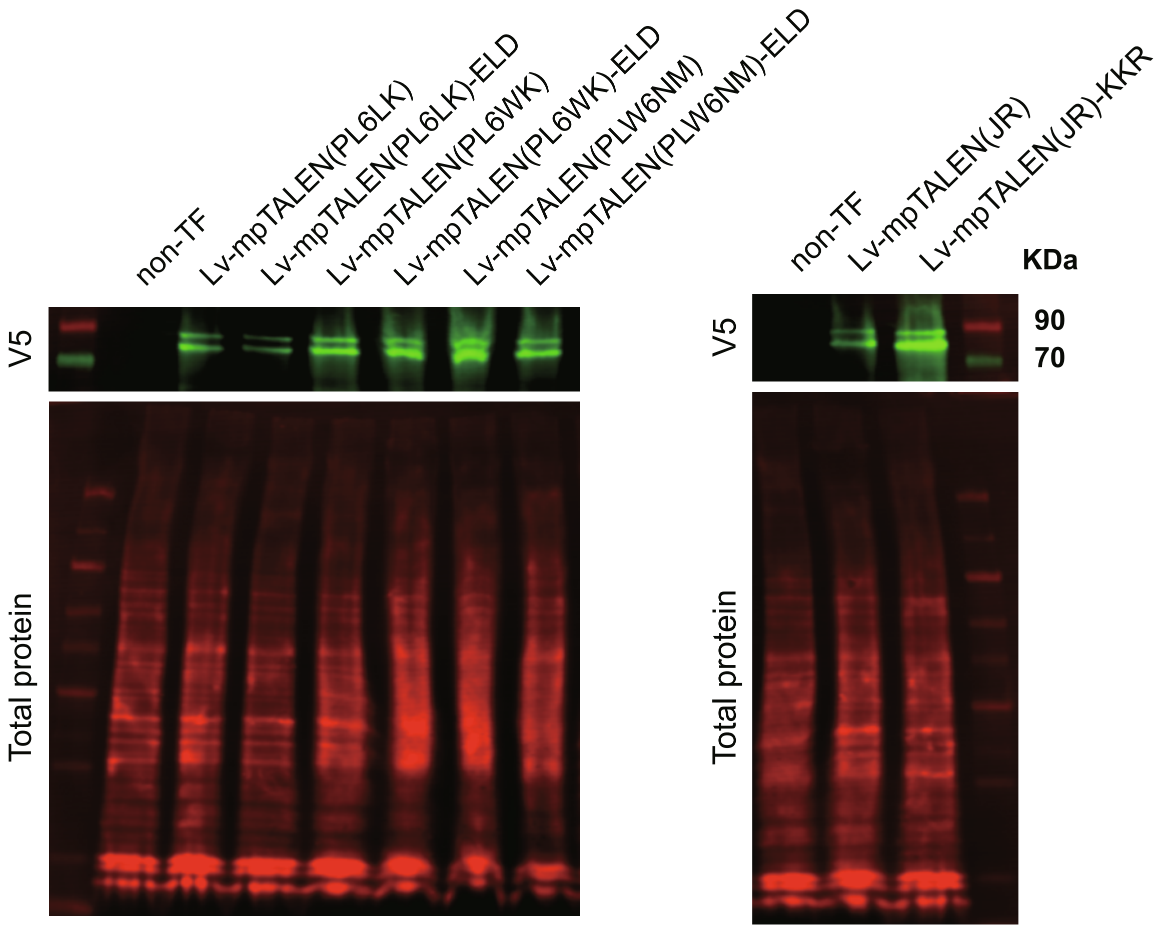

Figure S8

The mpTALEN proteins analyzed by Western blotting.

HEK293T cells were transfected with plasmids coding Lv-mpTALENs. Two days after transfection, the mpTALEN protein in cell extracts was detected by Western blotting using an anti-V5 antibody. Total protein was detected using Revert 700 Total Protein Stain (LI-COR). non-TF, non-transfected cells.

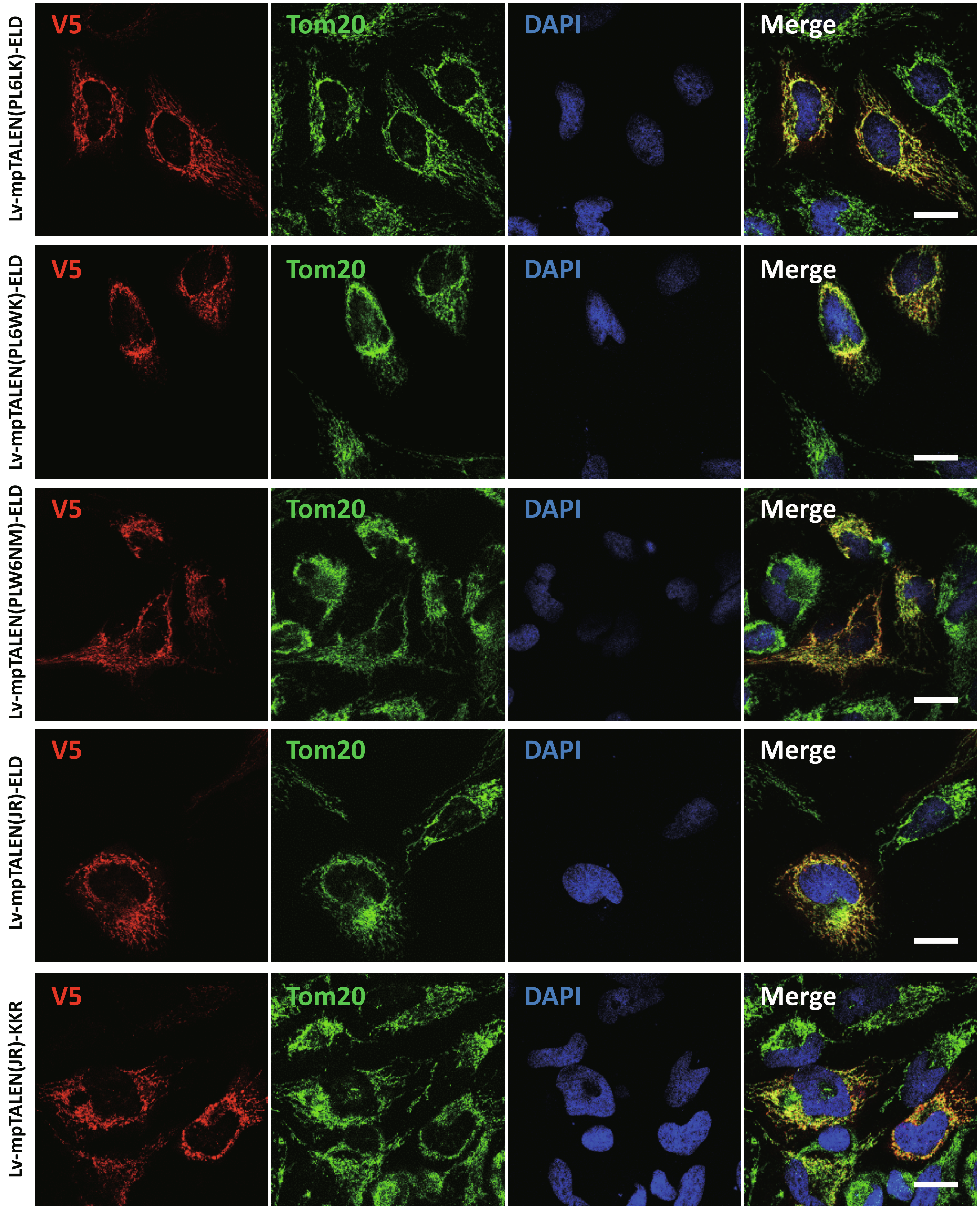

Figure S9

Mitochondrial localization of heterodimeric Lv-mpTALENs analyzed by immunocytochemistry.

The heterodimeric Lv-mpTALEN monomer was transiently expressed in HeLa cells. Two days after the transfection, Lv-mpTALEN (PL6LK)-ELD, Lv-mpTALEN(PL6WK)-ELD, Lv-mpTALEN(PLW6NM)-ELD, Lv-mpTALEN(JR)-ELD, and Lv-mpTALEN(JR)-KKR were stained with an anti-V5 antibody (red). Mitochondria were stained with an anti-TOM20 antibody (green). Nuclei were stained with DAPI (blue). Scale bar, 20 μm.

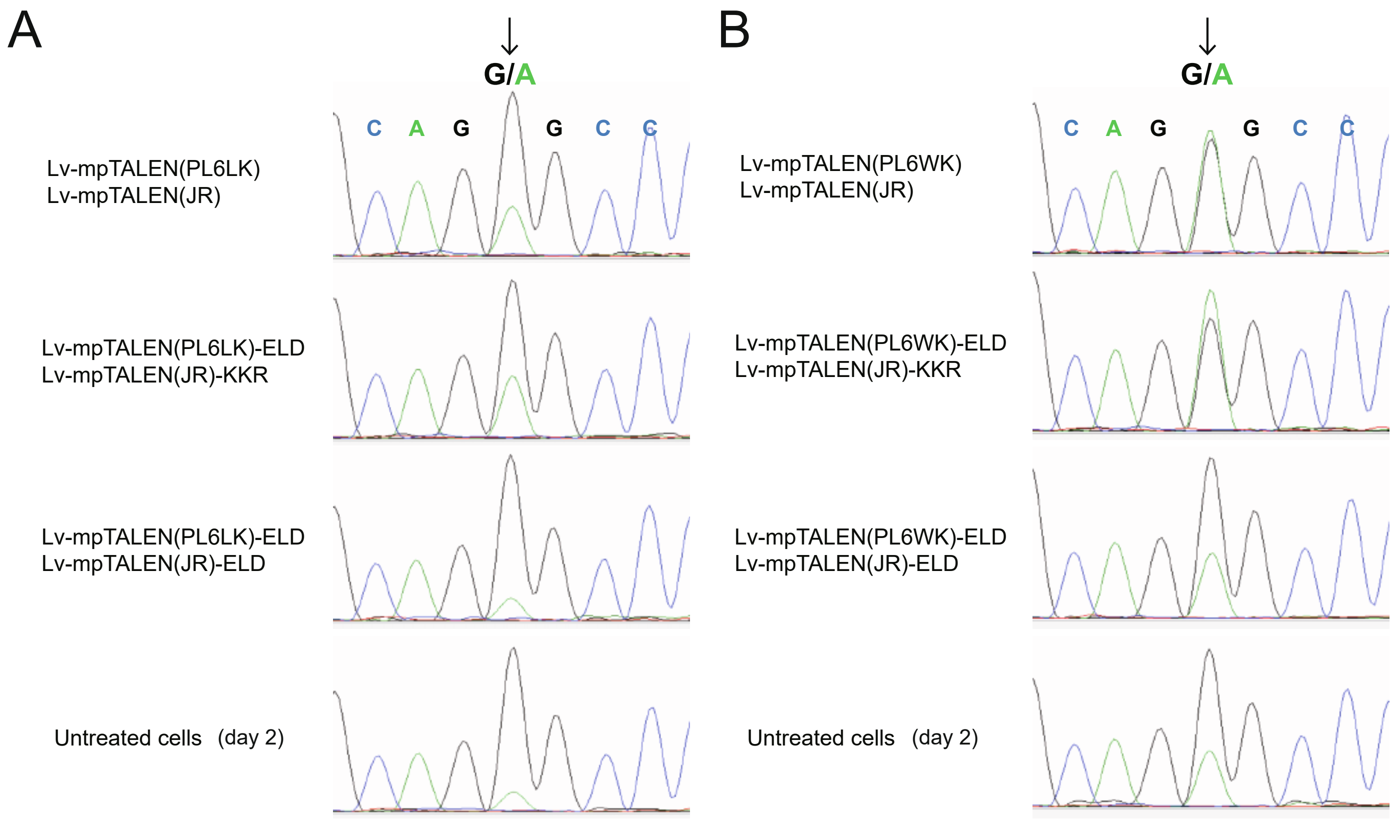

Figure S10

Sanger sequence analysis of m.3243A>G heteroplasmy in day 21 samples from Figures 3B and 3D data.

(A) Figure 3B and (B) FIgure 3D data. Arrow indicates the m.3243 position. The bottom data show electropherograms of untreated cells on day 2.

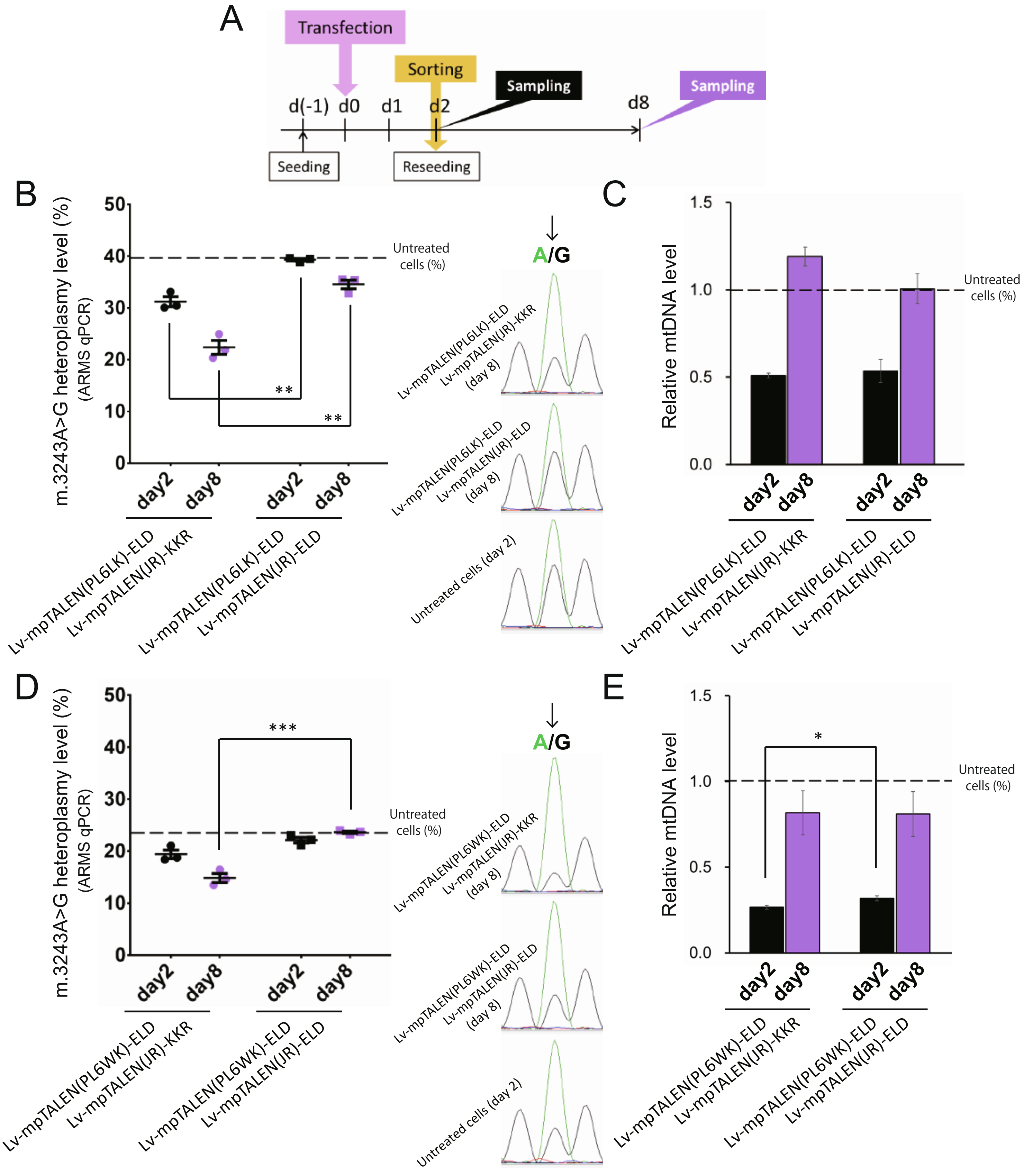

Figure S11

Effects of transient m.3243G(MUT)-mpTALEN expression on mtDNA heteroplasmy and copy numbers in #54D6-iPSCs.

(A) Experimental scheme. Two days after transfection, sorted cells were re-cultured without feeder cells for 6 days.

(B, D) Left: Heteroplasmy levels at 2 and 8 days after transfection. Dotted line indicates the heteroplasmy level in untreated cells at day 2. Data are expressed as the means ± SEM (n = 3). **$p < 0.01$, ***$p < 0.001$ (Holm-Sidak test). Right: Sanger sequencing data. Arrow indicates the m.3243 position.

(C, E) Relative mtDNA levels (correlating with mtDNA copy numbers) at 2 and 10 days after transfection. Data are presented as the ratio of the measured number to that in untreated cells at day 2 and expressed as the means ± SEM (n = 3). The dotted line indicates the relative copy number in untreated cells on day 2. *$p < 0.05$ (Holm-Sidak test).

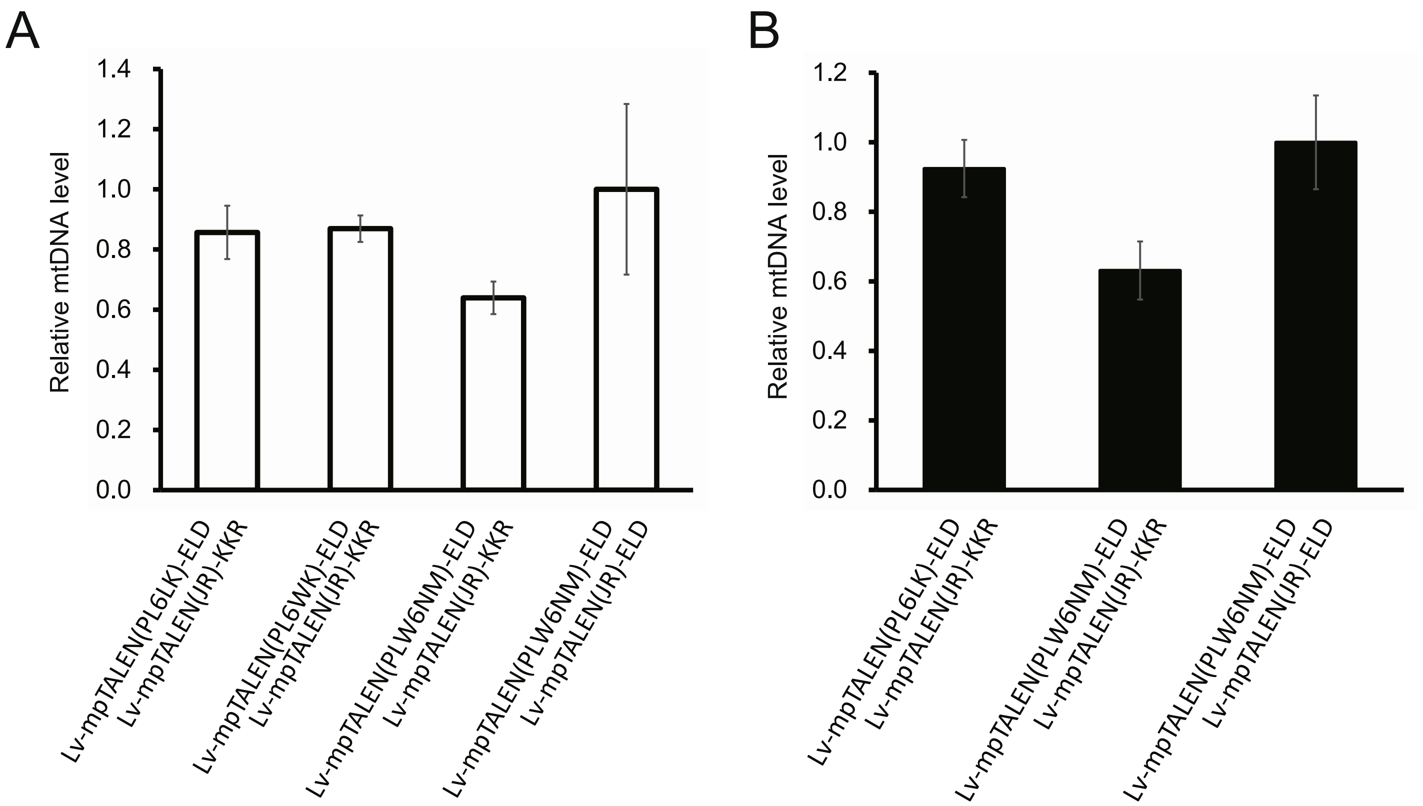

Figure S12

Effects of transient mpTALEN expression on mtDNA copy numbers in m.3243A(WT) homoplasmic cells.

(A) Relative mtDNA levels (correlating with mtDNA copy numbers) in sorted HEK293T cells at 2 day after transfection.

Data are presented relative to those of Lv-mpTALEN(PLW6NM)-ELD/Lv-mpTALEN(JR)-ELD and expressed as the mean ± SEM (n = 3).

There were no significant differences ($p > 0.05$, one-way ANOVA, followed by Tukey’ s multiple comparison test).

(B) Relative mtDNA levels (correlating with mtDNA copy numbers) in sorted A01#15_MyoD33-4 iPSCs at 2 day after transfection.

Data are presented relative to those of Lv-mpTALEN(PLW6NM)-ELD/Lv-mpTALEN(JR)-ELD and expressed as the means ± SEM (n = 3).

There were no significant differences ($p > 0.05$, one-way ANOVA, followed by Tukey’ s multiple comparison test).

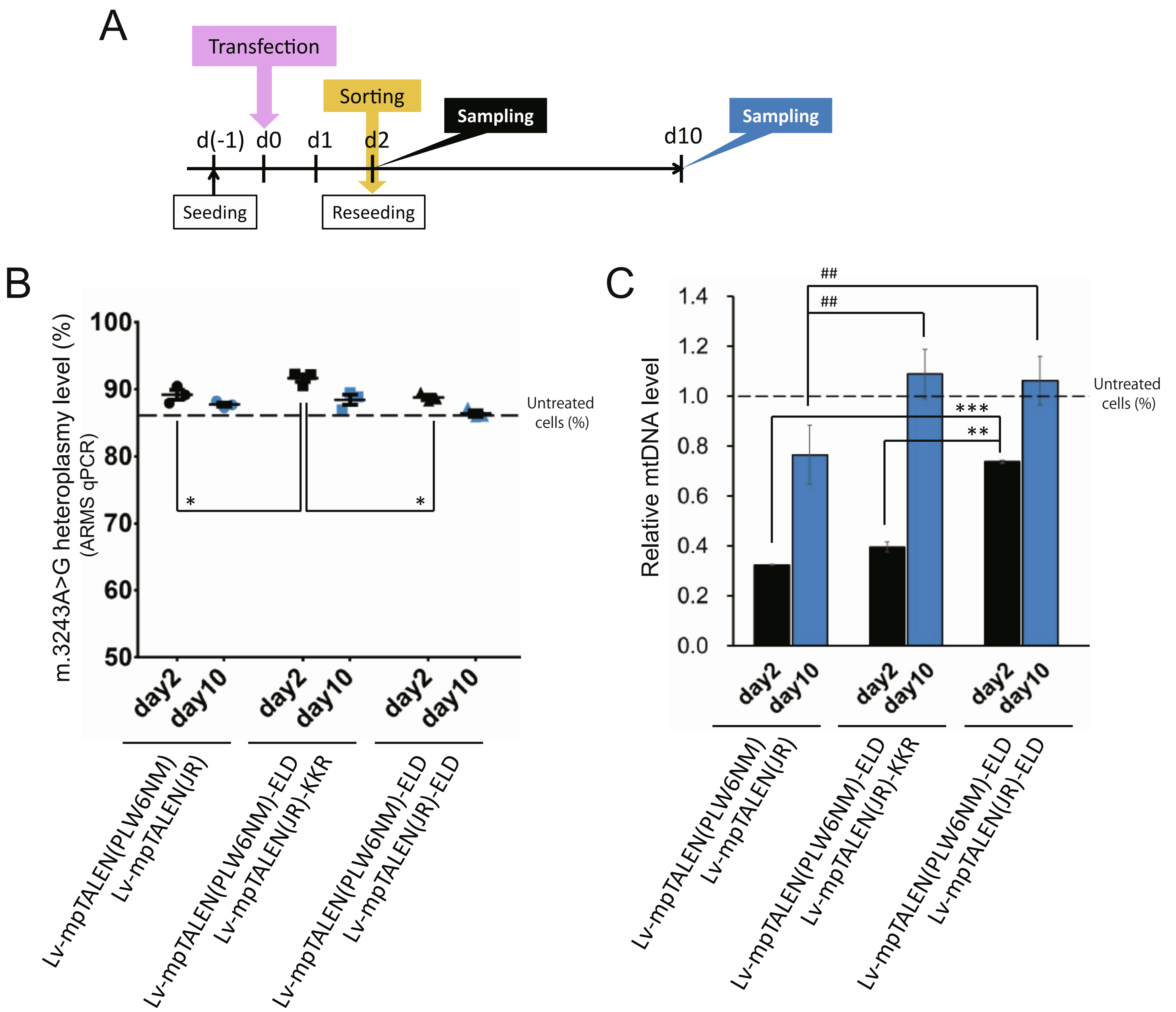

Figure S13

Effects of transient m.3243A(WT)-mpTALEN expression on mtDNA heteroplasmy and copy numbers in #54D6-iPSCs.

(A) Experimental scheme. Two days after transfection, sorted cells were re-cultured without feeder cells for 8 days.

(B) Heteroplasmy levels at 2 and 10 days after transfection. Dotted line indicates the heteroplasmy level in untreated cells at day 2. Data are expressed as the means ± SEM (n = 3). *p < 0.05 (two-way ANOVA, followed by Tukey' s multiple comparison test).

(C) Relative mtDNA levels (correlating with mtDNA copy numbers) at 2 and 10 days after transfection. Data are presented as the ratio of the measured number to that in untreated cells at day 2 and expressed as the means ± SEM (n = 3). The dotted line indicates the relative copy number in untreated cells on day 2. **, ## p < 0.01, ***p < 0.001 (two-way ANOVA, followed by Tukey' s multiple comparison test).

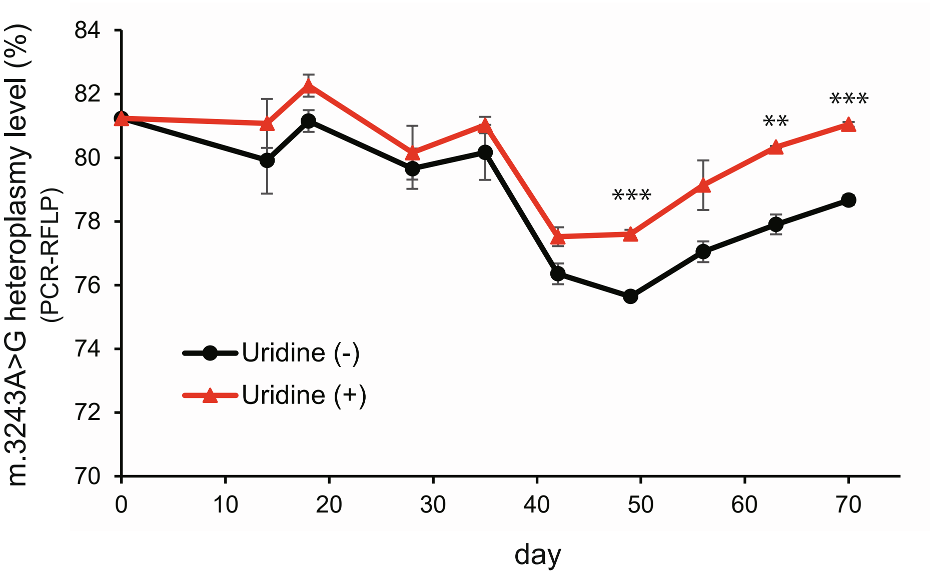

Figure S14

Effect of uridine supplementation on fluctuations of the m.3243A>G heteroplasmy levels in #54D6-iPSCs.

Long-term cultivation of #54D6-iPSCs was performed in feeder-free condition in the absence (black) or presence (red) of 50 μg/ml uridine. The m.3243A>G heteroplasmy levels were analyzed by PCR-RFLP at each passage point. Data are expressed as the means ± SEM of triplicate wells. Statistical significance was determined using the Holm-Sidak method for multiple comparisons. **$p < 0.01$, ***$p < 0.001$.

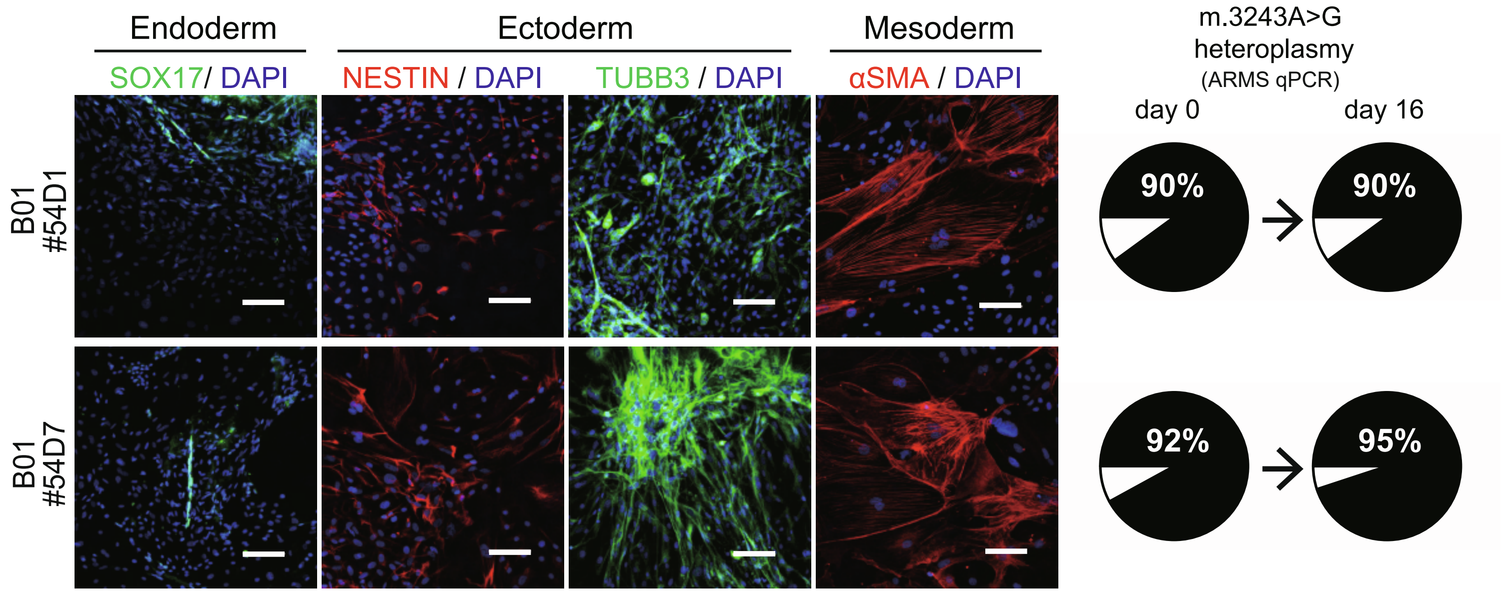

Figure S15

EB-mediated differentiation of #54D1- and #54D7-iPSCs with m.3243A>G mutation.

In vitro differentiation of two m.3243A>G-iPSC clones (B01_#54D1 and #54D7) into all three germ layers. Cells were labeled with anti-Sox17 (endoderm, green), anti-Nestin (ectoderm, red), anti-TUBB3 (ectoderm, green), and anti-αSMA (mesoderm, red) antibodies. Nuclei were stained with DAPI (blue). Scale bar, 100 μm. Pie charts at the right side show m.3243A>G heteroplasmy in undifferentiated iPSCs on day 0 and in differentiated cells on day 16, calculated by ARMS-qPCR.

Table S1: Primer list

Name	Sequence	Name	Sequence
Mito-1-2F	CATAGCACATTACAGTCAAATCCCTTCTCGTCCC	Mito-1-2R	ATTGCTAGGGTGGCGCTTCCAATTAGGTGC
mt3150-F	TACTTCACAAAGCGCCTTCC	mt3294-R	AGGAATTGAACCTCTGACTG
ACTB-F	CCAACCGCGAGAAGATGA	ACTB-R	TCCATCACGATGCCAGTG
rt-Oct3/4-F	GACAGGGGGAGGGGAGGAGCTAGG	rt-Oct3/4-R	CTTCCCTCCAACCAGTTGCCCCAAAC
rt-Nanog-F	CAGCCCCGATTCTTCCACCAGTCCC	rt-Nanog-R	CGGAAGATTCCCAGTCGGGTTCACC
rt-Sox2-F	GGGAAATGGGAGGGGTGCAAAAGAGG	rt-Sox2-R	TTGCGTGAGTGTGGATGGGATTGGTG
rt-Klf4-F	ACGATCGTGGCCCCGGAAAAGGACC	rt-Klf4-R	TGATTGTAGTGCTTTCTGGCTGGGCTCC
rt-L-Myc-F	GTGAGTCCCCCACCTGTAGA	rt-L-Myc-R	TTAGTAGCCAGTGAGGTATGCAATTC
rt c-Myc-F	GCGTCCTGGGAAGGGAGATCCGGAGC	rt c-Myc-R	TTGAGGGGCATCGTCGCGGGAGGCTG
rt-Lin28-F	CACCATGGGCTCCGTGTCCAACCAGCAG	rt-Lin28-R	TCAATTCTGTGCCTCCGGGAGCAGGGTAGG
MT-CYTB-F	TGCAACTATAGCAACAGCCTTCA	MT-CYTB-R	GAACTAGGTCTGTCCCAATGTATGG
FBXO15-F	GCCAGGAGGTCTTCGCTGTA	FBXO15-R	AATGCACGGCTAGGGTCAAA
pEP4-SF1	TTCCACGAGGGTAGTGAACC	pEP4-SR1	TCGGGGGTGTTAGAGACAAC
hFbx15-2F	GCCAGGAGGTCTTCGCTGTA	hFbx15-2R	AATGCACGGCTAGGGTCAAA
XmaI-A3243_F	TTAcccgggTATTATACCCACACCCACCC	XmaI-A3243AWT_R3	TTACCCGGGtgaacctctgactgtaaagttttaagttttatgcgattaccgggcT
		XmaI-A3243G_R3	TTACCCGGGtgaacctctgactgtaaagttttaagttttatgcgattaccgggcC
BamHI_Fok1_fwd	ACCAACAGAAGGATCCCCGA	K1-Fok1_rev	ATTTTCcttGACATAACGTTGCATCTC
K1-Fok2_fwd	TATGTCaagGAAAATCAGACCAGGAAC	R2K3-Fok2_rev	TTCGTctttctATTCAACCGTGTGAGCTG
R2K3-Fok3_fwd	GAATagaaagACGAACTGCAATGGTGCG	SexAI_Fok3_rev	ACTTTCCACACCTGGTTGCT
ARMS-A3243G(WT)_F1	CAGGGTTTGTTAAGATGGCAtA	ARMS-A3243G_R1	TGGCCATGGGTATGTTGTTA
ARMS-A3243G(MUT)_F1	CAGGGTTTGTTAAGATGGCAtG		
