# Supplementary material for: Optimization of mtDNA-targeted platinum TALENs for bi-directionally modifying heteroplasmy levels in patient-derived m.3243A>G-iPSCs
Source: Mol Ther Nucleic Acids. 2025 Mar 19;36(2):102521. doi: 10.1016/j.omtn.2025.102521 (PMC12002989; doi:10.1016/j.omtn.2025.102521)
Supplement: Document S2. Article plus supplemental information [file mmc2.pdf]

Molecular Therapy
Nucleic Acids
Original Article

Optimization of mtDNA-targeted platinum TALENs for bi-directionally modifying heteroplasmy levels in patient-derived m.3243A>G-iPSCs

Naoki Yahata,[1,2,3] Yu-ichi Goto,[4] and Ryuji Hata[1,5]

[1]Department of Anatomy I, Fujita Health University School of Medicine, Toyoake, Aichi 470-1192, Japan; [2]Department of Developmental Biology, Fujita Health University School of Medicine, Toyoake, Aichi 470-1192, Japan; [3]Division of Developmental Neurobiology, International Center for Brain Science, Fujita Health University, Toyoake, Aichi 470-1192, Japan; [4]Medical Genome Center, National Center of Neurology and Psychiatry, Kodaira, Tokyo 187-8551, Japan; [5]Osaka Psychiatric Research Center, Osaka Psychiatric Medical Center, Osaka Prefectural Hospital Organization, Hirakata, Osaka 573-0022, Japan

Patient-derived induced pluripotent stem cells (iPSCs) are a useful pathological model for debilitating diseases caused by mitochondrial DNA (mtDNA) mutations. We established iPSCs derived from mitochondrial disease patients, heteroplasmic for the m.3243A>G mutation. The proportion of a selected mtDNA can be reduced by delivering a programmable nuclease into the mitochondria, and we developed various mtDNA-targeted Platinum TALENs (mpTALENs) to modify m.3243A>G-iPSC heteroplasmy levels in either wild-type or mutant direction. For TALEN optimization, the use of non-conventional repeat-variable di-residues (ncRVD)—LK/WK or NM—enhanced cleavage activity and specificity, and the replacement of conventional with obligate heterodimeric FokI nuclease domains increased target specificity and protected mtDNA from copy number depletion. *In vitro*, depending on whether wild-type or mutant mtDNA was targeted, we could obtain m.3243A>G-iPSCs with a higher or lower mutation load, while the cells retained their ability to differentiate into three germ layers. These results demonstrate that our mpTALEN optimization created a useful tool for altering heteroplasmy levels in m.3243A>G-iPSCs, improving the potential for studying mutation pathology. The enhanced efficiency also holds promise for using m.3243G(MUT)-mpTALEN as a therapeutic strategy for treating patients suffering from m.3243A>G mitochondrial diseases.

INTRODUCTION

Understanding and manipulating mitochondrial DNA (mtDNA) mutations are crucial for developing therapeutic strategies for mitochondrial diseases. Challenges include the presence of multiple copies of both mutant and wild-type mtDNA per cell (“heteroplasmy”), with variation in their ratios,[1] and the double mitochondrial membrane, which poses a significant barrier to oligonucleotides used in gene manipulation strategies like CRISPR. Our study aimed to optimize mtDNA-targeted platinum transcription activator-like effector nucleases (mpTALENs) to efficiently and safely alter heteroplasmy levels in patient-derived induced pluripotent stem cells with m.3243A>G mutation (m.3243A>G-iPSCs), thereby advancing the research and treatment of mitochondrial disorders.

The m.3243A>G mutation is among the most frequently observed mtDNA mutations in patients with mitochondrial diseases. This mutation in the tRNA$^{Leu(UUR)}$ gene of mtDNA is associated with various clinical presentations, such as mitochondrial myopathy, encephalopathy, lactic acidosis, and stroke-like episodes (MELAS) and diabetes mellitus.[2,3] Most patients with the m.3243A>G mutation possess both normal and mutant mtDNA within single cells, and this heteroplasmy is also referred to here as “m.3243A>G.” The diversity in the severity and clinical phenotypes of the disease is not fully understood but is thought to correlate with the level of heteroplasmy and its distribution among the patient’s tissues.[1]

There are no good animal models for m.3243A>G-related disease. However, valuable research tools have been derived from patients with m.3243A>G mutation, including fibroblasts, myoblasts, established cybrid cells, and induced pluripotent stem cells (iPSCs), which have expanded the possibilities of disease modeling.[4]

In recent years, tools for manipulating mtDNA mutations have rapidly developed.[5] Programmable nucleases such as mitochondrially targeted-zinc finger nuclease (mtZFN), mitoTALEN, and mitoARCUS have shown the ability to alter mutation load in heteroplasmic cells and tissues by introducing double-strand breaks (DSBs) into mutant mtDNA.[6–10] Additionally, mtDNA base editing strategies that do not introduce DSBs have been developed,[11–13] though their abilities for making targeted changes are currently limited.[14]

A few reports have demonstrated that mito-nucleases or mito-base editors can reduce the mutation load in mitochondrial disease

Received 16 July 2024; accepted 16 March 2025;
https://doi.org/10.1016/j.omtn.2025.102521.

Correspondence: Naoki Yahata, Department of Developmental Biology, Fujita Health University School of Medicine, Toyoake, Aichi 470-1192, Japan.
E-mail: n-yahata@fujita-hu.ac.jp
Correspondence: Ryuji Hata, Osaka Psychiatric Research Center, Osaka Psychiatric Medical Center, Osaka Prefectural Hospital Organization, Hirakata, Osaka 573-0022, Japan.
E-mail: hata@fujita-hu.ac.jp

© 2025 The Author(s).
Published by Elsevier Inc. on behalf of The American Society of Gene and Cell Therapy.
This is an open access article under the CC BY license (http://creativecommons.org/licenses/by/4.0/).

patient-derived iPSCs with mtDNA mutation, such as m.13513G>A,[15,16] m.3243A>G,[17] and m.4300A>G.[18] Previously, when targeting another mutant mtDNA, we demonstrated the possibility of using mpTALENs that are relatively small by having fewer than usual repeat-variable di-residues (RVDs) units in the TALE array in order to facilitate their therapeutic delivery.[16] In the present study, for the creation of m.3243G(MUT)-mpTALENs, we applied this size principle when searching for the best possible TALE target regions, and additionally introduced novel non-conventional RVDs (ncRVDs) for optimizing target recognition[19] and obligate heterodimeric FokI domains for reducing off-target cleavage by homodimeric TALEN pairs.[20–22] This strategy was also applied for developing mpTALENs that preferentially cleave wild-type mtDNA (m.3243A(WT)-mpTALENs).

Our data represent progress toward the ultimate aim to cure m.3243A>G disease, and the bi-directionality of our system facilitates the creation of iPSCs with varying levels of m.3243A>G heteroplasmy for research purposes.

RESULTS

Development of m.3243G(MUT)-pTALEN and m.3243A(WT)-pTALEN with ncRVDs

In this study, we aimed to develop TALEN pairs that effectively change the m.3243A>G heteroplasmy levels using the Platinum TALEN system.[23] We designed m.3243G(MUT)-pTALENs with the intent that the TALENs preferentially bind the mutant sequence including m.3243G and induce double-strand breaks (DSBs) in mutant mtDNA. To evaluate the activity and specificity of the designed m.3243G (MUT)-pTALENs, we established a mammalian cell-based single-strand annealing (SSA) assay[24] using a reporter plasmid carrying a fragment of the mtDNA sequence, including either m.3243A(WT) or m.3243G(MUT) (Figure S1A). We evaluated various types of pTALEN pairs to fit the condition that immediately upstream of the DNA sequence, recognized by the RVDs of the pTALEN monomer (position 0), was a "T"[25,26] (Figure S1B). The majority of the investigated m.3243G(MUT)-pTALEN pairs induced efficient mtDNA-cleaving activity, but only the pairs including the pTALEN(FR) monomer were quite specific for cleaving the mutant mtDNA (Figure S1C). The highest m.3243G(MUT) target specificity was observed for the pTALEN(BL)/pTALEN(FR) pair (abbreviated as p[BL/FR]) (Figure S1D).

Next, we attempted to modify the RVD in m.3243G(MUT)-pTALENs that recognizes the m.3243G nucleotide, referring to previous research on improving the TALEN specificity for a nuclear genome sequence using non-conventional RVDs (ncRVDs).[19] The pTALEN(MR) as right-pTALEN and pTALEN(CL) and pTALEN(PL) as left-pTALEN were designed, together with variants of the left-TALENs in which the conventional NN of the sixth RVD was replaced by the non-conventional LK (Figure S2A). These modified TALENs are named pTALEN(CL6LK) and pTALEN(PL6LK) (Figure S2A), and the LK for NN change is expected to confer a higher specificity for binding G over A. A comparative study using an SSA assay revealed that p[CL6LK/MR] and p[PL6LK/MR] possess a higher cleavage activity and specificity for mutant types than p[CL/MR], and p[PL/MR], respectively (Figures S2B and S2C). The cleavage activity of p[PL6LK/MR] against the mutant sequence was approximately twice that of p[BL/FR] (Figure S2B).

We additionally investigated the utility of ncRVDs other than LK for recognizing the m.3243G nucleotide and created variants of pTALEN(PL6LK) in which the 12th amino acid of the sixth RVD was substituted from L to W, F, or N (Figure 1A). These left-pTALENs were evaluated for SSA activity in combination with multiple right-TALENs: pTALEN(JR), pTALEN(OR), and pTALEN(QR), each slightly differing in TALE array length and target binding position (Figures 1A and S2D). The p[PL6LK/JR] pair showed the most favorable combination of a high cleavage activity (Figures 1B and S2E) and target specificity for the mutant sequence (Figures 1C and S2F). The p[PL6WK/JR] pair was selected as the second candidate because it had a higher cleavage activity for the mutant sequence compared with the p[PL6LK/OR] and p[BL/FR] pairs (Figures 1B, S2B, and S2E), which showed a higher target specificity (Figures 1C, S2C, and S2F).

Next, we aimed to develop m.3243A(WT)-pTALEN, which preferentially targets and digests mtDNA with wild-type m.3243A. Using the same strategy for finding m.3243G(MUT)-pTALEN pairs, we designed the following left-pTALENs: pTALEN(PLW), pTALEN (PLW6HY), and pTALEN(PLW6NM), in which the sixth RVD recognizing the m.3243A nucleotide was NI, HY, and NM, respectively (Figure S3A). The SSA assay results indicated that p[PLW6HY/MR] and p[PLW6NM/MR] had a higher cleavage activity for the wild-type sequence than p[PLW/MR] (Figure S3B), with p [PLW6NM/MR] exhibiting a slightly higher target specificity (Figure S3C). Therefore, we evaluated the combination of pTALEN(PLW6NM) and several right-TALENs (Figures 1D and S3D). The cleavage activity for the m.3243A(WT) containing sequence decreased in the order: p[PLW6NM/QR] > p[PLW6NM/MR] > p [PLW6NM/JR] > p[PLW6NM/OR] (Figures 1E and S3E). However, we selected p[PLW6NM/QR] and p[PLW6NM/JR] as the two candidates for m.3243A(WT)-pTALEN because their target specificities for wild-type over mutant sequences were considerably higher (Figures 1F and S3F).

Generation and characterization of iPSCs from mitochondrial disease patients with m.3243A>G mutation

We reprogrammed fibroblasts from two mitochondrial disease patients (named B01 and A04), heteroplasmic for m.3243A>G mtDNA, and picked and expanded four and six iPSC clones, respectively. There was no integration of episomal vectors for reprogramming in these iPSC clones, as indicated by negative results for the OriP region using genomic PCR (Figure S4A). The heteroplasmy levels of m.3243A>G in these iPSC clones, estimated by the PCR-RFLP method, varied (Figure S4B).

For further studies, we selected three B01 clones with a high proportion and one A04 iPSC with a moderate proportion of m.3243A>G mutant mtDNA. These iPSC clones showed human ESC-like morphology and a normal karyotype (Figure S5A), and they were

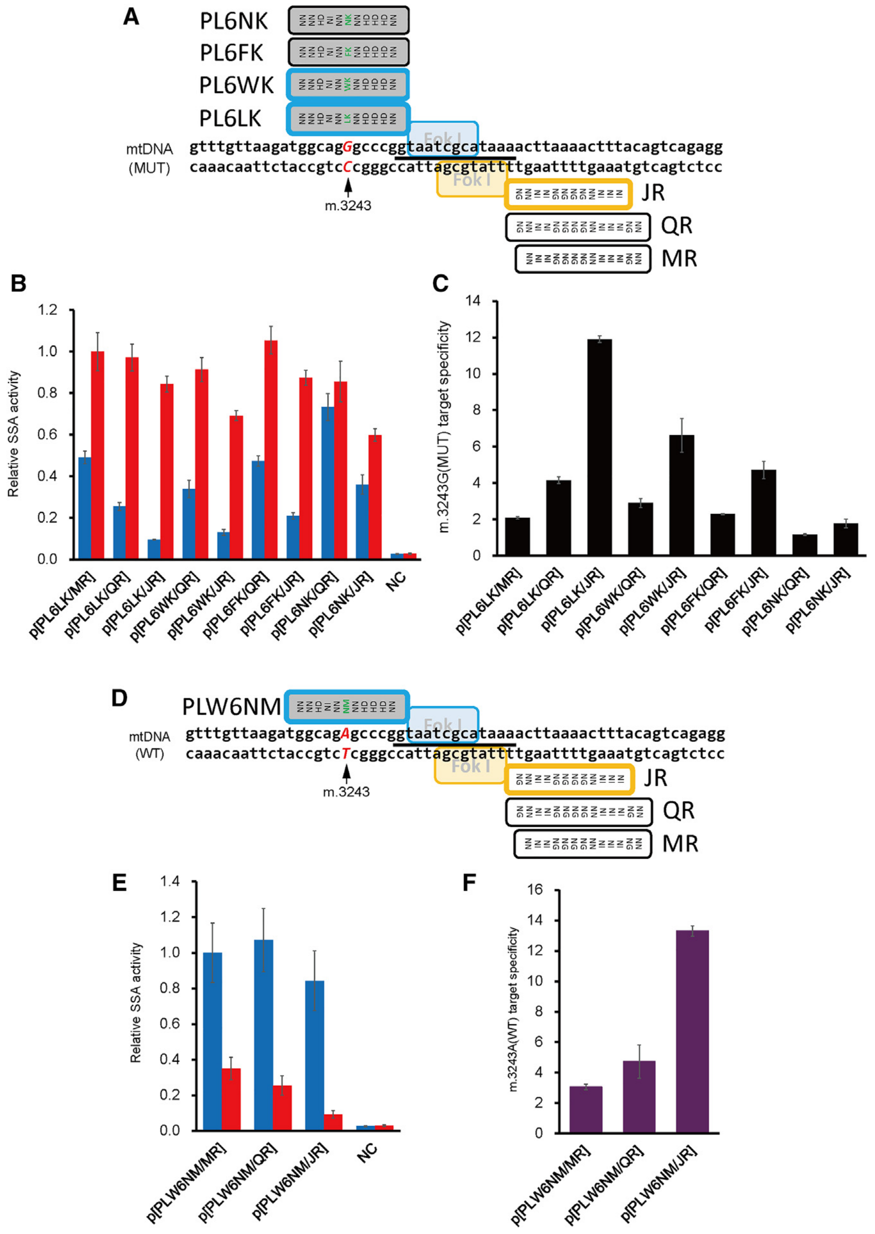

Figure 1. Functional evaluation of engineered m.3243G(MUT)- and m.3243A(WT)-pTALENs with ncRVDs using an SSA assay

(A, D) Schematic illustration of the target sites of m.3243G(MUT)-pTALENs (A) and m.3243A(WT)-pTALENs (D). Gray and white boxes indicate left-pTALENs and right-pTALENs, respectively, with their names written beside them. The black bars indicate the spacer regions bracketed by the pTALEN(PL6LK)/pTALEN(JR) pair, abbreviated as p[PL6LK/JR] (A) and p[PLW6NM/JR] (D), respectively. Green letters indicate ncRVDs. (B, E) Evaluation of the relative SSA activity (Luc/RLuc) of pTALEN pairs. Blue and red bars indicate cleaving activities against human mtDNA sequences, including m.3243A(WT) and m.3243G(MUT), respectively. Relative SSA activity is defined as the ratio of the measured activity to the activity score of p[PL6LK/MR] against mutant mtDNA sequence (B), and p[PLW6NM/MR] against wild-type mtDNA sequence (E), respectively. Data are expressed as the mean ± SEM (n = 3). NC, negative control. (C, F) Specificity of each pTALEN pair toward the m.3243G(MUT) (C) and m.3243A(WT) (F) sequences, respectively. Data are expressed as the means ± SEM (n = 3).

positive for pluripotency markers, as demonstrated by immunocytochemistry for Oct4, SSEA-4, and NANOG (Figure S5B) and by reverse transcription-PCR (RT-PCR) for *Oct4*, *Nanog*, *Sox2,* and *Lin28* (Figure S5C).

m.3243G(MUT)-mpTALEN and m.3243A(WT)-mpTALEN can change heteroplasmy levels in m.3243A>G-iPSCs

Selected left- and right-pTALENs were modified into "Lv-mpTALENs" for mitochondrial localization, by adding a mitochondrial targeting sequence (MTS) from ATP5B and a V5-tag at the N terminus.[16] To examine whether Lv-mpTALENs are readily imported into mitochondria, we analyzed their subcellular localization in transiently expressing HeLa cells. Immunofluorescence analysis revealed that mpTALENs colocalized with the mitochondrial marker Tom20 and were not observed in the nucleus (Figure S6).

We evaluated the efficacy of the two m.3243G(MUT)-mpTALEN pairs in altering the mtDNA heteroplasmy level in iPSCs with the m.3243A>G mutation. In heteroplasmic iPSC populations, the mutation load can considerably vary between iPSCs.[27] To avoid biasing the mutation load, before mpTALEN application, the iPSCs were separated as single cells, seeded on plate sparsely, and cultured under feeder-free conditions. The Lv-mpTALEN(PL6LK)/Lv-mpTALEN(JR) or Lv-mpTALEN(PL6WK)/Lv-mpTALEN(JR) plasmids were introduced into one of the B01 m.3243A>G-iPSC clones, #54D6-iPSC. EGFP was expressed from a co-transfected plasmid as a transfection marker. On day 2 after transfection, EGFP-positive and live (propidium iodide-negative) cells were sorted using a cell sorter (Figure S7A). Heteroplasmy levels in sorted iPSCs, estimated using PCR-RFLP, were compared with "mock-treated" controls, sorted cells transfected with twice the amount of the Lv-mpTALEN(JR) plasmid (Figure S7B). The expression of the Lv-mpTALEN(PL6LK)/Lv-mpTALEN(JR) pair (abbreviated as mp[PL6LK/JR]) or mp[PL6WK/JR] decreased the m.3243A>G heteroplasmy levels in #54D6-iPSCs compared with that in cells transfected for Lv-mpTALEN(JR) only (Figure S7B). There was no significant difference between mp[PL6LK/JR] and mp[PL6WK/JR] in their effects on

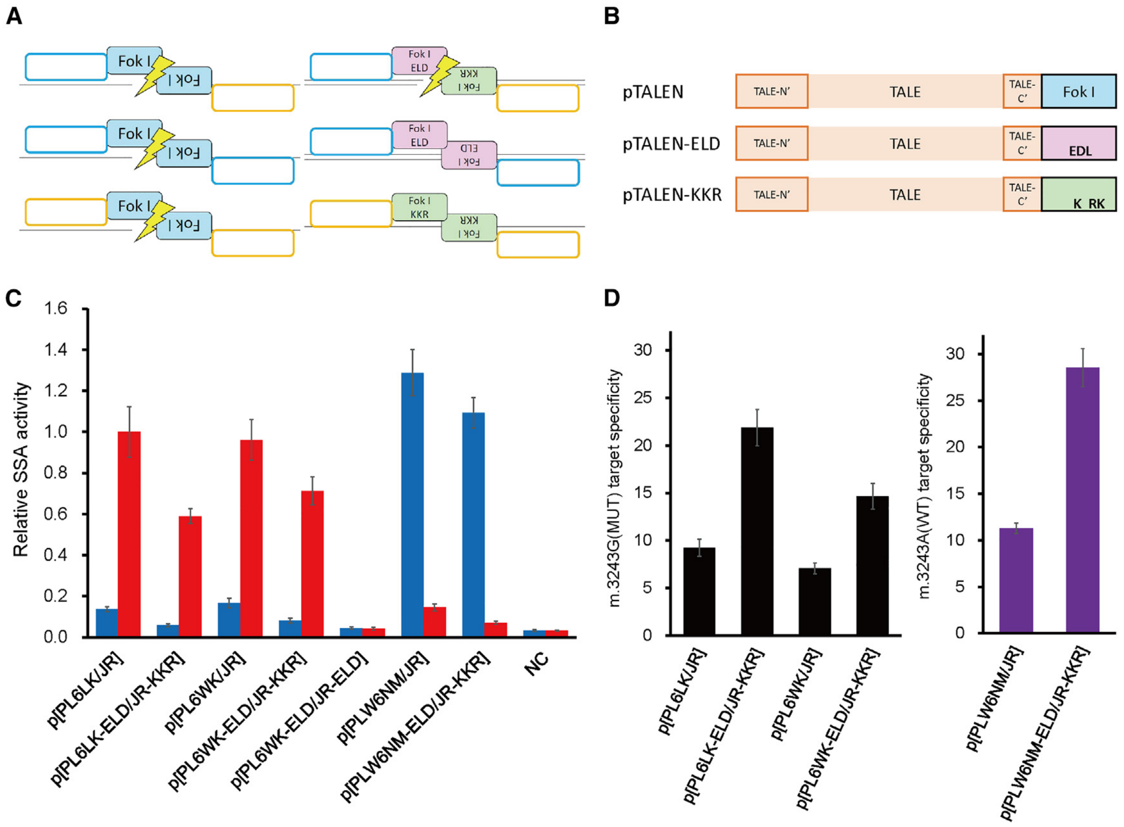

Figure 2. Functional comparisons of pTALENs with homodimeric and heterodimeric FokI

(A) Schematic of possible TALEN pairing combinations. Coexpression of left-pTALEN and right-pTALEN yields a heterodimer that cleaves the desired target (upper left) but also yields homodimers that may cleave other targets (middle left and lower left). Variations of the FokI cleavage domain that function as obligate heterodimers labeled "ELD" and "KKR" would enable cleavage of desired heterodimer targets (upper right) while eliminating off-target activity by homodimer species (middle right and lower right). (B) Domain structure of original and heterodimeric pTALENs. Original pTALENs contain a wild-type FokI domain. The monomers of obligate heterodimeric pTALEN pairs each contain a different mutated FokI domain, ELD and KKR, respectively, and are named pTALEN-ELD and pTALEN-KKR. (C) Evaluation of the relative SSA activity (Luc/RLuc) of pTALEN pairs. Blue and red bars indicate cleaving activities against human mtDNA sequences, including m.3243A(WT) and m.3243G(MUT), respectively. Relative SSA activity is defined as the ratio of the measured activity to the activity score of p[PL6LK/JR] against mutant mtDNA sequence. Data are expressed as the mean ± SEM (n = 3). NC, negative control. (D) Specificity of each pTALEN pair toward the m.3243G(MUT) (left) and m.3243A(WT) (right) sequences, respectively. Data are expressed as the means ± SEM (n = 3).

altering the mutation load in iPSCs on day 2 (Figure S7B), despite some differences in effects observed between p[PL6LK/JR] and p[PL6WK/JR] in the SSA assay (Figures 1B and 1C).

We similarly evaluated the efficacy of the two selected m.3243A(WT)-mpTALEN pairs in altering the mtDNA heteroplasmy level in #50V4-iPSCs. The data showed that the expression of mp[PLW6NM/JR] or mp[PLW6NM/QR] increased the percentage of m.3243A>G mutant mtDNA in #50V4-iPSCs compared with that in cells transfected for Lv-mpTALEN(JR) only (Figure S7D). As expected from the results of the SSA assay (Figures 1E and 1F), mp[PLW6NM/JR] was more effective than mp[PLW6NM/QR] in increasing the mutation load (Figure S7D).

No differences were observed in relative mtDNA levels on day 2 after transfection when comparing between m.3243G(MUT)-mpTALEN, m.3243A(WT)-mpTALEN, and twice the amount of Lv-mpTALEN (JR) (Figures S7C and S7E). These results suggest that the effects of the designed mpTALEN pairs on reducing mtDNA copy number by cutting the target DNA are low and cells may have partially compensated for that by mtDNA replication.

Evaluations of obligate heterodimeric m3243G(MUT)- and m.3243A(WT)-pTALENs

Previous research demonstrated that obligate heterodimeric FokI should reduce unwanted homodimeric pairing between two identical TALEN molecules.[20,21] The heterodimeric FokI domains referred to as ELD/KKR[22] cannot cleave DNA when they form homodimers with ELDs or KKRs (Figure 2A). We introduced the ELD and KKR mutations into the FokI domains of selected left-pTALENs and right-pTALENs, creating pTALEN-ELD and pTALEN-KKR, respectively (Figure 2B). The results of the SSA assay showed that this

introduction into selected m.3243G(MUT)- and m.3243A(WT)-pTALEN pairs caused a moderate decrease in cleavage activity in all cases (Figure 2C). Although it is not possible to pinpoint the exact reasons for the decrease in DNA cleavage activity of the pTALEN-ELD/pTALEN-KKR pair, we speculate that it involved a reduction in off-target mtDNA cleavage caused by homodimeric FokI.

The reduction in cleavage activity was more pronounced for the mutant or wild-type variant that differed from the intended target sequence, and, as a result, for each of the two investigated m.3243G(MUT)-pTALEN pairs and single m.3243A(WT)-pTALEN pair, the introduction of heterodimer FokI increased the target specificity (Figure 2D). In addition, the selected m.3243A(WT)-pTALEN showed higher DNA cleavage activity and target specificity than m.3243G(MUT)-pTALENs even when compared under the same conditions in the SSA assay (Figures 2C and 2D). As a negative control, the p[PL6WK-ELD/JR-ELD] pair using pTALEN(JR) with an ELD mutation in both TALENs of the pair, did not show cleavage activity as expected (Figure 2C).

In order for these heterodimeric pTALENs to function in mitochondria, they were modified into Lv-mpTALENs with MTS and V5-taq sequences as described above. Similar to the conventional Lv-mpTALENs, these heterodimeric monomers were detected by western blotting using an anti-V5 antibody (Figure S8) and their localization within mitochondria was supported by immunocytochemistry (Figure S9).

Long-term effects of heterodimeric m.3243G(MUT)-mpTALEN application on m.3243A>G-iPSCs

We monitored mtDNA heteroplasmy and relative mtDNA levels (a measure for mtDNA copy numbers) during long-term cultivation of m.3243G(MUT)-mpTALEN-transfected #54D6-iPSCs. At 2 days post-transfection, transfected cells were sorted and reseeded together on iMatrix-511-coated plates and cultured and passaged as a mixed culture for an additional 19 days, during which samples were taken for analysis (Figure 3A). To obtain a more accurate assessment of the heteroplasmy levels, the allele refractory mutation system-based quantitative real-time PCR (ARMS-qPCR) method[28] was also used. The heteroplasmy-shifting effects of the Lv-mpTALEN(PL6LK)-ELD/Lv-mpTALEN(JR)-KKR pair (abbreviated as mp[PL6LK-ELD/JR-KKR]) were stronger than those of the mp[PL6LK/JR] pair at 2, 10, and 21 days following transfection (Figures 3B and S10A). The heteroplasmy levels in these mpTALEN-applied iPSCs gradually decreased during 19 days of cultivation (Figure 3B). Similarly, the mp[PL6WK-ELD/JR-KKR] pair was more effective in reducing the percentage of mutant mtDNA than mp[PL6WK/JR] (Figures 3D and S10B). Additionally, we prepared mp[PL6LK-ELD/JR-ELD] and mp[PL6WK-ELD/JR-ELD] pairs as negative controls, and, as expected, they did not induce changes in heteroplasmy (Figures 3B, 3D, and S10). On day 2, the cells transfected for mp[PL6LK-ELD/JR-KKR] or mp [PL6WK-ELD/JR-KKR] showed a more moderate reduction of the relative mtDNA levels than those transfected for mp[PL6LK/JR] or mp [PL6WK/JR], respectively, although the reductions were higher than for the respective ELD/ELD control (Figures 3C and 3E). As reported in previous studies, the mtDNA depletion caused by mtDNA-targeted nucleases gradually recovered over time (as time allows for mtDNA replication).[6,16,29] Namely, in all cases the relative mtDNA levels gradually increased in the mpTALEN-transfected cells and within 19 days reached a level close to that observed in untreated cells on day 2 (Figures 3C and 3E). Given that these heterodimeric mpTALEN pairs not only demonstrated higher mutation load reduction effects but also suppressed mtDNA copy number depletion, we considered them the most appropriate m.3243G(MUT)-mpTALENs to decrease the m.3243A>G heteroplasmy level in m.3243A>G-iPSCs. Furthermore, we introduced m.3243G(MUT)-mpTALENs into #54D6-iPSCs with a heteroplasmy level of approximately 40% to further reduce the percentage of mutant mtDNA (Figure S11). Ultimately, we were able to obtain #54D6-iPSCs with a heteroplasmy level of 11% (Figure 4).

Off-target mtDNA cleavage of heterodimeric m.3243G(MUT)-mpTALENs

The heterodimeric m.3243G(MUT)-mpTALENs selected in this study successfully reduced the mutation load. However, in the context of therapeutic applications, it is also desirable that the activity to cleave normal mtDNA is minimized as much as possible. Therefore, using human cells without m.3243A>G mutant mtDNA, we evaluated the residual activity of cleaving normal mtDNA by comparing the mtDNA copy number. The mp[PLW6NM-ELD/JR-KKR] pair was used as a positive control for cleaving normal mtDNA, and the mp[PLW6NM-ELD/JR-ELD] pair as a negative control. The mp [PL6LK-ELD/JR-KKR] or mp[PL6WK-ELD/JR-KKR] pair was introduced into HEK293T cells, and the copy number on day 2 post-transfection was compared with the negative control. Although the difference was not significant, the mp[PL6LK-ELD/JR-KKR] or mp[PL6WK-ELD/JR-KKR] pair showed a slight decrease in mtDNA copy number compared with the negative control (Figure S12A). Furthermore, the mtDNA copy number in mp[PL6LK-ELD/JR-KKR]-treated iPSCs without m.3243A>G mutant mtDNA (A01#15_MyoD33-4[16]) showed a similar trend (Figure S12B). These data suggest that the two pairs of heterodimeric m.3243G(MUT)-mpTALENs still have a slight off-target effect causing cleavage of normal mtDNA.

Long-term effects of heterodimeric m.3243A(WT)-mpTALEN application on m.3243A>G-iPSCs

We verified the effect of m.3243A(WT)-mpTALEN on increasing the mutation load in heteroplasmic #54D6-iPSCs (Figure S13A). The obligate heterodimeric mp[PLW6NM-ELD/JR-KKR] pair was able to increase the mutation load up to 92% on day 2, but the original mp[PLW6NM/JR] pair failed to induce a significant increase in the original high mutation load (89%) in these #54D6-iPSCs (Figure S13B). Unfortunately, by day 10, the percentage of mutant mtDNA had dropped back to near its original level in each case (Figure S13B). In conclusion, also for m.3243A(WT)-mpTALEN, the usage of obligate heterodimeric FokI domains enhanced the heteroplasmy-shifting effect, but the effect was small and not sustained. Data indicated that the mtDNA copy number depletion was slightly

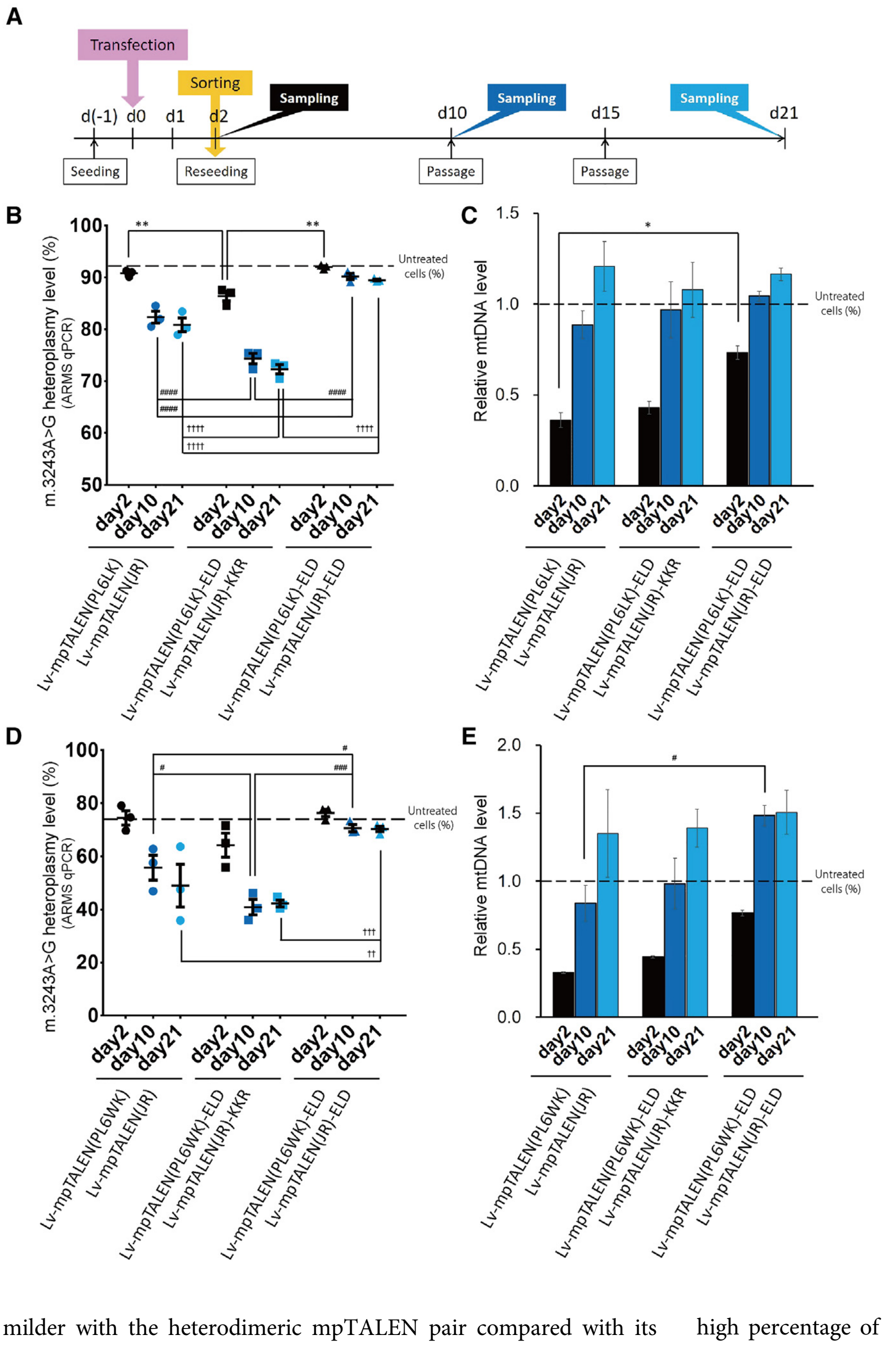

Figure 3. Long-term effects of heterodimeric m.3243G(MUT)-mpTALEN expression on mtDNA heteroplasmy and copy numbers in #54D6-iPSCs

(A) Experimental scheme. Two days after transfection, sorted cells were re-cultured without feeder cells for 19 days. (B, C) Evaluation of the Lv-mpTALEN(PL6LK)/Lv-mpTALEN(JR) pair. (D, E) Evaluation of the Lv-mpTALEN(PL6WK)/Lv-mpTALEN(JR) pair. (B, D) Heteroplasmy levels at 2, 10, and 21 days after transfection. Dotted lines indicate the heteroplasmy level in untreated cells on day 2. Data are expressed as the means ± SEM (n = 3). $^{\#}p < 0.05$, $^{**,\ \dagger\dagger}p < 0.01$, $^{\#\#\#,\ \dagger\dagger\dagger}p < 0.001$, $^{\#\#\#\#,\ \dagger\dagger\dagger\dagger}p < 0.0001$ (two-way ANOVA, followed by Tukey's multiple comparison test). (C, E) Relative mtDNA levels (correlating with mtDNA copy number) at 2, 10, and 21 days after transfection. Data are presented as the ratio of the measured number to that in untreated cells on day 2 and expressed as the means ± SEM (n = 3). The dotted lines indicate the relative mtDNA level in untreated cells on day 2. $^{*,\ \#}p < 0.05$ (two-way ANOVA, followed by Tukey's multiple comparison test).

milder with the heterodimeric mpTALEN pair compared with its precursor, and the mtDNA copy number recovery by day 10 correlated with the copy number on day 2 (Figure S13C). The combined results (Figures S13B and S13C) indicated that a change in protocol was needed to establish and maintain iPSCs with a higher mutation load.

Sorted iPSCs were sowed on feeder layers and cultured using Primate ES Cell Medium (ReproCELL) (Figure 5A). The mp[PLW6NM-ELD/JR-KKR]-applied #50V4-iPSCs, which originally had an approximately 75% mutation load, exhibited about 11% higher heteroplasmy levels compared with the mp[PLW6NM-ELD/JR-ELD]-applied controls on day 2 (Figure 5B). However, the differences in heteroplasmy levels between mp[PLW6NM-ELD/JR-KKR]-applied and mp[PLW6NM-ELD/JR-ELD]-applied cells exhibited a decreasing trend after 25 days (Figure 5B). These data demonstrate that it is challenging to stably culture mp[PLW6NM-ELD/JR-KKR]-applied m.3243A>G-iPSCs with a higher mutation load.

Uridine, a pyrimidine nucleoside, plays an important role in cellular metabolism and is supplemented in the culture of cell models with mtDNA mutations, such as cybrids[30] and patient-derived cells,[31] to maintain cell growth by compensating for the functional depletion of mitochondrial respiratory activity.[32] We examined the effect of uridine supplementation on heteroplasmy levels in #54D6-iPSCs with a high percentage of the m.3243A>G mutation in feeder-free conditions using StemFit AK02N medium (Ajinomoto). The results indicated that uridine supplementation reduced the tendency of the mutation load in #54D6-iPSCs to decrease over time, although fluctuations in loads persisted (Figure S14).

We again introduced m.3243A(WT)-mpTALEN into #54D6-iPSCs to try to increase the mutation load. After cell sorting, the cells were seeded on a feeder layer with uridine supplementation (Figure 6A). As expected, the mutation load in mp[PLW6NM-ELD/JR-KKR]-applied cells was higher than that in mp[PLW6NM-ELD/JR-ELD]-applied cells

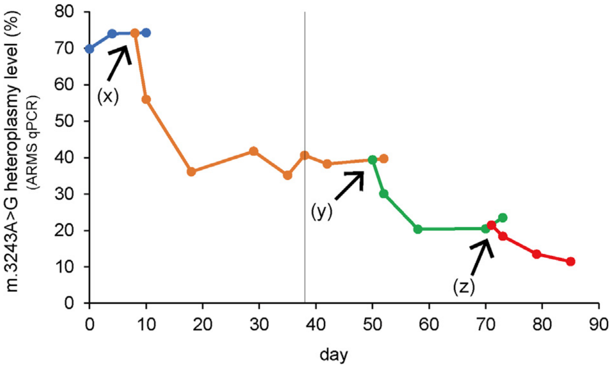

Figure 4. An example of m.3243A>G heteroplasmy shifts in #54D6-iPSCs upon iterative expression of m.3243G(MUT)-mpTALENs

Arrows indicate the timing of plasimd transfection. (x, z) Transfection of plasmids encoding the Lv-mpTALEN(PL6WK)-ELD/Lv-mpTALEN(JR)-KKR pair. (y) Transfection of plasmids encoding the Lv-mpTALEN(PL6LK)-ELD/Lv-mpTALEN(JR)-KKR pair. Heteroplasmy levels following mpTALEN introduction are indicated by different colors. iPSCs were re-cultured after freezing and thawing at the time point indicated by vertical line.

on days 2 and 13 (Figure 6B). However, there were no differences in heteroplasmy levels on day 30.

iPSCs with higher mutation loads may have a reduced proliferation rate,[33] giving them a competitive disadvantage in mixed cell cultures. Therefore, to purify iPSCs with a higher mutation load, after seeding m.3243A(WT)-mpTALEN-applied #54D6-iPSCs on a feeder layer, emerging iPSC colonies were isolated and subcloned (Figure 6C). Most of the 14 selected subclones showed a higher mutation load, up to 97%, than observed when iPSCs applied with m.3243A(WT)-mpTALEN were cultured as a mixture (Figure 6D).

Our improved strategies resulted in obtaining #54D6-iPSCs with varying percentages of m.3243A>G mutant mtDNA. Using either m.3243G(MUT)- or m.3243A(WT)-mpTALEN, we achieved mutation loads ranging from 11% to 97% (Figures 4, 6D, and 7A, upper pie chart figures). Despite these variations in mutation loads, all #54D6-iPSCs maintained their ability to differentiate, as demonstrated by embryoid body (EB)-mediated spontaneous differentiation *in vitro*. Namely, similar to #50V4, #54D1, and #54D7-iPSCs, the mpTALEN-applied #54D6-iPSCs clones spontaneously differentiated into various types of cells that were positive for the endoderm marker Sox17, the ectoderm markers Nestin and tubulin β3 (TUBB3), and the mesoderm marker ACTA2 (α-smooth muscle actin, αSMA) on day 16 (Figures 7A and S15, micrographs). The differences in m.3243A>G mutation loads between iPSC states on day 0 and differentiated cells on day 16 ranged from 0% to 7%, with some cases showing an increase after 16 days (Figures 7A and S15).

Our previous data demonstrated that iPSCs derived from MELAS patients with a higher percentage of m.13513G>A mtDNA (≥84%)

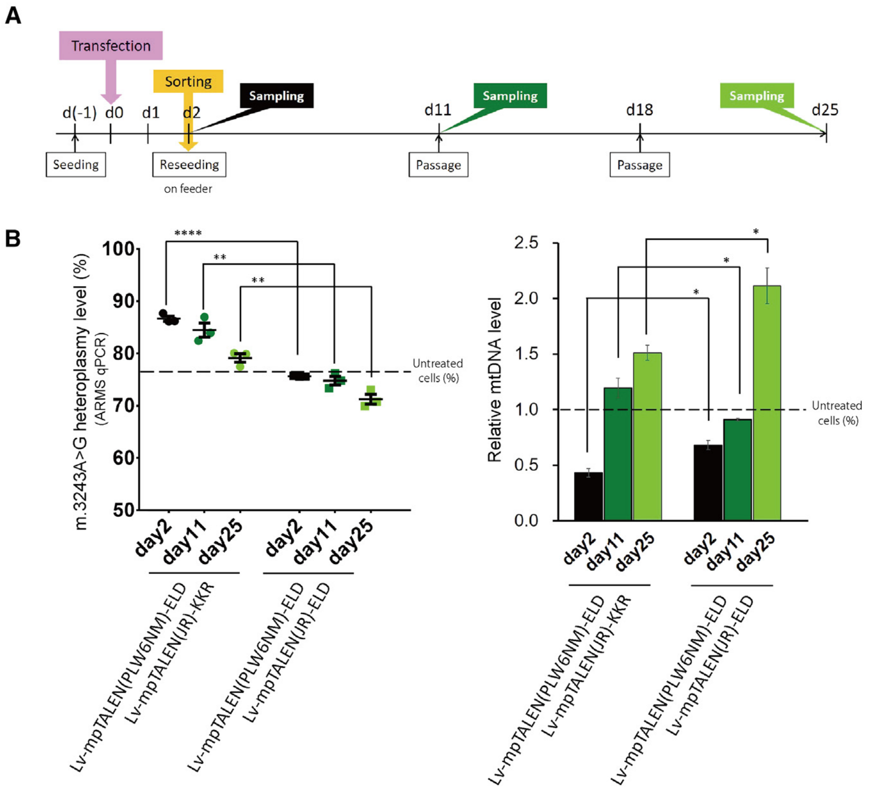

Figure 5. Long-term effects of heterodimeric m.3243A(WT)-mpTALEN expression on mtDNA heteroplasmy and copy numbers in #50V4-iPSCs

(A) Experimental scheme. Two days after transfection, sorted cells were re-cultured on feeder cells for 23 days. (B) Left, heteroplasmy levels at 2, 11, and 25 days after transfection. The dotted line indicates the heteroplasmy level in untreated cells on day 2. Data are expressed as the means ± SEM (n = 3). $^{**}p < 0.01$, $^{****}p < 0.0001$ (Holm-Sidak test). Right, relative mtDNA levels (correlating with mtDNA copy number) at 2, 11, and 25 days after transfection. Data are presented as the ratio of the measured number to that in untreated cells on day 2 and expressed as the means ± SEM (n = 3). The dotted line indicates the relative mtDNA level in untreated cells on day 2. $^{*}p < 0.05$ (Holm-Sidak test).

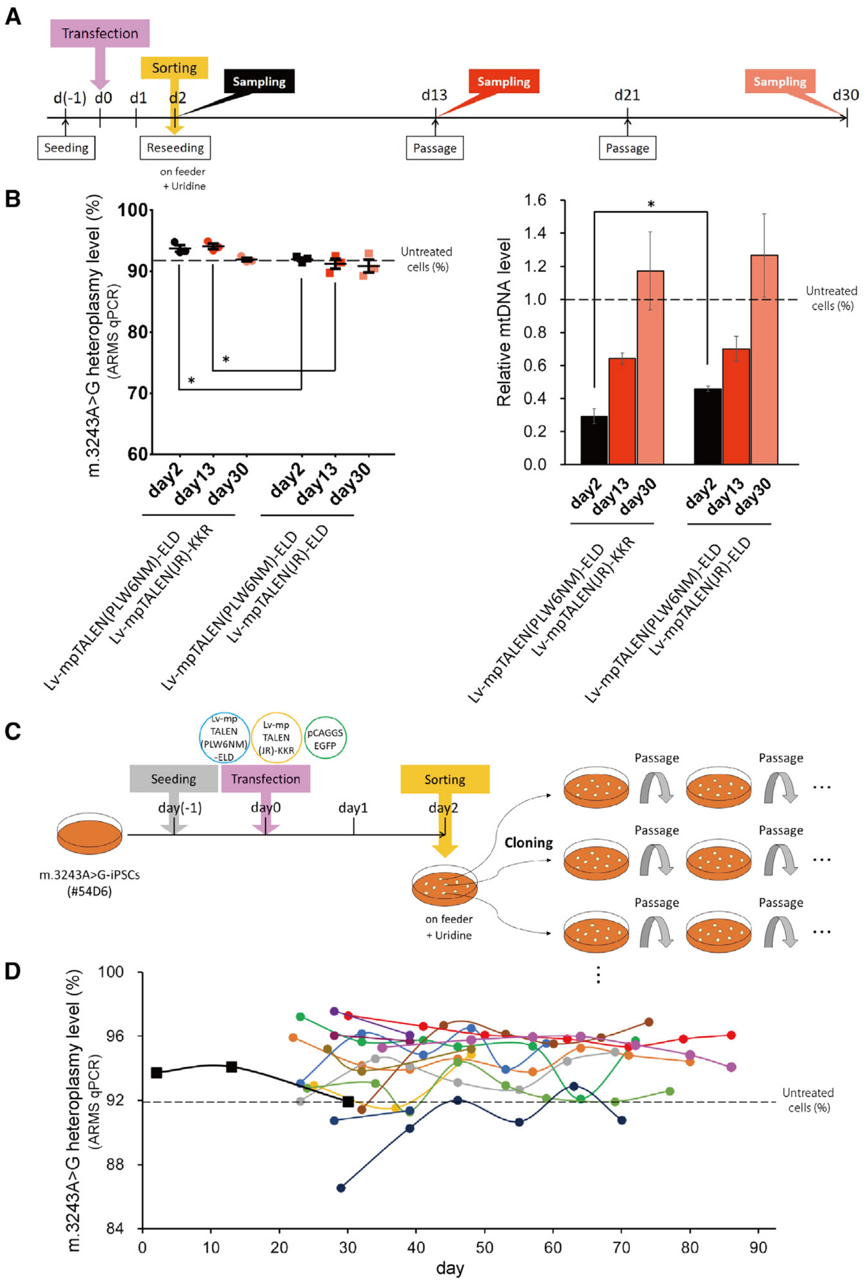

Figure 6. Isolation of m.3243A(WT)-mpTALEN-applied #54D6-iPSC subclones with higher mutation loads

(A) Experimental scheme 1. Two days after transfection, sorted cells were re-cultured with uridine supplementation on feeder cells for 28 days. (B) Left, m.3243A>G heteroplasmy levels at 2, 13, and 30 days after transfection. The dotted line indicates the heteroplasmy level in untreated cells on day 2. Data are expressed as the means ± SEM ($n = 3$). *$p < 0.05$ (Holm-Sidak test). Right, relative mtDNA level (correlating with mtDNA copy number) at 2, 13, and 30 days after transfection. Data are presented as the ratio of the measured number to that in untreated cells on day 2 and expressed as the means ± SEM ($n = 3$). The dotted line indicates the relative mtDNA level in untreated cells on day 2. *$p < 0.05$ (Holm-Sidak test). (C) Experimental scheme 2. Two days after transfection, sorted cells were re-cultured with uridine supplementation on feeder cells. Fourteen iPSC colonies were isolated and separately expanded on feeder cells with uridine supplementation. (D) Long-term fluctuation of m.3243A>G heteroplasmy levels in isolated #54D6-iPSC subclones transfected with the mp[PLW6NM-ELD/JR-KKR] pair. The dotted line indicates the heteroplasmy level in untreated cells on day 2. Black squares indicate m.3243A>G heteroplasmy levels in the mixture of mp[PLW6NM-ELD/JR-KKR]-applied #54D6-iPSCs at 2, 13, and 30 days after transfection, shown in (B).

showed poor differentiation into αSMA-positive cells and TUBB3-positive neuronal cells with extended neurites.[16] However, even #54D6-iPSCs possessing 96% m.3243A>G mutant mtDNA could differentiate into TUBB3-positive neuronal cells and αSMA-positive cells (Figure 7A). Some impact of the mutation load was found though, as qRT-PCR analysis of *αSMA* expression levels in differentiated cells on day 16 revealed that higher m.3243A>G mutation loads in differentiated cells from #54D6-iPSCs moderately correlated with lower *αSMA* expression levels. Nevertheless, even the *αSMA* expression level of the differentiated cells from #54D6-iPSCs with 96% mutation load was still more than 60% of that in differentiated cells derived from the mutation-free A01#15_MyoD33-4 clone[16] (Figure 7B).

DISCUSSION

In this study, we developed and optimized mpTALENs targeting the m.3243A>G mutant mtDNA or the wild-type mtDNA to change heteroplasmy levels in either direction in m.3243A>G-iPSC models. The immediate goal of this bi-directional system is to facilitate the investigation of the relationship between m.3243A>G mutation load and disease phenotypes, because these can vary widely and are not well understood.[34] For the long term, our aim is to develop an effective and safe therapy for patients with m.3243A>G disease. In pursuit of these goals, we made significant progress by developing m.3243G(MUT)-mpTALENs and m.3243A(WT)-mpTALENs with the following components: (1) relatively short TALEs with novel ncRVDs, including LK/WK and NM, that provided the optimal combination of cleavage activity and target specificity (Figures 1, S1, S2, and S3), and (2) obligate heterodimeric FokI domains that increased target specificity and appeared to reduce mtDNA copy number loss due to off-target cleavage (Figures 2, 3, S10, and S13). This bi-directional system of mpTALENs enabled the creation of iPSCs with a wide range of m.3243A>G heteroplasmy.

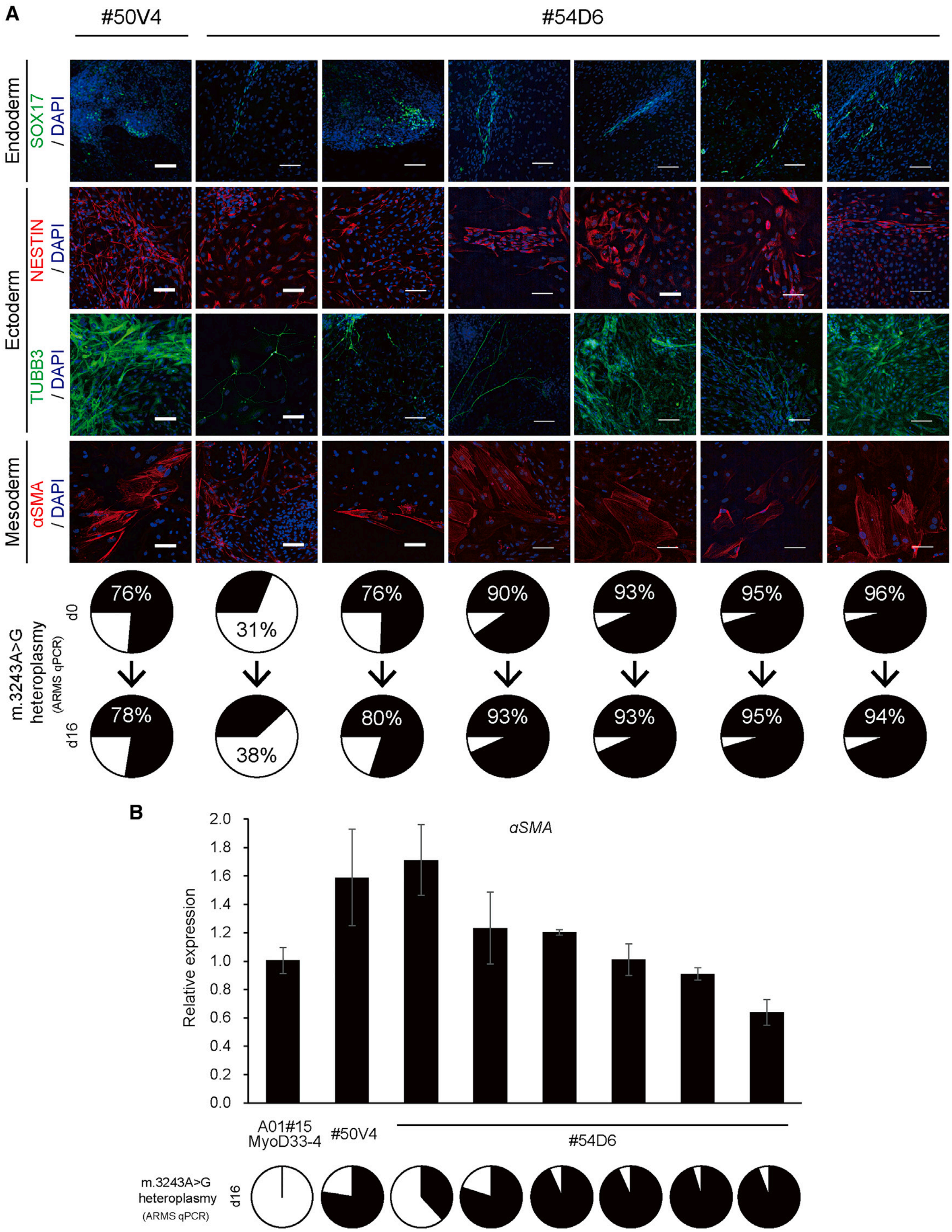

Figure 7. *In vitro* EB-mediated spontaneous differentiation into all three germ layers by mpTALEN-applied m.3243A>G-iPSCs with varying heteroplasmy levels
(A) Differentiated cells from A04#50V4 and mpTALEN-applied B01#54D6-iPSCs were stained on day 16 with anti-SOX17 (endoderm, green), anti-NESTIN (ectoderm, red), anti-TUBB3 (ectoderm, green), and anti-αSMA (mesoderm, red) antibodies. Nuclei were stained with DAPI. Scale bars, 100 μm. Pie charts at the bottom show m.3243A>G heteroplasmy in undifferentiated iPSCs on day 0 and in differentiated cells on day 16, with black and white regions representing percentages of mutant and wild-type mtDNA, respectively. (B) Quantitative RT-PCR analysis for *αSMA* in differentiated cells from A01#15_MyoD33-4 (#33-4),[16] #50V4, and mpTALEN-applied #54D6 on day 16. The graph represents relative gene expression compared with that measured for #33-4 (n = 3; error bars, SD). *ACTB* was used as an internal control. Pie charts at the bottom show m.3243A>G heteroplasmy levels in differentiated cells on day 16.

For stably selecting these clones, including those that may have a competitive disadvantage, we employed additional techniques such as uridine supplementation and subcloning (Figure 6). This approach resulted in a palette of viable iPSCs, all retaining their ability to differentiate into three germ layers, with mutation loads varying from 31% to 96% (Figure 7). We believe that with this bi-directional system and the use of iPSCs, we have created a valuable tool for studying m.3243A>G pathology across different genetic patient backgrounds. A key advantage of our bi-directional mpTALEN system is that it enables the use of isogenic cells to analyze phenotypes that are solely dependent on heteroplasmy levels. This approach may also be extended to other heteroplasmic models, such as cybrid cells.

The DNA binding domain of TALEN consists of modules of 34 amino acids, each with divergent 12th and 13th amino acids forming an RVD. Traditionally, four conventional RVDs (NI, HD, NN, and NG)—all found in nature—have been used, but NN is not very specific as it binds both adenine and guanine.[35,36] Previous research has demonstrated that alternatives to NN, such as NK and NH, can improve specificity for guanine.[37,38] Additionally, other ncRVDs with novel intrinsic targeting specificity features have been identified and characterized.[19,39,40] For example, in TALENs targeting nuclear genes, ncRVDs such as GN for adenine; VG and LP for thymine; SD, NM, and QD for cytosine; and GR, HN, and RH for guanine have been shown to improve target specificity.[19,39,41] This study is the first to use ncRVDs in TALENs to target point-mutated mtDNA. Unexpectedly, in m.3243G(MUT)-mpTALEN, LK and WK were more effective than the well-known NK, enhancing the recognition of m.3243G over m.3243A. In designing m.3243A(WT)-mpTALEN to recognize m.3243A over m.3243G, HY was initially a leading candidate for the ncRVD.[19] However, contrary to expectations, NM exhibited slightly higher specificity for wild-type sequence compared with HY and was therefore selected. Our results provide evidence that using ncRVDs can help optimize TALEN design for targeting point-mutated mtDNA. As a next step, we will examine whether the same ncRVD can be used for mtDNA with other A/G mutations that cause mitochondrial diseases. In addition, as our analysis of possible ncRVDs has not been exhaustive, we also intend to investigate more ncRVD possibilities for targeting point-mutated mtDNA.

Several mito-nucleases, including our mpTALEN, have been reported to alter the percentage of mutant mtDNA,[42] but designing them appropriately is not easy. The most widely used programmable nuclease system is CRISPR-Cas, due to its ease of design and production. However, when targeting mtDNA (mito-CRISPR), not only the Cas protein but also the guide RNA (gRNA) needs to be delivered into mitochondria, which is hampered by their double membrane. Stem-loop secondary RNA structures may facilitate RNA transport into mitochondria, and combining motifs that confer such structures with gRNAs can promote their import as well.[43] However, the technique is not yet well established, and although introducing mito-CRISPR has been shown to induce a decrease in mtDNA copy number, it has, to the best of our knowledge, not yet succeeded in modifying the mutation load in heteroplasmic cells.[44] Additionally, designing a mito-CRISPR system that specifically targets a mutant mtDNA sequence requires selecting a CRISPR-Cas system with an appropriate PAM and screening for gRNAs that specifically recognize the mutant mtDNA.[45] We are hopeful that, in comparison, our mpTALEN system using ncRVDs may be more easily generalized, making it a convenient and valuable tool for modifying heteroplasmy levels in both clinical and basic research applications.

Our study observed substantial decreases in mtDNA copy numbers when comparing mpTALEN-treated cells with untreated controls—which were non-transfected and non-sorted—at 2 days post-infection (Figures 3C, 3E, 5B, 6B, and S13C). This reduction was possibly also caused by factors other than FokI cleavage activity, such as cell sorting using forward scatter/side scatter-gating to avoid doublet contamination. Since the current experimental protocol has low efficiency in introducing mpTALEN into iPSCs, the cell sorting is essential; hopefully, a more efficient method for mpTALEN transfection will be developed, making cell sorting unnecessary and preventing it from potentially reducing the average copy number. Better negative controls were formed by using mpTALEN pairs in which each of the FokI domains was of the ELD type, as cell treatments were identical but cleavage-competent homodimers are believed to be absent in such condition.[46] Transfection with these ELD/ELD controls also led to reduced mtDNA copy numbers compared with untreated controls, but this reduction was considerably smaller than those observed with cleavage-competent mpTALENs, whether using conventional or ELD/KKR obligate heterodimeric FokI domains (Figures 3C, 3E, 5B, 6B, and S13C). The reduction in mtDNA copy numbers relative to ELD/ELD controls was consistently less dramatic after treatment with obligate heterodimeric ELD/KKR mpTALEN pairs than with pairs including similar TALEs but conventional FokI domains (Figures 3C, 3E, and S13C). This, in combination with the effects seen on the m.3243A>G heteroplasmy levels (Figures 3B, 3D, and S13B), indicates that using the ELD/KKR system reduced off-target mtDNA cleavage and enhanced the relative frequency of target-specific mtDNA cleavage. However, our developed m.3243G(MUT)-mpTALENs seem to not have entirely eliminated off-target mtDNA cleavage (Figure S12). Further refinements may be necessary to minimize off-target mtDNA cleavage as close to zero as possible.

Two types of mito-nucleases targeting the m.3243A>G mutation have been previously reported: mitoARCUS and mitoTALEN.[10,17] MitoARCUS was demonstrated to effectively reduce the m.3243A>G mutation load while no detrimental effect of mtDNA copy number depletion was observed in cybrid cells.[10] From target cleavage efficiency and the proven absence of off-target cleavage perspectives, the mitoARCUS system[10] appears superior to our mpTALEN system in reducing the m.3243A>G mutation load. However, for a proper comparison, the systems should be studied side-by-side. Furthermore, even if the developed mitoARCUS system would be superior in reducing m.3243A>G mutation load, this might not be due to an intrinsic superiority of the mitoARCUS technology but possibly resulted from the respective researchers using more efficient rounds of optimization through computer predictions and

functional selections[10]; potentially, such approach could also benefit the mpTALEN system. Only the future can tell which technique will be favorable in the clinic, which will also depend on other factors such as immunogenicity. From a medical perspective, it is only positive that different routes of genetic engineering show promise for treating patients with mitochondrial disease. From a research perspective, current disadvantages of the mitoARCUS system compared with the mpTALEN system are that, arguably, its versatility in binding any possible target sequence remains to be proven and that it cannot be developed in a normal laboratory.

An advantage of our here-described mpTALEN system compared with a previously reported A3243G-mitoTALEN[17] is the smaller size. Recombinant adeno-associated virus (AAV) is commonly used to deliver mito-nucleases into animal models,[47–49] but has a packaging size limitation. The previously reported A3243G-mitoTALEN was quite long with arrays of 16 modules in the TALE domains,[17] while our developed m.3243G(MUT)-mpTALEN monomers have only 11 or 12 modules, making their DNA better suited for packaging within an AAV capsid. The ability of our mpTALENs to efficiently modify mtDNA mutation load despite having relatively few TALE modules may be due to the use of highly active Platinum TALEN and ncRVDs. A recent study demonstrated that introducing mitoTALEN into the central nervous system using an AAV vector could reduce the mutation load and recover tRNA alanine levels in a mouse model,[50] paving the way for clinical application in patients with mitochondrial diseases, including MELAS.

In our previous report, G13513A-mpTALEN induced a large decrease in mtDNA copy number, which we could suppress by modifying codons in the expression vectors to generate lower expression levels.[16] In the present study, we did not pursue this route, because the reductions in mtDNA copy number were more moderate and easily recovered. However, to work toward clinical applications, we may explore the potential advantages of changing m.3243G(MUT)-mpTALEN expression levels for further optimizing/balancing cleavage activity and target specificity. Introducing mpTALENs in the form of mRNA is one of the effective ways to control their expression level,[10] but also other delivery methods such as various virus-like particles (VLPs) or lipid nanoparticles (LNPs) can be considered for this purpose.[51]

For future experiments, in addition to addressing potential side effects, it remains important to recognize that there is a threshold of mtDNA mutation loads that causes pathological mitochondrial dysfunction. The primary goal of mpTALEN-based therapeutic treatment is to reduce the mutation load below this threshold. We will use *in vivo* models to determine the most appropriate delivery technologies and other variables necessary to achieve this outcome.

Our previous study on another mitochondrial mutation demonstrated that spontaneously differentiated cells from m.13513G>A-iPSCs with higher mutation loads (>75%) showed drastic decreases in *αSMA* expression and limited neurite extension of TUBB3-positive cells.[16] In contrast, in the present study, αSMA-positive cells and TUBB3-positive neurites were observed in relatively normal abundancies upon EB-mediated differentiation of iPSCs even if having a high m.3243A>G mutation load (∼95%). Nevertheless, also in our study, the m.3243A>G mutation load may have had some impact on the differentiation characteristics of m.3243A>G-iPSCs because qPCR data show that the *αSMA* expression decreased with increasing mutation load (Figure 7B). In a previous study on spinal organoids derived from m.3243A>G-iPSCs, it was found that an approximately 80% mutation load was associated with neurodevelopmental defects,[52] and in another study high proportions of m.3243A>G mtDNA (>90%) were associated with neuronal cell death and impairment in cardiac lineage commitment.[53] Taken together, the data indicate that iPSC differentiation is more sensitive to m.13513G>A mutation load than to m.3243A>G mutation load, but that depending on the system, also high m.3243A>G mutation loads can lead to differentiation defects. In the near future, we plan to differentiate these heteroplasmic iPSCs into relevant somatic cell types and analyze their cellular phenotypes.

In summary, our study describes the successful development of a bidirectional mpTALEN system capable of manipulating m.3243A>G heteroplasmy levels in patient-derived iPSCs while retaining their differentiation potential. By enabling not only a reduction but also an increase of mutation loads—which, to the best of our knowledge, has not been done before—this system provides a versatile and powerful tool for studying mitochondrial disease pathology across different genetic patient backgrounds. Our results underscore the importance of advanced techniques, such as the use of non-conventional RVDs and obligate heterodimeric FokI domains, for enhancing cleavage activity and specificity while reducing off-target effects. Future work should include a focus on translating these findings into clinical applications, promoting the safety and efficacy of mpTALEN-based therapies for m.3243A>G and other mitochondrial disorders.

MATERIALS AND METHODS

Human iPSC generation and culture

We obtained skin biopsy specimens from patients with mitochondrial disease caused by the m.3243A>G mutation. This study was approved by the Ethics Committee of Fujita Health University and the NCNP Institutional Review Board. This study was stringently conducted in accordance with the ethical principles of the Declaration of Helsinki. We received written informed consent with permission to study patient-derived iPSCs. We expanded the patients' fibroblasts (named B01 and A04) in DMEM containing 10% FBS and 1% antibiotic-antimycotic solution (FUJIFILM Wako Pure Chemical Corp., Osaka, Japan); 5×10^5 fibroblasts were transduced with six reprogramming factors (*SOX2*, *KLF4*, *OCT3/4*, *L-MYC*, *LIN28*, *p53*-shRNA) by episomal vectors (pCXLE-hOCT3/4-shp53-F, Addgene #27077; pCXLE-hSK, #27078; and pCXLE-hUL, #27080), donated by Shinya Yamanaka[54] using Nucleofector 2b (Lonza) with an Amaxa Human Dermal Fibroblast Nucleofector Kit (Lonza). Seven days after transduction, fibroblasts were plated on a mitomycin C-treated SNL feeder

layer. The next day, the medium was changed to Primate ES Cell Medium (ReproCELL, Yokohama, Japan) supplemented with basic fibroblast growth factor (bFGF) (FUJIFILM Wako). After a few weeks, ESC-like colonies were mechanically dissociated and transferred onto new plates. iPSCs were harvested by treatment with CTK solution consisting of 0.1 mg/mL collagenase IV (Gibco/Thermo Fisher Scientific), 0.25% trypsin (Gibco), 0.1 mM $CaCl_2$ (FUJIFILM Wako), and 20% KSR (Gibco).

RNA isolation and RT-PCR

Total RNA was purified using an RNeasy Plus Mini Kit (Qiagen). One microgram of total RNA was used for reverse transcription reaction with ReverTra Ace qPCR RT Kit (Toyobo, Osaka, Japan), according to the manufacturer's protocol. PCR was performed with TaKaRa Ex Premier DNA polymerase (Takara Bio, Shiga, Japan). PCR cycle conditions consisted of 94°C for 1 min, 30 cycles of 98°C for 10 s, 60°C for 15 s, and 68°C for 30 s, and final cooling to 4°C. Primer sequences are shown in Table S1. PCR products were run in a 1% agarose gel and stained with ethidium bromide.

Karyotyping analysis

Standard G-band chromosome analysis was performed by OVUS Co., Ltd. (Aichi, Japan).

EB-mediated spontaneous differentiation of iPSCs

Spherical clusters of iPSCs re-suspended in DMEM/F12 (Gibco) containing 20% KSR (Gibco), 2 mM GlutaMAX (Gibco), 100 μM non-essential amino acid (NEAA) (Gibco), 100 μM 2-mercaptoethanol (Nacalai tesque, Kyoto, Japan), and a 0.5% antibiotic-antimycotic solution (FUJIFILM Wako) were transferred to Petri dishes. After an 8-day floating culture, spontaneously formed EBs were transferred to Matrigel matrix basement membrane growth factor reduced (Corning)-coated plates and incubated for another 8 days.

Immunocytochemical analysis

Cells were fixed with 4% paraformaldehyde/PBS for 30 min and incubated in PBS containing 0.2% Triton X-100 for 10 min. After blocking with 2% BSA/PBS for 1 h, cells were incubated with primary antibodies diluted with blocking buffer and then washed with PBS. Finally, the cells were incubated with secondary antibodies, washed with PBS, and mounted using ProLong Diamond Antifade Mountant with DAPI (Molecular Probes). Immunoreactive cells were visualized using a confocal laser scanning microscope (Carl Zeiss, LSM710 and LSM980) or Biorevo BZ-9000 fluorescence microscope (Keyence).

DNA isolation

Genomic DNA including mitochondrial DNA was isolated from fibroblasts and iPSCs using a NucleoSpin Tissue XS (Macherey-Nagel) according to its manufacturer's protocol.

Analysis of mtDNA mutation by DNA sequencing

A mitochondrial genome fragment including the m.3243 position was amplified using PrimeSTAR GXL DNA polymerase (Takara Bio) with PCR primers (Mito-1-2F and Mito-1-2R, described in Table S1). The PCR amplicon was purified using Wizard SV Gel and PCR Clean-UP System (Promega) and sequenced by the Sanger method with mt3150-F primer (Table S1) through a sequencing service (FASMAC Co., Ltd., Kanagawa, Japan).

Analysis of mtDNA heteroplasmy by PCR-RFLP

The DNA was amplified using TaKaRa Ex Taq or Tks Gflex DNA Polymerase Low DNA (Takara Bio) with mt3150-F and mt3294-R primers (described in Table S1). PCR conditions were 94°C for 1 min, followed by 35 cycles of 94°C for 30 s, 57°C for 30 s, 72°C for 30 s (TaKaRa Ex Taq), or 94°C for 1 min, followed by 40 cycles of 98°C for 10 s, 57°C for 15 s, and 68°C for 10 s (Tks Gflex). In the presence of m.3243A>G mutation, the 145-base pair (bp) PCR product was cleaved by HaeIII (NEB) to two fragments of 95 and 50 bp. The HaeIII-digested PCR products were separated on a 3% agarose gel and stained with ethidium bromide. ImageJ software for Windows was used to analyze the ratio of the signal intensity.

Analysis of mtDNA heteroplasmy by ARMS-qPCR

Quantification of m.3243A>G mtDNA heteroplasmy was also performed by ARMS-qPCR using a QuantStudio 7 (Applied Biosystems) as previously described,[28] with partial modifications. The 10-μL reaction mixture contained 0.15 ng of template DNA, GeneAce SYBR qPCR Mix α with Low ROX (Nippon Gene, Tokyo, Japan), and primers ARMS-A3243G(WT)_F1 and ARMS-A3243G_R1 for wild-type mtDNA or ARMS-A3243G(MUT)_F1 and ARMS-A3243G_R1 for mutant mtDNA (Table S1). qPCR was performed in triplicate for each DNA sample. The PCR cycle conditions consisted of 95°C for 10 min, and 40 cycles of 95°C for 15 s and 63°C for 30 s. The copy number of WT or MUT mtDNA was calculated based on a standard curve using absolute standard plasmid including WT or MUT amplicon. The m.3243A>G heteroplasmy level was calculated as (MUT mtDNA copy number)/(WT mtDNA copy number + MUT mtDNA copy number) × 100.

Measurement of mitochondrial DNA copy number

mtDNA copy number of iPSCs was determined using an ABI PRISM 7900HT (Applied Biosystems) as described previously,[15] with partial modification. The 20-μL reaction mixture contained 0.9 ng of template DNA, GeneAce SYBR qPCR Mix α (Nippon Gene), with primers MT-CYB-F and MT-CYB-R for mtDNA, or FBXO15-F and FBXO15-R for nuclear DNA (Table S1). qPCR was performed in triplicate. The copy number of targeted DNA was calculated based on a standard curve using absolute standard plasmid including MT-CYB and FBXO15 amplicons. mtDNA copy number was calculated as (MT-CYB copy number)/2 × (FBXO15 copy number).

Construction of TALEN expression plasmids

We explored m.3243G(MUT)- and m.3243A(WT)-pTALEN design candidates using TAL Effector Nucleotide Targeter 2.0[55] (https://tale-nt.cac.cornell.edu/node/add/talen.). The mtDNA sequences from positions 3197 to 3288 mtDNA were used as target sequences. The search criteria were set with spacer lengths ranging from 13 to 22 and repeat array lengths ranging from 10 to 19. Additionally,

"NN" was selected as the G substitute, and the Filter Options were adjusted to "Show all TALEN pairs (include redundant TALEN)," while all other conditions remained at their default settings. From the resulting design candidates, several designs were selected where the RVDs of either the left-TALEN or right-TALEN recognize the nucleotide at m.3243.

TALEN expression plasmids were constructed using the Platinum Gate TALEN kit (#1000000043, Addgene). We adapted the Golden Gate method for four-module assembly and final TALEN vector assembly using four-module ligand plasmids as described in the manufacturer's protocol for the Platinum Gate TALEN kit. Restriction enzyme BsaI-HFv2 (NEB) was used in the first step of Golden Gate cloning.

pTALE-DNA binding module vectors containing ncRVDs, p2LK, p2WK, p2FK, p2NK, p2NM, or p2HY were generated by modifying a p2NI vector. DNA fragment including the ncRVD, synthesized by Azenta (Tokyo, Japan), was inserted into a p2NI vector, digested with HindIII and AgeI, using the In-Fusion HD cloning kit (Clontech Laboratories).

The destination vector to express a platinum TALEN (pTALEN) monomer possesses a +136/+63 scaffold under the control of the CAG promoter. The destination vector used to express an mpTALEN monomer additionally encodes a mitochondrial targeting sequence (MTS) of ATP5B and a V5-tag for adding to the TALEN N terminus as previously described.[16]

The destination vectors of mpTALEN scaffolds with the heterodimeric FokI (ELD/KKR) (Figure 2)[21] were generated by modifying the destination vectors of mpTALENs. A DNA fragment including ELD mutations, synthesized by Azenta (Tokyo, Japan), was inserted into an mpTALEN destination vector, which was digested with BamHI and BstXI, using the In-Fusion HD cloning kit. Three DNA fragments including KKR mutations, amplified by PCR with primer pairs, BamHI_Fok1_fwd and K1-Fok1_rev, K1-Fok2_fwd and R2K3-Fok2_rev, R2K3-Fok3_fwd and SexAI_Fok3_rev (described in Table S1), were inserted into an mpTALEN destination vector, which was digested with BamHI and SexAI, using the In-Fusion HD cloning kit.

SSA assay using HEK293T cells

TALEN activity may be evaluated using an SSA assay as previously described.[24] The TALEN target sequence including m.3243A or m.3243G was amplified using XmaI-A3243_F and XmaI-A3243AWT_R3 or XmaI-A3243G_R3 primers (Table S1) by PCR and inserted into the Xma I site between the bisected luciferase elements of the pGL4-SSA reporter plasmid.[56] HEK293T cells were transfected with three types of plasmids, comprising TALEN plasmids, a reporter plasmid, and a reference plasmid for dual-luciferase assay (pRL-CMV; Promega), by using Lipofectamine LTX (Invitrogen/ThermoFisher Scientific). Dual-luciferase assays were performed at 24 h after transfection using a Dual-Glo luciferase assay system (Promega) in an ARVO X5 luminometer (PerkinElmer) or VICTOR Nivo Multimode Microplate reader (PerkinElmer). SSA activity (Luc/RLuc) was calculated as firefly luminescence/*Renilla* luminescence. The m.3243G(MUT) target specificity was calculated as [Luc/Rluc(MUT, pTALEN+) – Luc/RLuc(MUT, NC)]/[Luc/Rluc(WT, pTALEN+) – Luc/RLuc(WT, NC)]. The m.3243A(WT) target specificity was calculated by the inverse formula.

Cells: Transfection and sorting

HEK293T and HeLa cells were transfected using Lipofectamine 3000 reagent (Invitrogen) according to the manufacturer's protocol.

Patient-derived iPSCs with m.3243A>G mutation were cultured under feeder-free conditions in accordance with a previous report[57] for the transduction of the mpTALEN plasmids. After removing feeder cells, iPSCs were dissociated into single cells by 0.5× TrypLE Select (Gibco) for about 2 min at 37°C. Single iPSCs were reseeded on dishes coated with iMatrix-511 (Matrixome, Osaka, Japan) and cultured using StemFit AK02N medium (Ajinomoto, Tokyo, Japan) with 10 μM Y-27632 (Nacalai tesque). One day later, pCAGGS-EGFP[15] and mpTALEN plasmids (1.67 μg each) were introduced into iPSCs using Lipofectamine 3000 reagent (Invitrogen) according to the manufacturer's protocol. Two days later, the cells were harvested and sorted using a Moflo Astrios (Beckman Coulter) with Summit acquisition software (Beckman Coulter). A sorting gate was established based on forward and side scatter as well as the level of EGFP expression, after the exclusion of dead cells and debris, which were stained with propidium iodide (PI) solution (Dojindo, Kumamoto, Japan). EGFP-positive and PI-negative cells were directly sorted into StemFit AK02N medium in feeder-free conditions or Primate ES Cell Medium on feeders, with 10 μM Y-27632. Fifty micrograms per milliliter uridine (Sigma) was supplemented into the culture medium to maintain m.3243A>G-iPSCs with a higher mutation load. "Untreated cells" are cells harvested 3 days after seeding without transfection or cell sorting.

Western blotting

Transfected HEK293T cells were pelleted and lysed with a radioimmunoprecipitation assay (RIPA) buffer containing a protease inhibitor cocktail (Roche). After sonication and centrifugation, the protein concentration of the supernatant was determined using a Pierce bicinchoninic acid (BCA) protein assay kit (Thermo Scientific). Lysed protein (13 μg) with sample buffer (EzApply; ATTO, Tokyo, Japan) was denatured at 95°C for 5 min and separated by SDS-PAGE in 4%–15% gels (Bio-Rad). Proteins were transferred to Immobilon-FL membranes (Millipore). The membrane was incubated with Revert 700 Total Protein Stain Solution (LI-COR) for 5 min. After washing the membrane with Revert 700 Wash Solution (LI-COR), a total protein signal image was captured using an Odyssey CLx Infrared Imaging System (LI-COR) with the infrared (IR) 680 channel. The membrane was blocked with Intercept Blocking Buffer TBS (LI-COR) and then incubated with primary antibodies at room temperature for 2 h. After washing with TBS-T, the membrane was further incubated with IRDye 800CW Goat anti-Mouse immunoglobulin (Ig)G (LI-COR) at room temperature for 1 h. The membrane

was washed with TBS-T, and protein signals were detected using an Odyssey CLx Infrared Imaging System with the IR800 channel.

Antibodies

The following primary antibodies were used: mouse anti-V5-tag (ICC, 1:300; WB, 1:1,000; R960-25, Invitrogen), rabbit anti-TOM20 (1:100; sc-11415, Santa Cruz Biotechnology), mouse anti-SSEA-4 (1:100; MAB4304, Millipore), goat anti-NANOG (1:20; AF1997, R&D Systems), rabbit anti-OCT4 (1:200; ab19857, Abcam), mouse anti-αSMA (1:200; M0851, Dako), goat anti-SOX17 (1:200; AF1924, R&D Systems), mouse anti-NESTIN (1:200; MAB5326, Millipore), and rabbit anti-TUBB3 (1:1,500; PRB-435P, BioLegend). Alexa Fluor 488 (A11055 or A11034, Molecular Probes)-, Alexa Fluor 594 (A11005, Molecular Probes; ab150132, Abcam)-, and Alexa Fluor 647 (ab150075, Abcam)-conjugated secondary antibodies were used for immunofluorescence studies.

Statistical analysis

Statistical significance of differences between two groups was determined using the Holm-Sidak method. Comparisons among three or four groups were performed using one-way or two-way ANOVA, followed by Tukey's test (GraphPad Prism; GraphPad). Differences were considered significant at a p value < 0.05.

DATA AND CODE AVAILABILITY

All data generated or analyzed during this study are included in this published article and its supplemental information files.

ACKNOWLEDGMENTS

We would like to express our sincere gratitude to the patients and their families for their participation in our research. We are also grateful to all our coworkers, especially Dr. Kyoko Ibaraki, Sayaka Matsumoto, Dr. Seika Hashimoto, Takafumi Kawamura, Takaumi Baba (Fujita Health University), and to Dr. Kentaro Tsukamoto and Chikako Hibiya (Fujita Health University) for assistance at the Open Facility Center, and to Dr. Johannes M. Dijkstra for proofreading this manuscript. The authors also wish to express their thanks to Dr. Takashi Yamamoto for supplying the Platinum Gate TALEN kit and pGL4-SSA plasmid. This study was supported by the Takeda Science Foundation (to N.Y.), the Fujita Health University Research Fund (to N.Y. and R.H.), and the Japan Society for the Promotion of Science (JSPS) KAKENHI (JP21K06848 to N.Y. and JP24K11010 to R.H.), and the support project of research seeds transfer to industries of Japanese Society of Neurology (to R.H.).

AUTHOR CONTRIBUTIONS

N.Y. and R.H. conceived the project. N.Y. designed the experiments. Y.-i.G. and R.H. provided the study material. N.Y. and R.H. performed the experiments. N.Y. analyzed the data. N.Y. wrote original draft. All of the authors read and approved the final manuscript.

DECLARATION OF INTERESTS

Fujita Academy has submitted a patent application based in part on the data reported in this paper, with N.Y. and R.H. as inventors.

SUPPLEMENTAL INFORMATION

Supplemental information can be found online at https://doi.org/10.1016/j.omtn.2025.102521.

REFERENCES

1. Stewart, J.B., and Chinnery, P.F. (2015). The dynamics of mitochondrial DNA heteroplasmy: implications for human health and disease. Nat. Rev. Genet. *16*, 530–542.
2. Goto, Y., Nonaka, I., and Horai, S. (1990). A mutation in the tRNA(Leu)(UUR) gene associated with the MELAS subgroup of mitochondrial encephalomyopathies. Nature *348*, 651–653.
3. Li, D., Liang, C., Zhang, T., Marley, J.L., Zou, W., Lian, M., and Ji, D. (2022). Pathogenic mitochondrial DNA 3243A>G mutation: From genetics to phenotype. Front. Genet. *13*, 951185.
4. Ryytty, S., and Hamalainen, R.H. (2023). The Mitochondrial m.3243A>G Mutation on the Dish, Lessons from In Vitro Models. Int. J. Mol. Sci. *24*, 13478.
5. Silva-Pinheiro, P., and Minczuk, M. (2022). The potential of mitochondrial genome engineering. Nat. Rev. Genet. *23*, 199–214.
6. Bacman, S.R., Williams, S.L., Pinto, M., Peralta, S., and Moraes, C.T. (2013). Specific elimination of mutant mitochondrial genomes in patient-derived cells by mitoTALENs. Nat. Med. *19*, 1111–1113.
7. Gammage, P.A., Rorbach, J., Vincent, A.I., Rebar, E.J., and Minczuk, M. (2014). Mitochondrially targeted ZFNs for selective degradation of pathogenic mitochondrial genomes bearing large-scale deletions or point mutations. EMBO Mol. Med. *6*, 458–466.
8. Hashimoto, M., Bacman, S.R., Peralta, S., Falk, M.J., Chomyn, A., Chan, D.C., Williams, S.L., and Moraes, C.T. (2015). MitoTALEN: A General Approach to Reduce Mutant mtDNA Loads and Restore Oxidative Phosphorylation Function in Mitochondrial Diseases. Mol. Ther. *23*, 1592–1599.
9. Reddy, P., Ocampo, A., Suzuki, K., Luo, J., Bacman, S.R., Williams, S.L., Sugawara, A., Okamura, D., Tsunekawa, Y., Wu, J., et al. (2015). Selective elimination of mitochondrial mutations in the germline by genome editing. Cell *161*, 459–469.
10. Shoop, W.K., Lape, J., Trum, M., Powell, A., Sevigny, E., Mischler, A., Bacman, S.R., Fontanesi, F., Smith, J., Jantz, D., et al. (2023). Efficient elimination of MELAS-associated m.3243G mutant mitochondrial DNA by an engineered mitoARCUS nuclease. Nat. Metab. *5*, 2169–2183.
11. Mok, B.Y., de Moraes, M.H., Zeng, J., Bosch, D.E., Kotrys, A.V., Raguram, A., Hsu, F., Radey, M.C., Peterson, S.B., Mootha, V.K., et al. (2020). A bacterial cytidine deaminase toxin enables CRISPR-free mitochondrial base editing. Nature *583*, 631–637.
12. Yi, Z., Zhang, X., Tang, W., Yu, Y., Wei, X., Zhang, X., and Wei, W. (2024). Strand-selective base editing of human mitochondrial DNA using mitoBEs. Nat. Biotechnol. *42*, 498–509.
13. Cho, S.I., Lee, S., Mok, Y.G., Lim, K., Lee, J., Lee, J.M., Chung, E., and Kim, J.S. (2022). Targeted A-to-G base editing in human mitochondrial DNA with programmable deaminases. Cell *185*, 1764–1776.e12.
14. Lei, Z., Meng, H., Liu, L., Zhao, H., Rao, X., Yan, Y., Wu, H., Liu, M., He, A., and Yi, C. (2022). Mitochondrial base editor induces substantial nuclear off-target mutations. Nature *606*, 804–811.
15. Yahata, N., Matsumoto, Y., Omi, M., Yamamoto, N., and Hata, R. (2017). TALEN-mediated shift of mitochondrial DNA heteroplasmy in MELAS-iPSCs with m.13513G>A mutation. Sci. Rep. *7*, 15557.
16. Yahata, N., Boda, H., and Hata, R. (2021). Elimination of Mutant mtDNA by an Optimized mpTALEN Restores Differentiation Capacities of Heteroplasmic MELAS-iPSCs. Mol. Ther. Methods Clin. Dev. *20*, 54–68.
17. Yang, Y., Wu, H., Kang, X., Liang, Y., Lan, T., Li, T., Tan, T., Peng, J., Zhang, Q., An, G., et al. (2018). Targeted elimination of mutant mitochondrial DNA in MELAS-iPSCs by mitoTALENs. Protein Cell *9*, 283–297.
18. Chen, X., Chen, M., Zhu, Y., Sun, H., Wang, Y., Xie, Y., Ji, L., Wang, C., Hu, Z., Guo, X., et al. (2023). Correction of a homoplasmic mitochondrial tRNA mutation in patient-derived iPSCs via a mitochondrial base editor. Commun. Biol. *6*, 1116.
19. Juillerat, A., Pessereau, C., Dubois, G., Guyot, V., Maréchal, A., Valton, J., Daboussi, F., Poirot, L., Duclert, A., and Duchateau, P. (2015). Optimized tuning of TALEN specificity using non-conventional RVDs. Sci. Rep. *5*, 8150.
20. Cade, L., Reyon, D., Hwang, W.Y., Tsai, S.Q., Patel, S., Khayter, C., Joung, J.K., Sander, J.D., Peterson, R.T., and Yeh, J.R.J. (2012). Highly efficient generation of heritable zebrafish gene mutations using homo- and heterodimeric TALENs. Nucleic Acids Res. *40*, 8001–8010.
21. Nakajima, K., and Yaoita, Y. (2013). Comparison of TALEN scaffolds in Xenopus tropicalis. Biol. Open *2*, 1364–1370.

22. Naitou, A., Kato, Y., Nakanishi, T., Matsuura, T., and Watanabe, H. (2015). Heterodimeric TALENs induce targeted heritable mutations in the crustacean Daphnia magna. Biol. Open *4*, 364–369.

23. Sakuma, T., Ochiai, H., Kaneko, T., Mashimo, T., Tokumasu, D., Sakane, Y., Suzuki, K.i., Miyamoto, T., Sakamoto, N., Matsuura, S., and Yamamoto, T. (2013). Repeating pattern of non-RVD variations in DNA-binding modules enhances TALEN activity. Sci. Rep. *3*, 3379.

24. Sakuma, T., Hosoi, S., Woltjen, K., Suzuki, K.I., Kashiwagi, K., Wada, H., Ochiai, H., Miyamoto, T., Kawai, N., Sasakura, Y., et al. (2013). Efficient TALEN construction and evaluation methods for human cell and animal applications. Genes Cells *18*, 315–326.

25. Miller, J.C., Tan, S., Qiao, G., Barlow, K.A., Wang, J., Xia, D.F., Meng, X., Paschon, D.E., Leung, E., Hinkley, S.J., et al. (2011). A TALE nuclease architecture for efficient genome editing. Nat. Biotechnol. *29*, 143–148.

26. Mak, A.N.S., Bradley, P., Cernadas, R.A., Bogdanove, A.J., and Stoddard, B.L. (2012). The crystal structure of TAL effector PthXo1 bound to its DNA target. Science *335*, 716–719.

27. Yokota, M., Hatakeyama, H., Okabe, S., Ono, Y., and Goto, Y.i. (2015). Mitochondrial respiratory dysfunction caused by a heteroplasmic mitochondrial DNA mutation blocks cellular reprogramming. Hum. Mol. Genet. *24*, 4698–4709.

28. Venegas, V., and Halberg, M.C. (2012). Quantification of mtDNA mutation heteroplasmy (ARMS qPCR). Methods Mol. Biol. *837*, 313–326.

29. Gammage, P.A., Gaude, E., Van Haute, L., Rebelo-Guiomar, P., Jackson, C.B., Rorbach, J., Pekalski, M.L., Robinson, A.J., Charpentier, M., Concordet, J.P., et al. (2016). Near-complete elimination of mutant mtDNA by iterative or dynamic dose-controlled treatment with mtZFNs. Nucleic Acids Res. *44*, 7804–7816.

30. Tani, H., Ishikawa, K., Tamashiro, H., Ogasawara, E., Yasukawa, T., Matsuda, S., Shimizu, A., Kang, D., Hayashi, J.I., Wei, F.Y., et al. (2022). Aberrant RNA processing contributes to the pathogenesis of mitochondrial diseases in trans-mitochondrial mouse model carrying mitochondrial tRNALeu(UUR) with a pathogenic A2748G mutation. Nucleic Acids Res. *50*, 9382–9396.

31. Pang, C.Y., Lee, H.C., and Wei, Y.H. (2001). Enhanced oxidative damage in human cells harboring A3243G mutation of mitochondrial DNA: implication of oxidative stress in the pathogenesis of mitochondrial diabetes. Diabetes Res. Clin. Pract. *54*, S45–S56.

32. King, M.P., and Attardi, G. (1989). Human cells lacking mtDNA: repopulation with exogenous mitochondria by complementation. Science *246*, 500–503.

33. Cherry, A.B.C., Gagne, K.E., McLoughlin, E.M., Baccei, A., Gorman, B., Hartung, O., Miller, J.D., Zhang, J., Zon, R.L., Ince, T.A., et al. (2013). Induced pluripotent stem cells with a mitochondrial DNA deletion. Stem Cell. *31*, 1287–1297.

34. Chiaratti, M.R., and Chinnery, P.F. (2022). Modulating mitochondrial DNA mutations: factors shaping heteroplasmy in the germ line and somatic cells. Pharmacol. Res. *185*, 106466.

35. Moscou, M.J., and Bogdanove, A.J. (2009). A simple cipher governs DNA recognition by TAL effectors. Science *326*, 1501.

36. Boch, J., Scholze, H., Schornack, S., Landgraf, A., Hahn, S., Kay, S., Lahaye, T., Nickstadt, A., and Bonas, U. (2009). Breaking the code of DNA binding specificity of TAL-type III effectors. Science *326*, 1509–1512.

37. Cong, L., Zhou, R., Kuo, Y.C., Cunniff, M., and Zhang, F. (2012). Comprehensive interrogation of natural TALE DNA-binding modules and transcriptional repressor domains. Nat. Commun. *3*, 968.

38. Streubel, J., Blücher, C., Landgraf, A., and Boch, J. (2012). TAL effector RVD specificities and efficiencies. Nat. Biotechnol. *30*, 593–595.

39. Miller, J.C., Zhang, L., Xia, D.F., Campo, J.J., Ankoudinova, I.V., Guschin, D.Y., Babiarz, J.E., Meng, X., Hinkley, S.J., Lam, S.C., et al. (2015). Improved specificity of TALE-based genome editing using an expanded RVD repertoire. Nat. Methods *12*, 465–471.

40. Yang, J., Zhang, Y., Yuan, P., Zhou, Y., Cai, C., Ren, Q., Wen, D., Chu, C., Qi, H., and Wei, W. (2014). Complete decoding of TAL effectors for DNA recognition. Cell Res. *24*, 628–631.

41. Gautron, A.S., Juillerat, A., Guyot, V., Filhol, J.M., Dessez, E., Duclert, A., Duchateau, P., and Poirot, L. (2017). Fine and Predictable Tuning of TALEN Gene Editing Targeting for Improved T Cell Adoptive Immunotherapy. Mol. Ther. Nucleic Acids *9*, 312–321.

42. Moraes, C.T. (2024). Tools for editing the mammalian mitochondrial genome. Hum. Mol. Genet. *33*, R92–R99.

43. Yin, T., Luo, J., Huang, D., and Li, H. (2022). Current Progress of Mitochondrial Genome Editing by CRISPR. Front. Physiol. *13*, 883459.

44. Loutre, R., Heckel, A.M., Smirnova, A., Entelis, N., and Tarassov, I. (2018). Can Mitochondrial DNA be CRISPRized: Pro and Contra. IUBMB Life *70*, 1233–1239.

45. Li, Y., Wu, Y., Xu, R., Guo, J., Quan, F., Zhang, Y., Huang, D., Pei, Y., Gao, H., Liu, W., et al. (2023). In vivo imaging of mitochondrial DNA mutations using an integrated nano Cas12a sensor. Nat. Commun. *14*, 7722.

46. Sun, N., Bao, Z., Xiong, X., and Zhao, H. (2014). SunnyTALEN: a second-generation TALEN system for human genome editing. Biotechnol. Bioeng. *111*, 683–691.

47. Bacman, S.R., Kauppila, J.H.K., Pereira, C.V., Nissanka, N., Miranda, M., Pinto, M., Williams, S.L., Larsson, N.G., Stewart, J.B., and Moraes, C.T. (2018). MitoTALEN reduces mutant mtDNA load and restores tRNA(Ala) levels in a mouse model of heteroplasmic mtDNA mutation. Nat. Med. *24*, 1696–1700.

48. Gammage, P.A., Viscomi, C., Simard, M.L., Costa, A.S.H., Gaude, E., Powell, C.A., Van Haute, L., McCann, B.J., Rebelo-Guiomar, P., Cerutti, R., et al. (2018). Genome editing in mitochondria corrects a pathogenic mtDNA mutation in vivo. Nat. Med. *24*, 1691–1695.

49. Zekonyte, U., Bacman, S.R., Smith, J., Shoop, W., Pereira, C.V., Tomberlin, G., Stewart, J., Jantz, D., and Moraes, C.T. (2021). Mitochondrial targeted meganuclease as a platform to eliminate mutant mtDNA in vivo. Nat. Commun. *12*, 3210.

50. Bacman, S.R., Barrera-Paez, J.D., Pinto, M., Van Booven, D., Stewart, J.B., Griswold, A.J., and Moraes, C.T. (2024). mitoTALEN reduces the mutant mtDNA load in neurons. Mol. Ther. Nucleic Acids *35*, 102132.

51. Taha, E.A., Lee, J., and Hotta, A. (2022). Delivery of CRISPR-Cas tools for in vivo genome editing therapy: Trends and challenges. J. Contr. Release *342*, 345–361.

52. Winanto, Khong, Z.J., Khong, Z.J., Soh, B.S., Fan, Y., and Ng, S.Y. (2020). Organoid cultures of MELAS neural cells reveal hyperactive Notch signaling that impacts neurodevelopment. Cell Death Dis. *11*, 182.

53. Yokota, M., Hatakeyama, H., Ono, Y., Kanazawa, M., and Goto, Y.I. (2017). Mitochondrial respiratory dysfunction disturbs neuronal and cardiac lineage commitment of human iPSCs. Cell Death Dis. *8*, e2551.

54. Okita, K., Matsumura, Y., Sato, Y., Okada, A., Morizane, A., Okamoto, S., Hong, H., Nakagawa, M., Tanabe, K., Tezuka, K.i., et al. (2011). A more efficient method to generate integration-free human iPS cells. Nat. Methods *8*, 409–412.

55. Doyle, E.L., Booher, N.J., Standage, D.S., Voytas, D.F., Brendel, V.P., Vandyk, J.K., and Bogdanove, A.J. (2012). TAL Effector-Nucleotide Targeter (TALE-NT) 2.0: tools for TAL effector design and target prediction. Nucleic Acids Res. *40*, W117–W122.

56. Ochiai, H., Fujita, K., Suzuki, K.I., Nishikawa, M., Shibata, T., Sakamoto, N., and Yamamoto, T. (2010). Targeted mutagenesis in the sea urchin embryo using zinc-finger nucleases. Genes Cells *15*, 875–885.

57. Nakagawa, M., Taniguchi, Y., Senda, S., Takizawa, N., Ichisaka, T., Asano, K., Morizane, A., Doi, D., Takahashi, J., Nishizawa, M., et al. (2014). A novel efficient feeder-free culture system for the derivation of human induced pluripotent stem cells. Sci. Rep. *4*, 3594.

OMTN, Volume *36*

Supplemental information

Optimization of mtDNA-targeted platinum TALENs for bi-directionally modifying heteroplasmy levels in patient-derived m.3243A>G-iPSCs

Naoki Yahata, Yu-ichi Goto, and Ryuji Hata

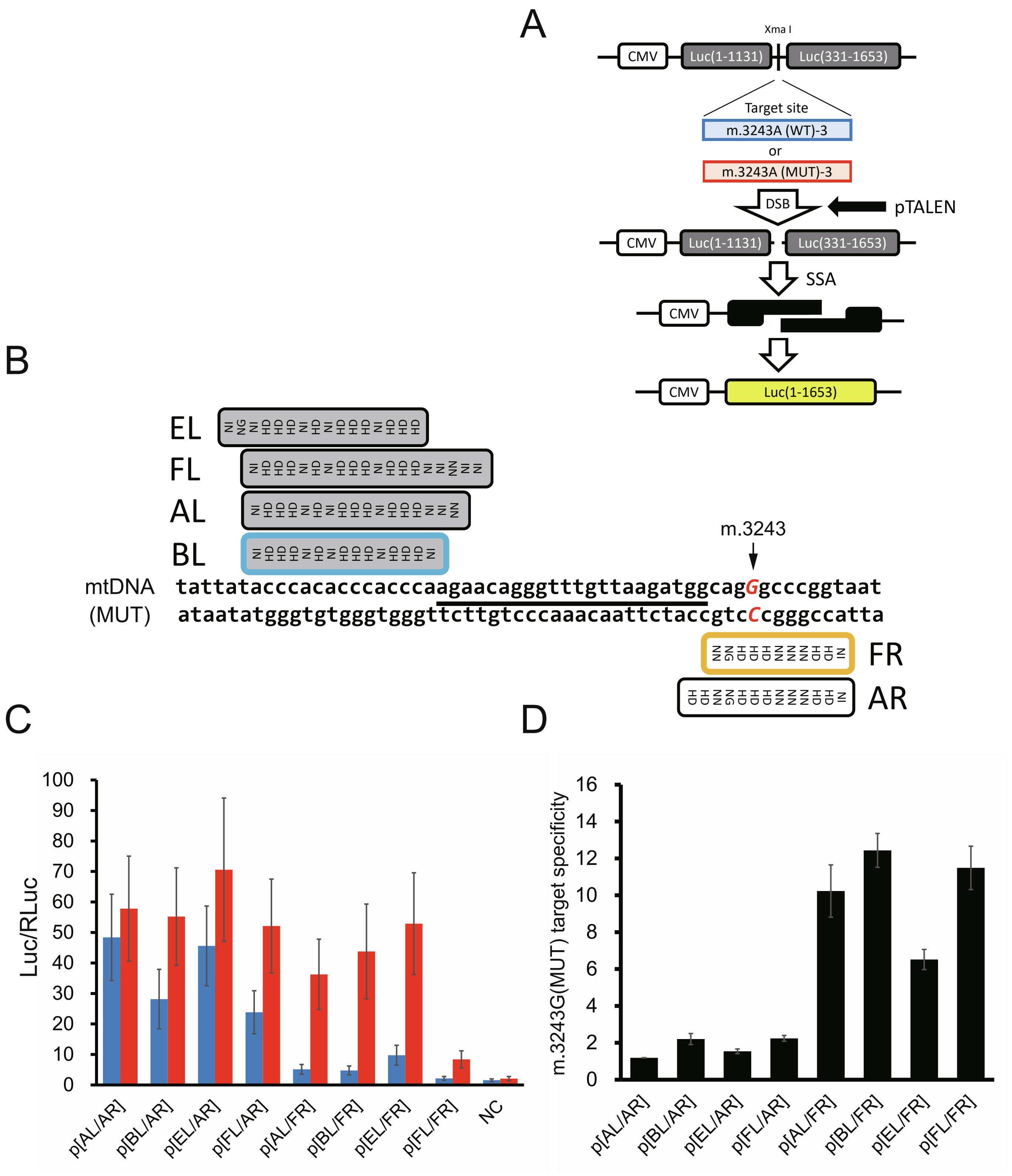

Figure S1

Functional evaluation of engineered m.3243G(MUT)-pTALENs using an SSA assay.

(A) Scheme of the SSA assay. The reporter plasmid encodes a part of the mtDNA sequence including m.3243A or m.3243G, named m.3243A(WT)-3 or m.3243G(MUT)-3, which is sandwiched by two split inactive parts of the luciferase gene with overlapping repeated sequences. Following a double-strand break caused by pTALENs, a functional luciferase gene is generated by an SSA reaction.

(B) Schematic design of m.3243G(MUT)-pTALENs. Gray and White boxes indicate left-pTALENs and right-pTALENs, respectively. Letters beside the boxes indicate the TALEs' names. The black bar indicates the spacer region bracketed by the pTALEN(BL)/pTALEN (FR) pair, abbreviated as p[BL/FR].

(C) Evaluation of the SSA activity (Luc/RLuc) of pTALEN pairs. Blue and red bars reflect the cleaving activities against m.3243A(WT)-3 and m.3243G(MUT)-3, respectively. NC, negative control. Data are expressed as mean ± SEM (n = 3).

(D) m.3243G(MUT) target specificity of each pTALEN pair. Data are expressed as means ± SEM (n = 3).

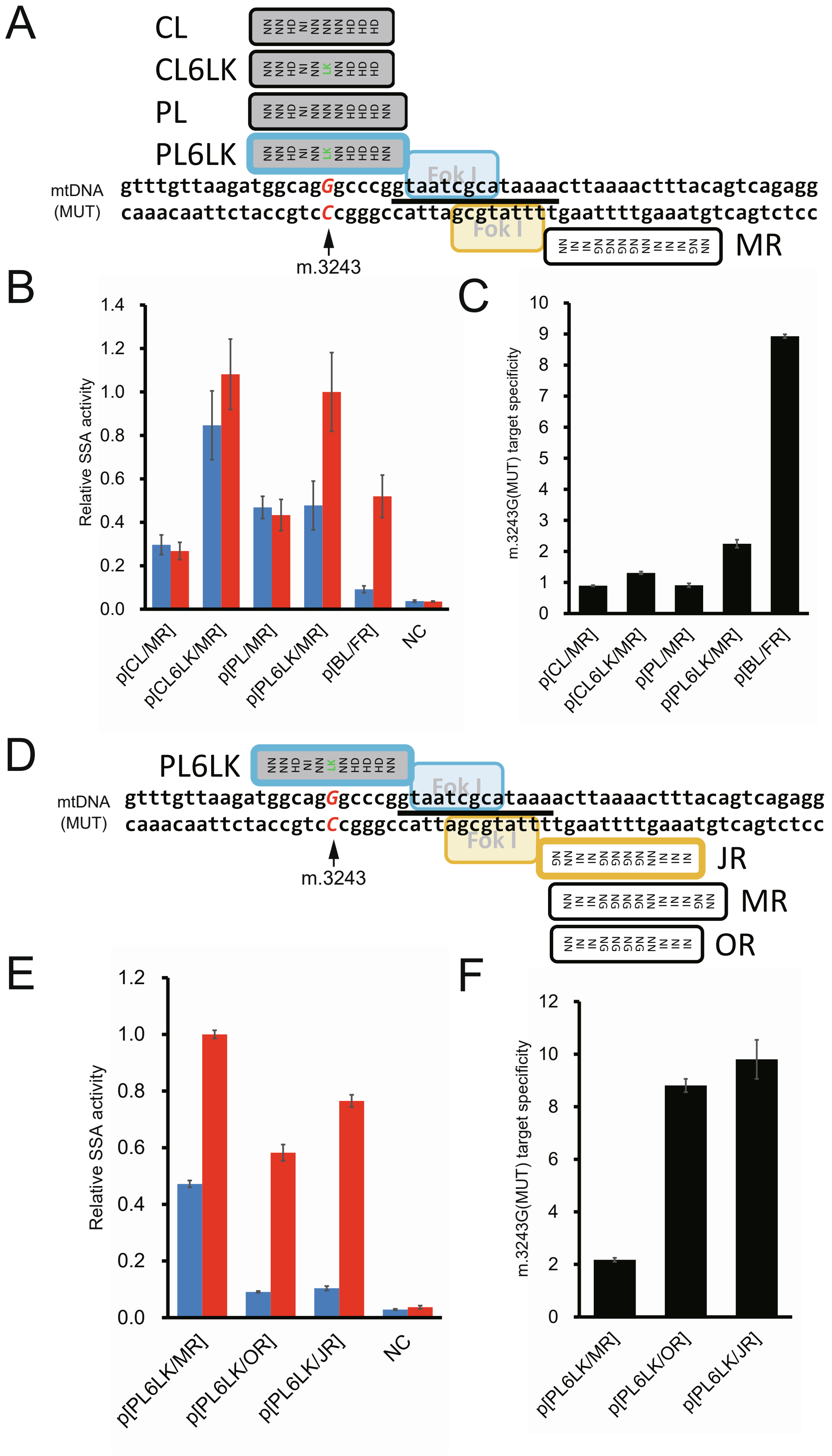

Figure S2

Functional evaluation of engineered m.3243G(MUT)-pTALENs with ncRVDs using an SSA assay.

(A, D) Schematic design of m.3243G(MUT)-pTALENs. Gray and white boxes indicate the left-pTALENs and right-pTALENs, respectively. Letters beside the boxes indicate the TALEs' names. The black bars indicate the spacer regions bracketed by the pTALEN (PL6LK)/pTALEN(MR) or pTALEN(PL6LK)/pTALEN(JR) pair, abbreviated as p[PL6LK/MR] (A) and p[PL6LK/JR] (D), respectively. Green letters indicate ncRVDs.

(B, E) Evaluation of the SSA activity (Luc/RLuc) of pTALEN pairs. Blue and red bars reflect the cleaving activities against human mtDNA sequences, including m.3243A(WT) and m.3243G(MUT), respectively. Relative SSA activity is defined as the ratio of the measured activity to the activity score of p[PL6LK/MR] against mutant mtDNA sequence. Data are expressed as the mean ± SEM (n = 3). NC, negative control.

(C, F) Specificity of each pTALEN pair toward the m.3243G(MUT). Data are expressed as the means ± SEM (n = 3).

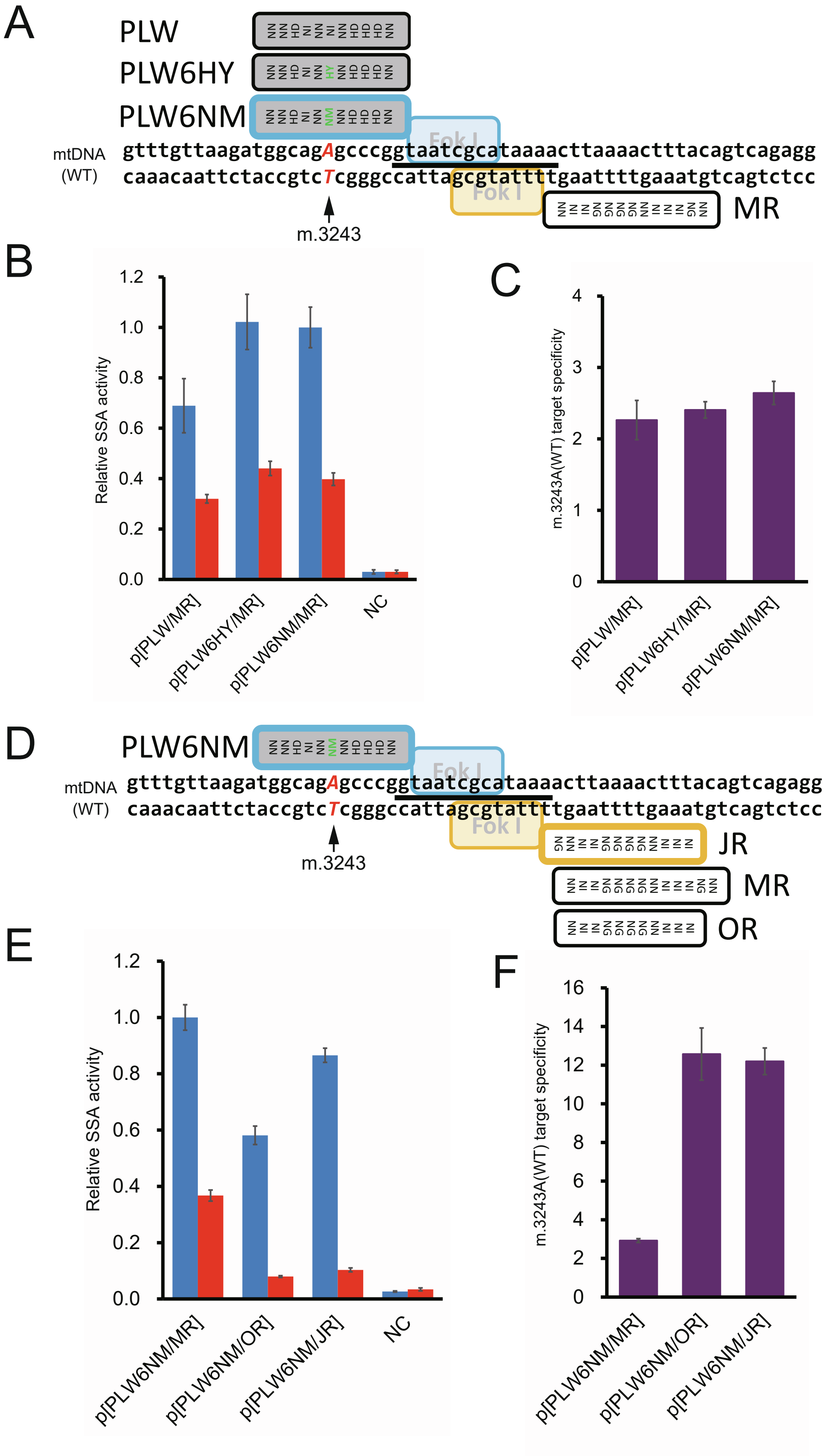

Figure S3

Functional evaluation of engineered m.3243A(WT)-pTALEN with ncRVDs using an SSA assay.

(A, D) Schematic design of m.3243A(WT)-pTALENs. Gray and white boxes indicate left-pTALENs and right-pTALENs, respectively. Letters beside the boxes indicate the TALEs' names. The black bars indicate the spacer regions bracketed by the pTALEN (PLW6NM)/pTALEN(MR) or pTALEN(PLW6NM)/pTALEN(JR) pair, abbreviated as p[PLW6NM/MR] (A) and p[PLW6NM/JR] (D), respectively. Green letters indicate ncRVDs.

(B, E) Evaluation of the SSA activity (Luc/RLuc) of pTALEN pairs. Blue and red bars reflect cleaving activity against human mtDNA sequences, including m.3243A(WT) and m.3243G(MUT), respectively. Relative SSA activity is defined as the ratio of the measured activity to the activity score of p[PLW6NM/MR] against wild-type mtDNA sequence. Data are expressed as the mean ± SEM (n = 3). NC, negative control.

(C, F) Specificity of each pTALEN pair toward the m.3243A(WT). Data are expressed as the means ± SEM (n = 3).

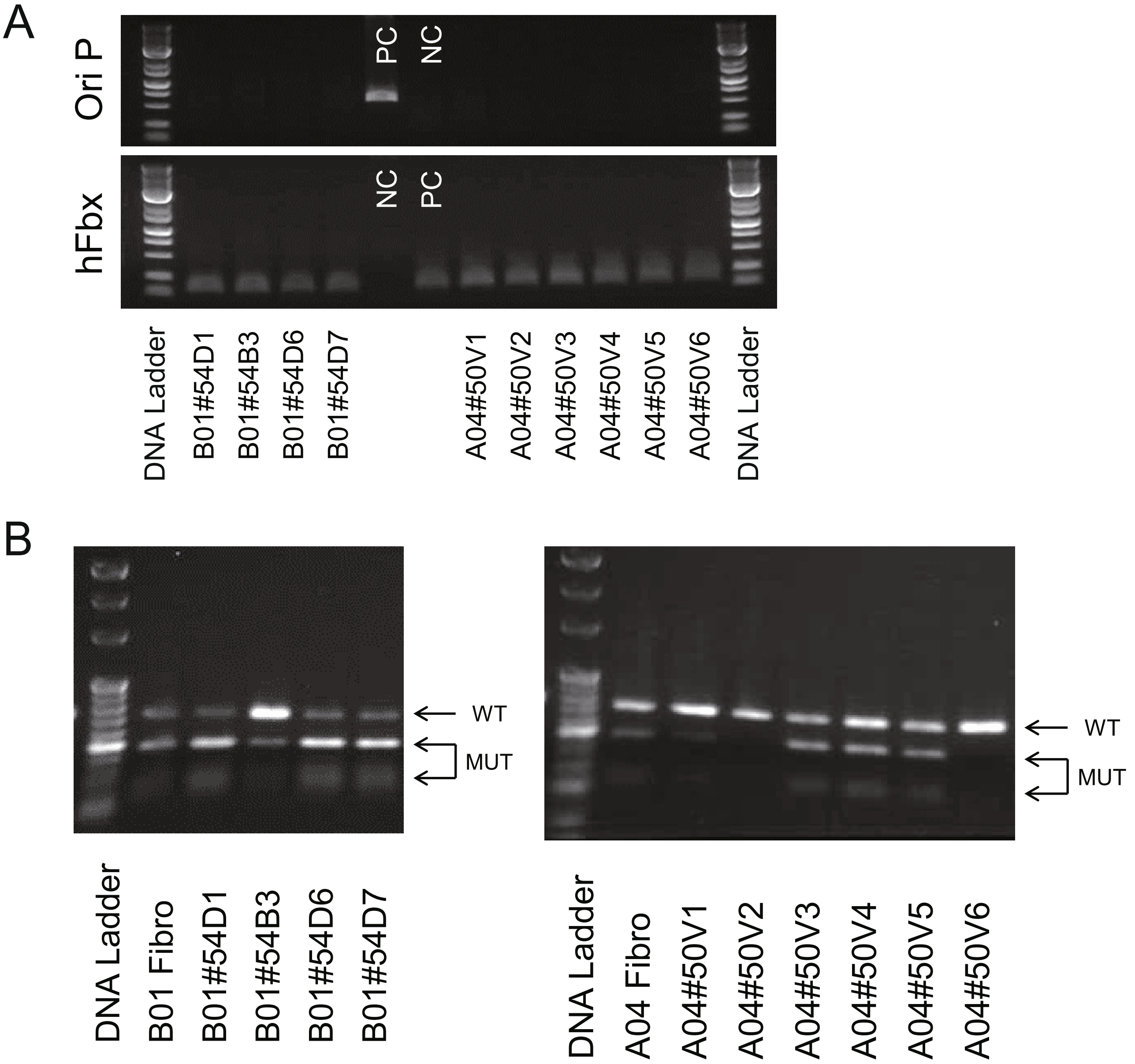

Figure S4

Validation of lack of transgene integration and m.3243A>G heteroplasmy level in isolated iPSC clones.

(A) PCR analysis for confirmation of transgene integration in isolated iPSC clones. In the top lane labeled OriP, PCR primer pair detected the sequence of OriP region in episomal vectors. The bottom lane labeled hFbx showed endogenous allele detection. Lane 6: reprogramming vector mix (pCXLE-hOCT3/4-shp53, pCXLE-hSK and pCXLE-hUL). Lane 7: genomic DNA of human skin fibroblasts. PC, positive control. NC, negative control.

(B) m.3243A>G heteroplasmy levels in mitochondrial disease patients' fibroblasts and isolated iPSC clones were analyzed by PCR-RFLP. The 145 bp and 95 bp + 50 bp fragments indicate the presence of wild-type and mutant mtDNA, respectively.

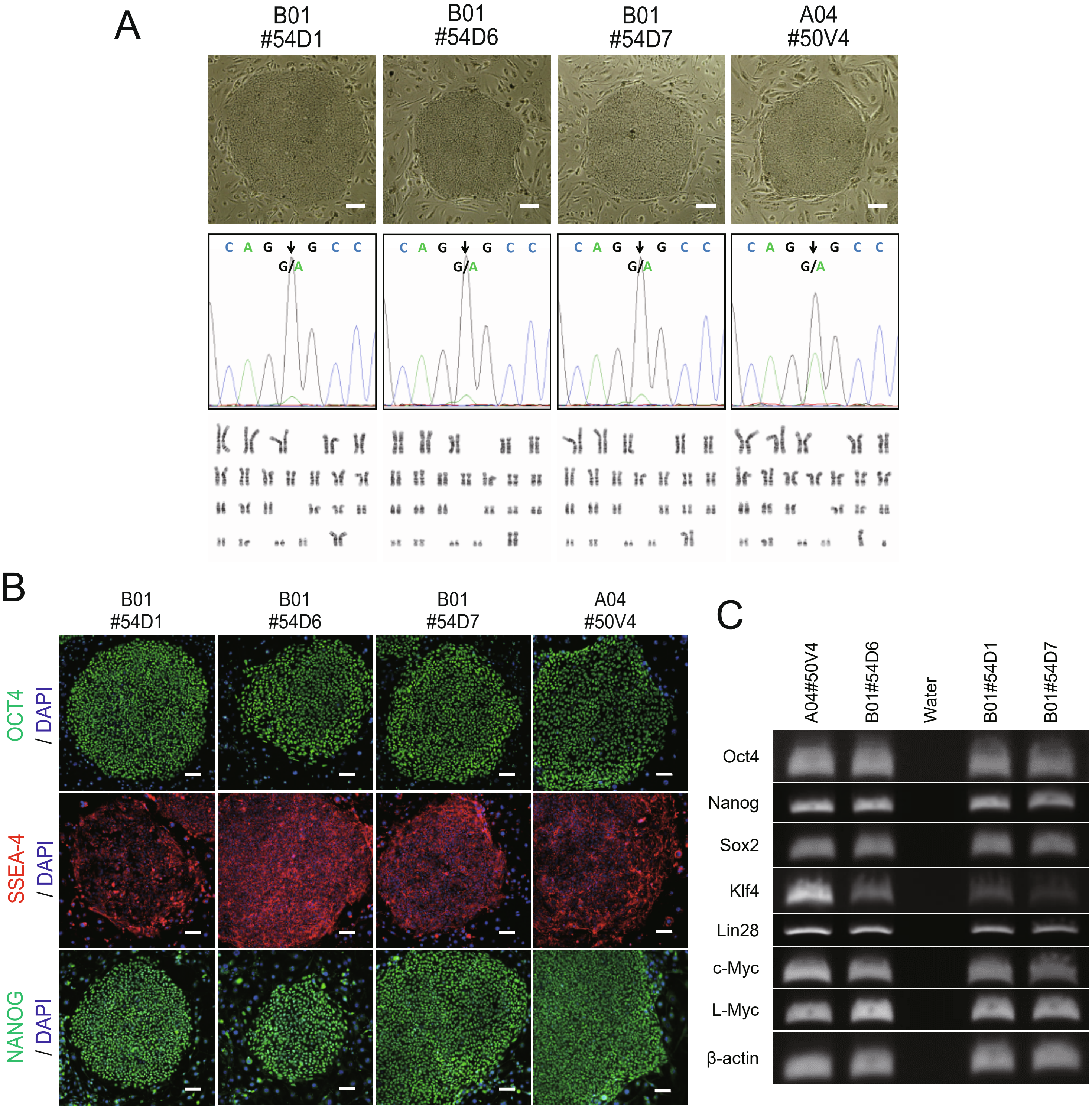

Figure S5

Establishment of human iPSCs from mitochondrial disease patients with m.3243A>G mutation.

(A) Top, phase-contrast images of established m.3243A>G-iPSC clones (B01_#54D1, #54D6, #54D7, and A04_#50V4). Scale bar, 200 μm. Middle, sequences of mtDNA extracted from the four iPSC clones. Arrows indicate the m.3243 position. Bottom, karyotype analysis revealed that these iPSC clones preserved a normal karyotype.

(B) These iPSC clones expressed the human ESC markers Oct4 (green), SSEA-4 (red), and Nanog (green) as indicated by immunocytochemical analysis. Scale bar, 200 μm.

(C) RT-PCR analyses of pluripotency markers and reprogramming factors (*Oct4*, *Nanog*, *Sox2*, *Klf4*, *Lin28*, *c-Myc*, and *L-Myc*). *β-actin* serves as a loading control.

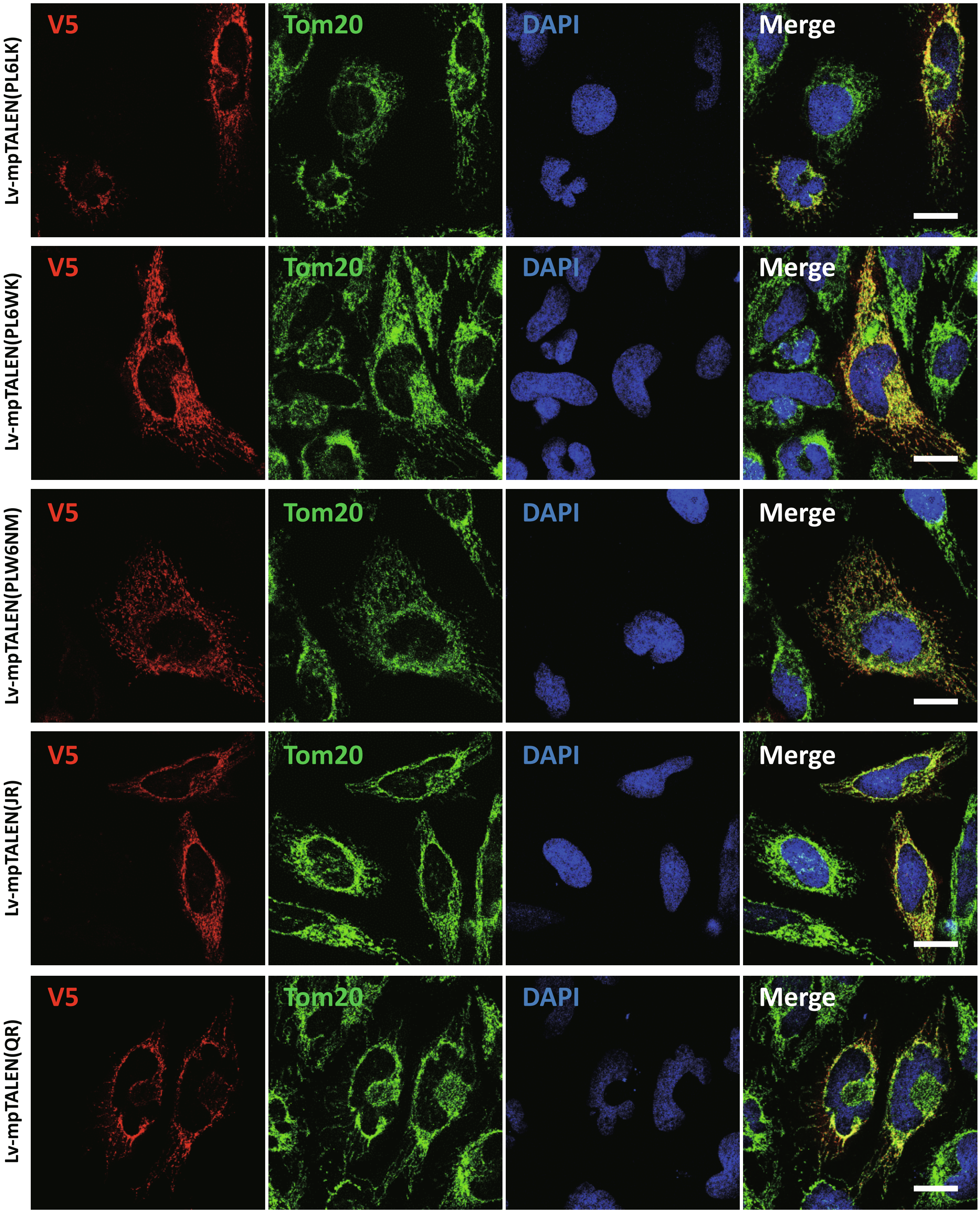

Figure S6

Mitochondrial localization of Lv-mpTALENs analyzed by immunocytochemistry.

The Lv-mpTALEN monomers were transiently expressed in HeLa cells. Two days after transfection, Lv-mp-TALEN(PL6LK), Lv-mpTALEN(PL6WK), Lv-mpTALEN(PLW6NM), Lv-mpTALEN(JR), and Lv-mpTALEN(QR) were stained using an anti-V5 antibody (red). Mitochondria were stained using an anti-TOM20 antibody (green). Nuclei were stained with DAPI (blue). Scale bar, 20 µm.

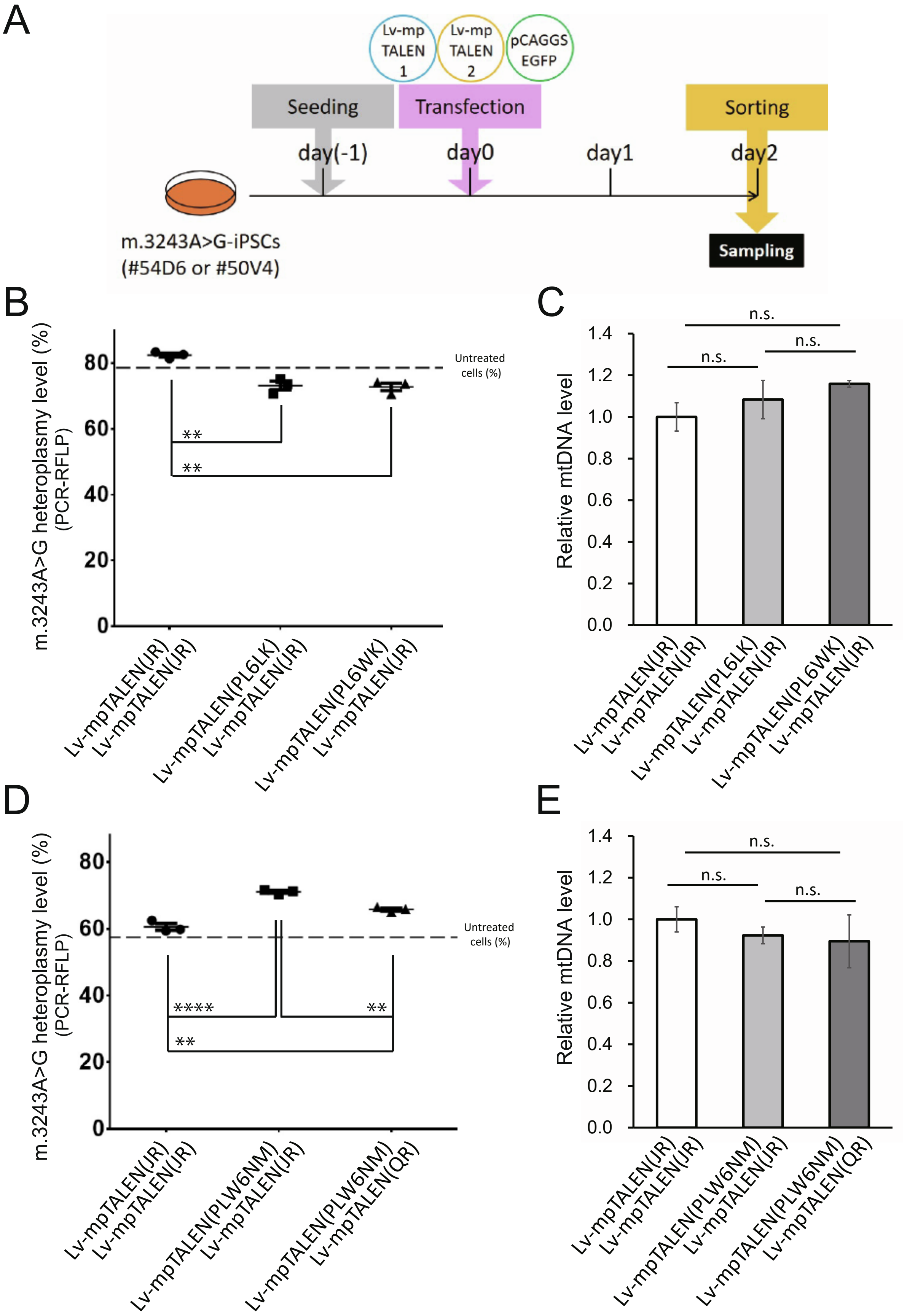

Figure S7

Effects of selected mpTALEN pairs on heteroplasmy levels in m.3243A>G-iPSCs.

(A) Experimental scheme for the application of mpTALEN pairs to m.3243A>G-iPSCs. m.3243A>G-iPSCs (#54D6 or #50V4) were transfected with plasmids coding left- and right-mpTALENs and EGFP. EGFP-positive and living cells were analyzed on day 2 after transfection.

(B, D) m.3243A>G heteroplasmy level in #54D6-iPSCs transfected with m.3243G(MUT)-mpTALEN pairs (B) and in #50V4-iPSCs transfected with m.3243A(WT)-mpTALEN pairs (D), respectively. Data are expressed as the means ± SEM (n = 3). Dotted lines indicate the heteroplasmy level in untreated cells on day 2. **$p < 0.01$, ****$p < 0.0001$ (one-way ANOVA, followed by Tukey' s multiple comparison test).

(C, E) Relative mtDNA levels (correlating with mtDNA copy number) in #54D6-iPSCs (C) and in #50V4-iPSCs (E), respectively. Data are presented as the ratio of the measured number to that in "mock-treated" controls (Lv-mpTALEN(JR)/Lv-mpTALEN(JR)) and expressed as the mean ± SEM (n = 3). n.s., not significant (one-way ANOVA, followed by Tukey' s multiple comparison test).

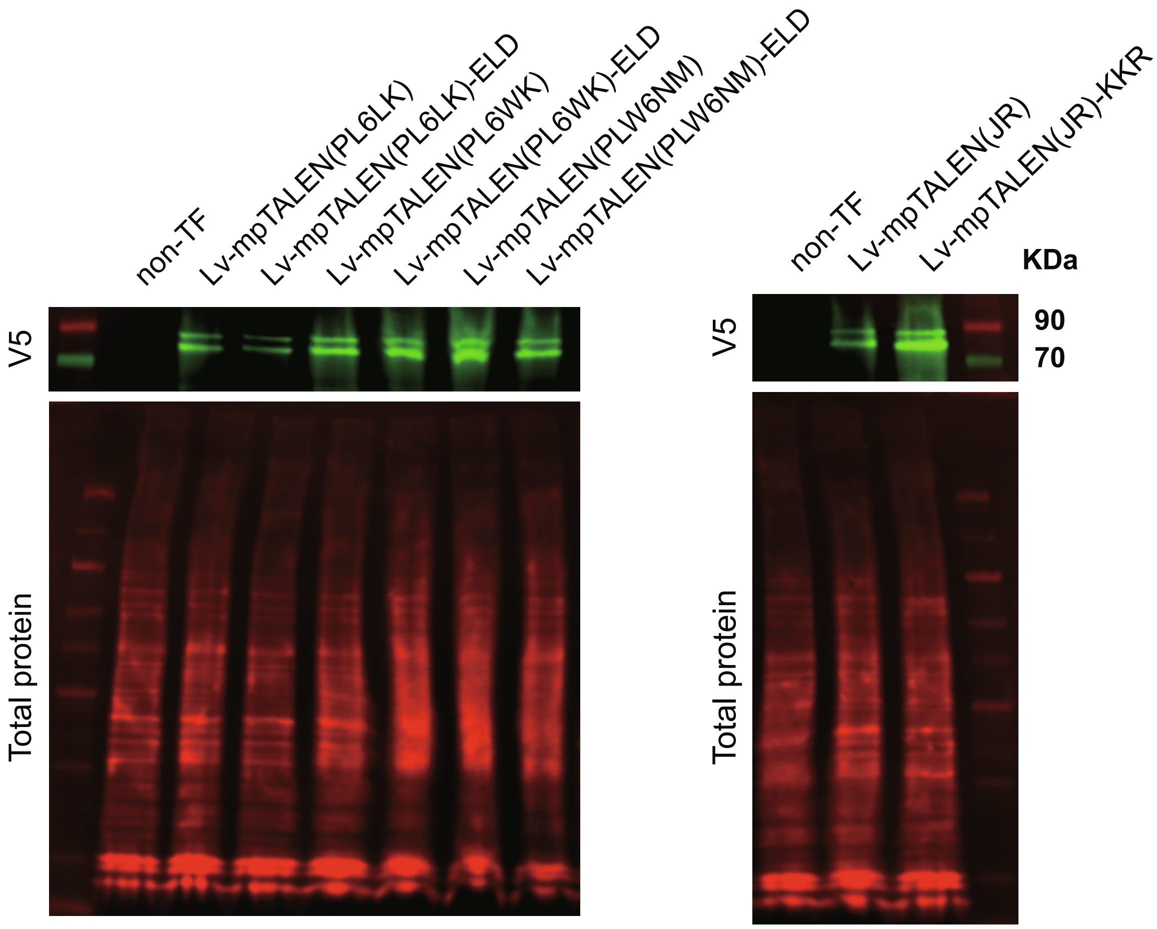

Figure S8

The mpTALEN proteins analyzed by Western blotting.

HEK293T cells were transfected with plasmids coding Lv-mpTALENs. Two days after transfection, the mpTALEN protein in cell extracts was detected by Western blotting using an anti-V5 antibody. Total protein was detected using Revert 700 Total Protein Stain (LI-COR). non-TF, non-transfected cells.

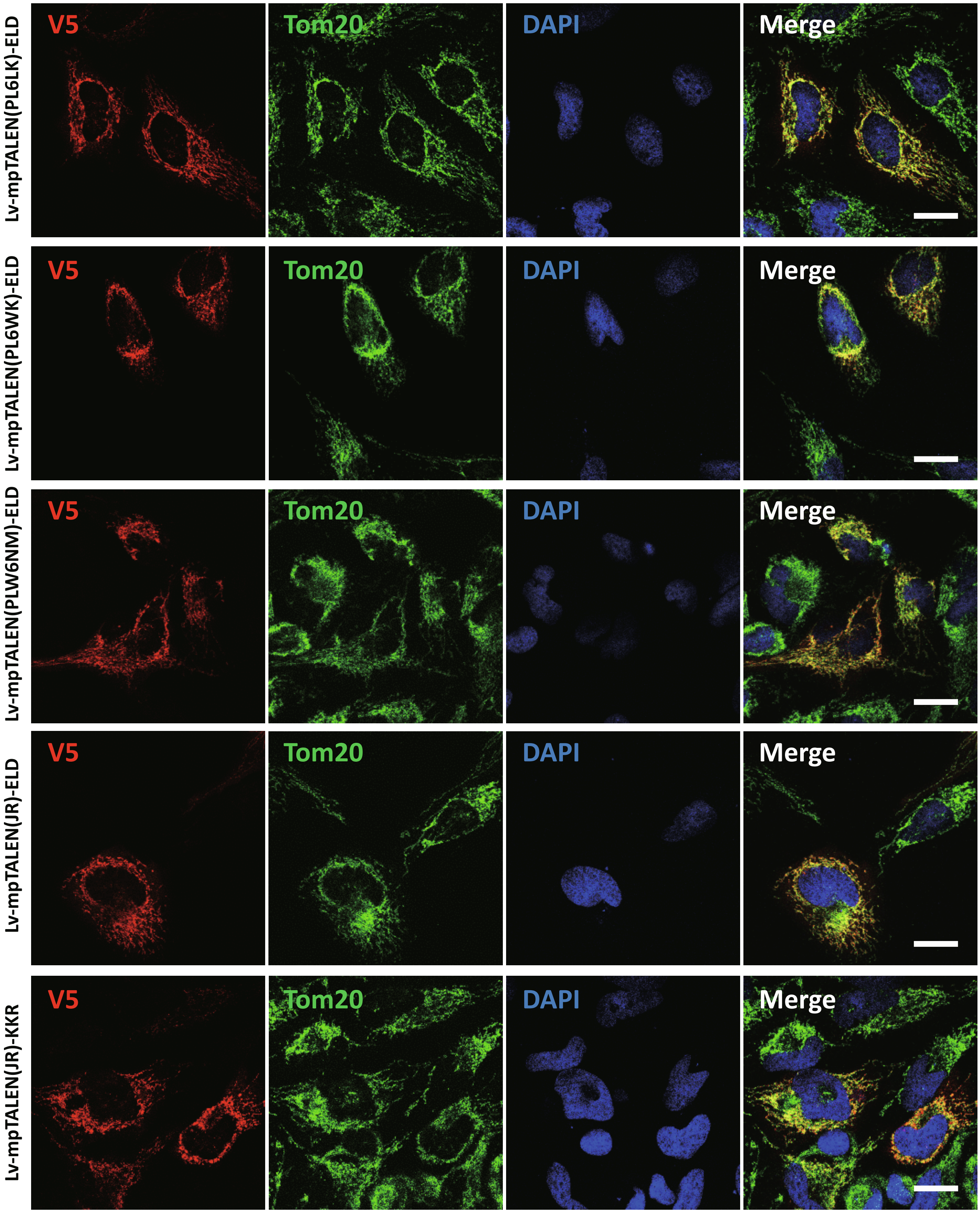

Figure S9

Mitochondrial localization of heterodimeric Lv-mpTALENs analyzed by immunocytochemistry.

The heterodimeric Lv-mpTALEN monomer was transiently expressed in HeLa cells. Two days after the transfection, Lv-mpTALEN (PL6LK)-ELD, Lv-mpTALEN(PL6WK)-ELD, Lv-mpTALEN(PLW6NM)-ELD, Lv-mpTALEN(JR)-ELD, and Lv-mpTALEN(JR)-KKR were stained with an anti-V5 antibody (red). Mitochondria were stained with an anti-TOM20 antibody (green). Nuclei were stained with DAPI (blue). Scale bar, 20 μm.

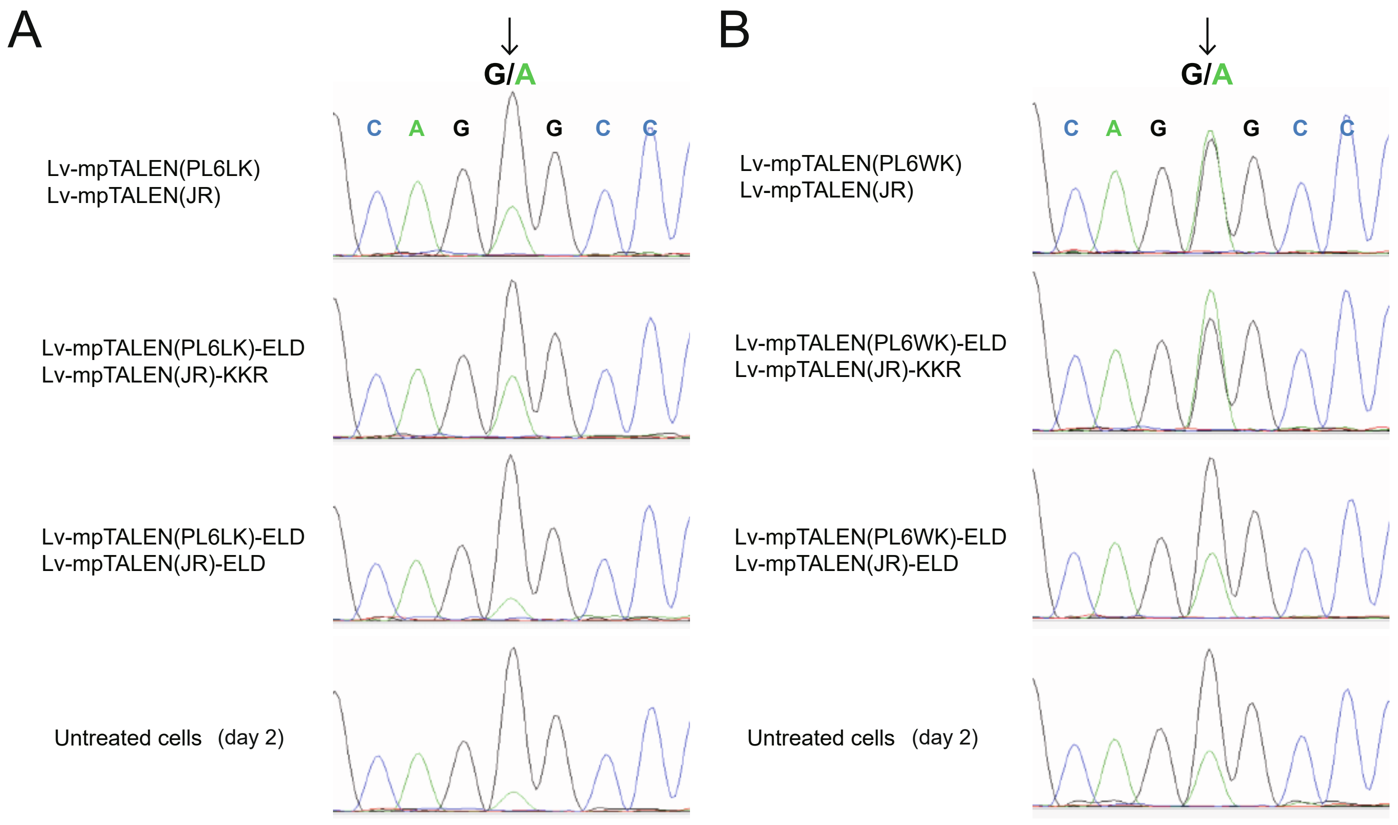

Figure S10

Sanger sequence analysis of m.3243A>G heteroplasmy in day 21 samples from Figures 3B and 3D data.

(A) Figure 3B and (B) FIgure 3D data. Arrow indicates the m.3243 position. The bottom data show electropherograms of untreated cells on day 2.

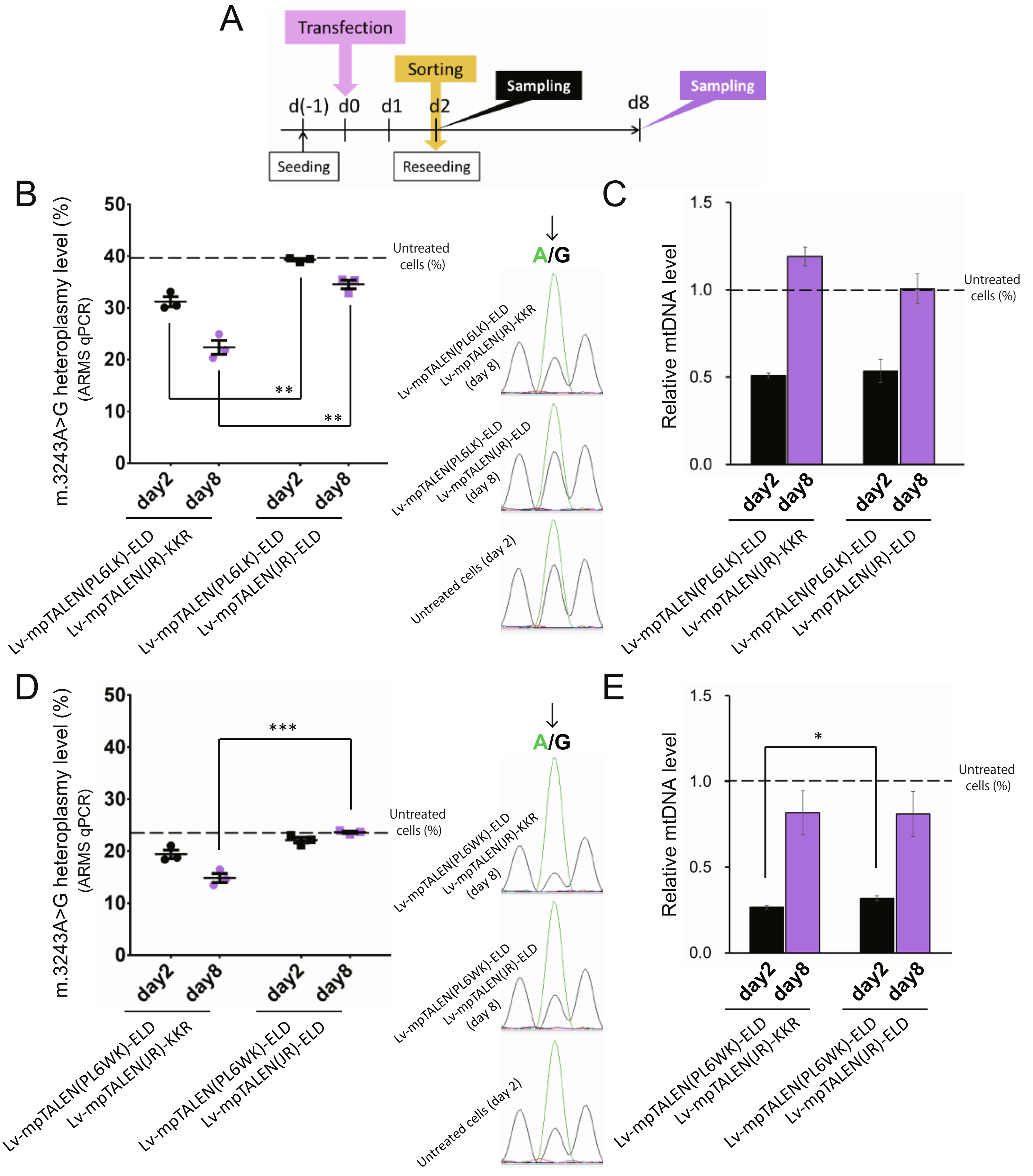

Figure S11

Effects of transient m.3243G(MUT)-mpTALEN expression on mtDNA heteroplasmy and copy numbers in #54D6-iPSCs.

(A) Experimental scheme. Two days after transfection, sorted cells were re-cultured without feeder cells for 6 days.

(B, D) Left: Heteroplasmy levels at 2 and 8 days after transfection. Dotted line indicates the heteroplasmy level in untreated cells at day 2. Data are expressed as the means ± SEM (n = 3). $^{**}p < 0.01$, $^{***}p < 0.001$ (Holm-Sidak test). Right: Sanger sequencing data. Arrow indicates the m.3243 position.

(C, E) Relative mtDNA levels (correlating with mtDNA copy numbers) at 2 and 10 days after transfection. Data are presented as the ratio of the measured number to that in untreated cells at day 2 and expressed as the means ± SEM (n = 3). The dotted line indicates the relative copy number in untreated cells on day 2. $^{*}p < 0.05$ (Holm-Sidak test).

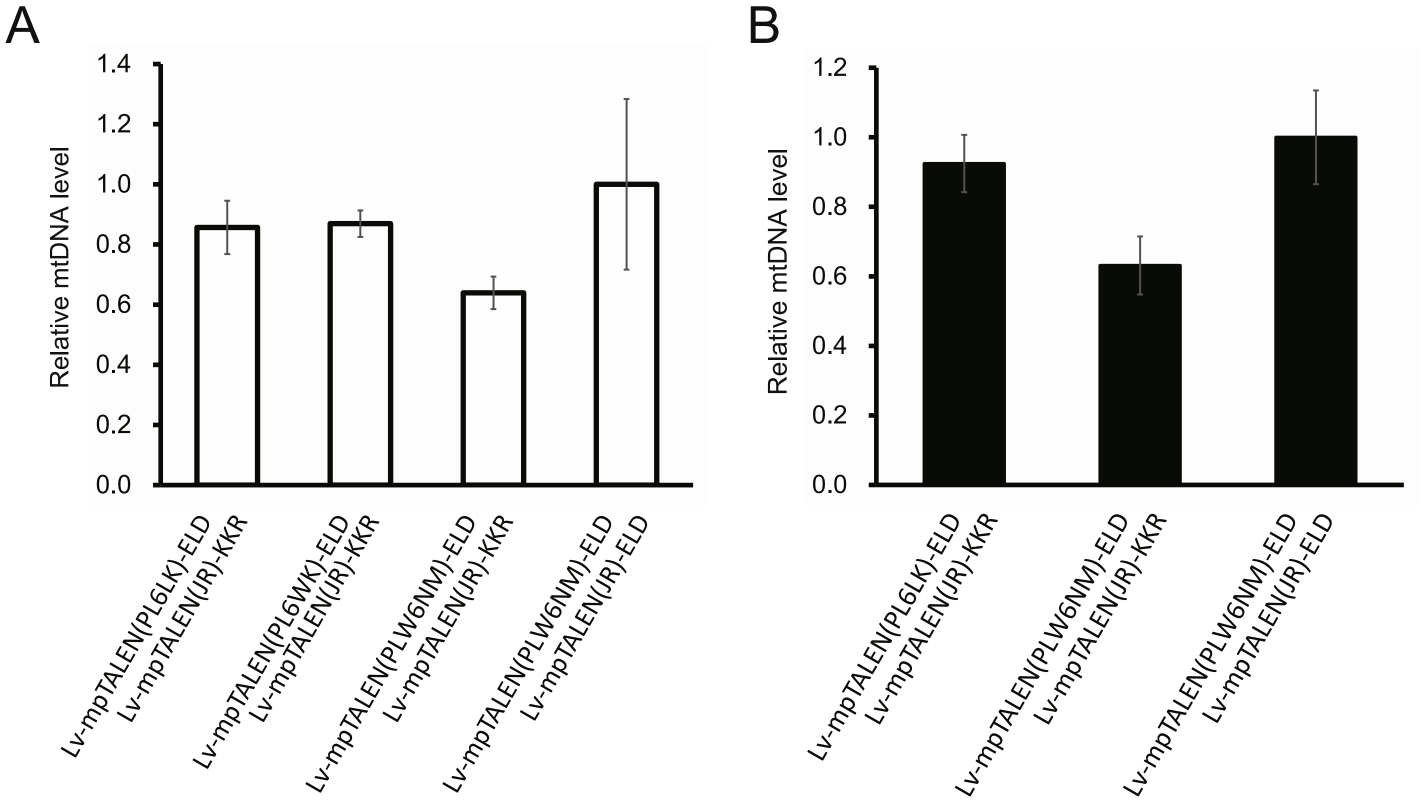

Figure S12

Effects of transient mpTALEN expression on mtDNA copy numbers in m.3243A(WT) homoplasmic cells.

(A) Relative mtDNA levels (correlating with mtDNA copy numbers) in sorted HEK293T cells at 2 day after transfection.

Data are presented relative to those of Lv-mpTALEN(PLW6NM)-ELD/Lv-mpTALEN(JR)-ELD and expressed as the mean ± SEM (n = 3).

There were no significant differences ($p > 0.05$, one-way ANOVA, followed by Tukey' s multiple comparison test).

(B) Relative mtDNA levels (correlating with mtDNA copy numbers) in sorted A01#15_MyoD33-4 iPSCs at 2 day after transfection.

Data are presented relative to those of Lv-mpTALEN(PLW6NM)-ELD/Lv-mpTALEN(JR)-ELD and expressed as the means ± SEM (n = 3).

There were no significant differences ($p > 0.05$, one-way ANOVA, followed by Tukey' s multiple comparison test).

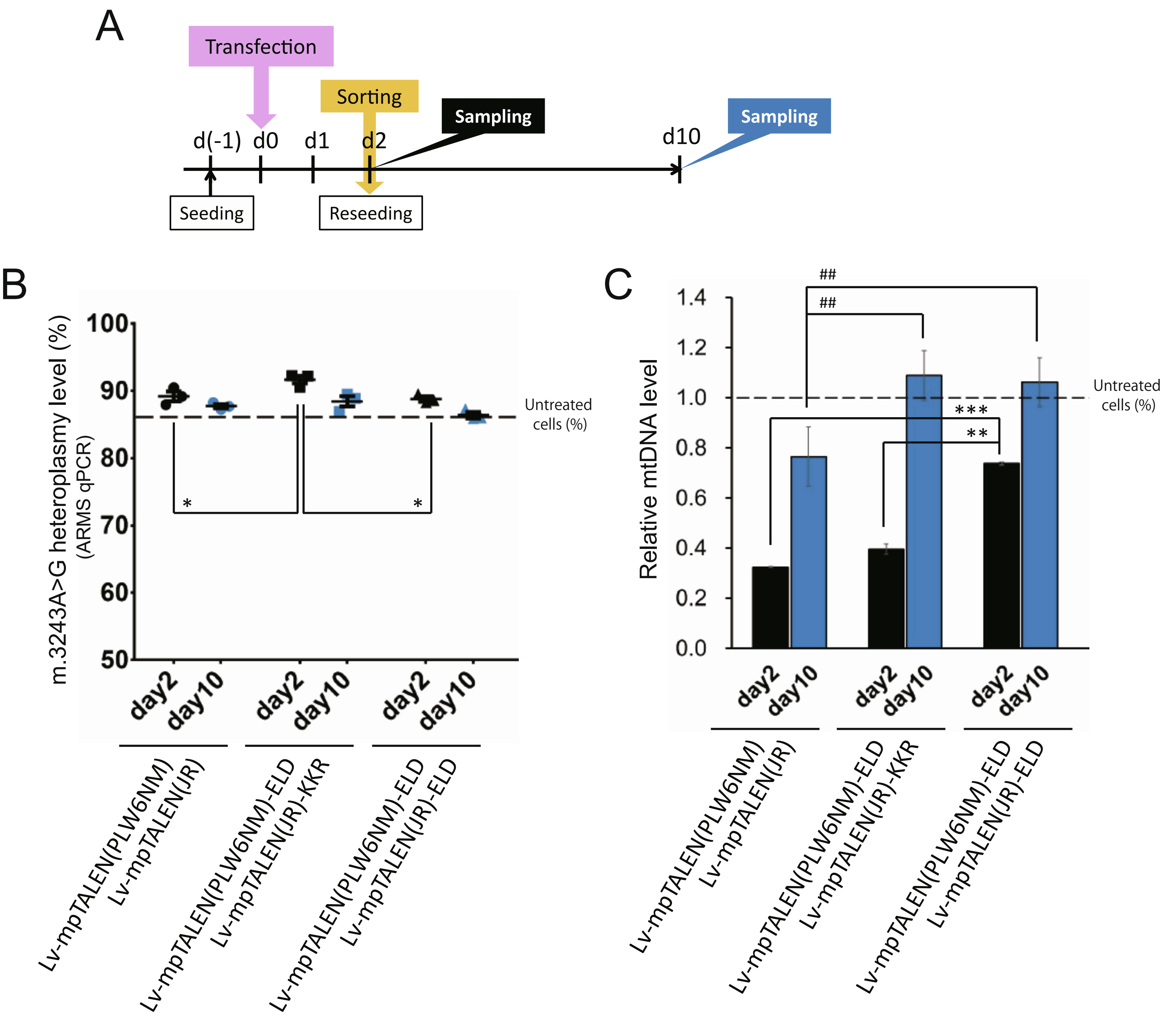

Figure S13

Effects of transient m.3243A(WT)-mpTALEN expression on mtDNA heteroplasmy and copy numbers in #54D6-iPSCs.

(A) Experimental scheme. Two days after transfection, sorted cells were re-cultured without feeder cells for 8 days.

(B) Heteroplasmy levels at 2 and 10 days after transfection. Dotted line indicates the heteroplasmy level in untreated cells at day 2. Data are expressed as the means ± SEM (n = 3). *p < 0.05 (two-way ANOVA, followed by Tukey' s multiple comparison test).

(C) Relative mtDNA levels (correlating with mtDNA copy numbers) at 2 and 10 days after transfection. Data are presented as the ratio of the measured number to that in untreated cells at day 2 and expressed as the means ± SEM (n = 3). The dotted line indicates the relative copy number in untreated cells on day 2. $^{**, \#\#}$ p < 0.01, ***p < 0.001 (two-way ANOVA, followed by Tukey' s multiple comparison test).

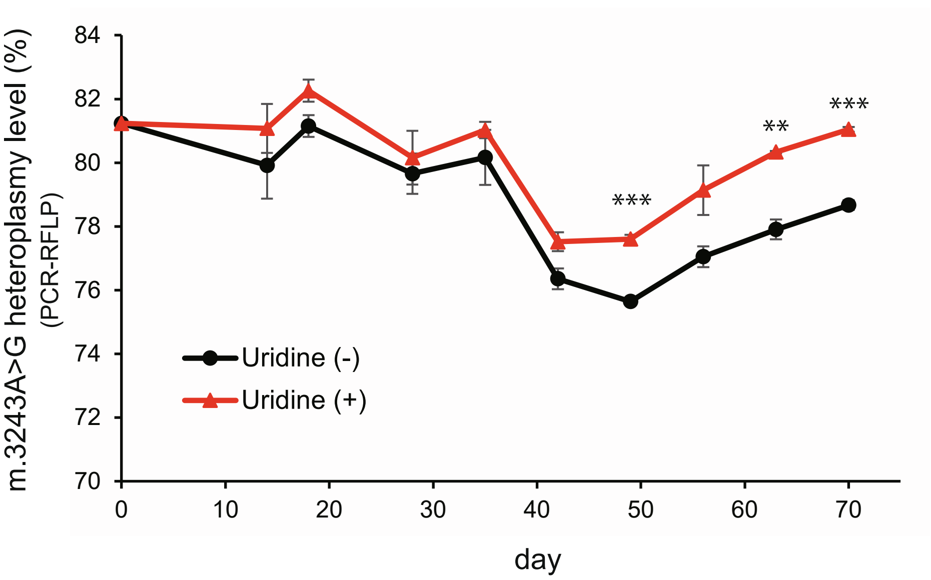

Figure S14

Effect of uridine supplementation on fluctuations of the m.3243A>G heteroplasmy levels in #54D6-iPSCs.

Long-term cultivation of #54D6-iPSCs was performed in feeder-free condition in the absence (black) or presence (red) of 50 µg/ml uridine. The m.3243A>G heteroplasmy levels were analyzed by PCR-RFLP at each passage point. Data are expressed as the means ± SEM of triplicate wells. Statistical significance was determined using the Holm-Sidak method for multiple comparisons. **$p < 0.01$, ***$p < 0.001$.

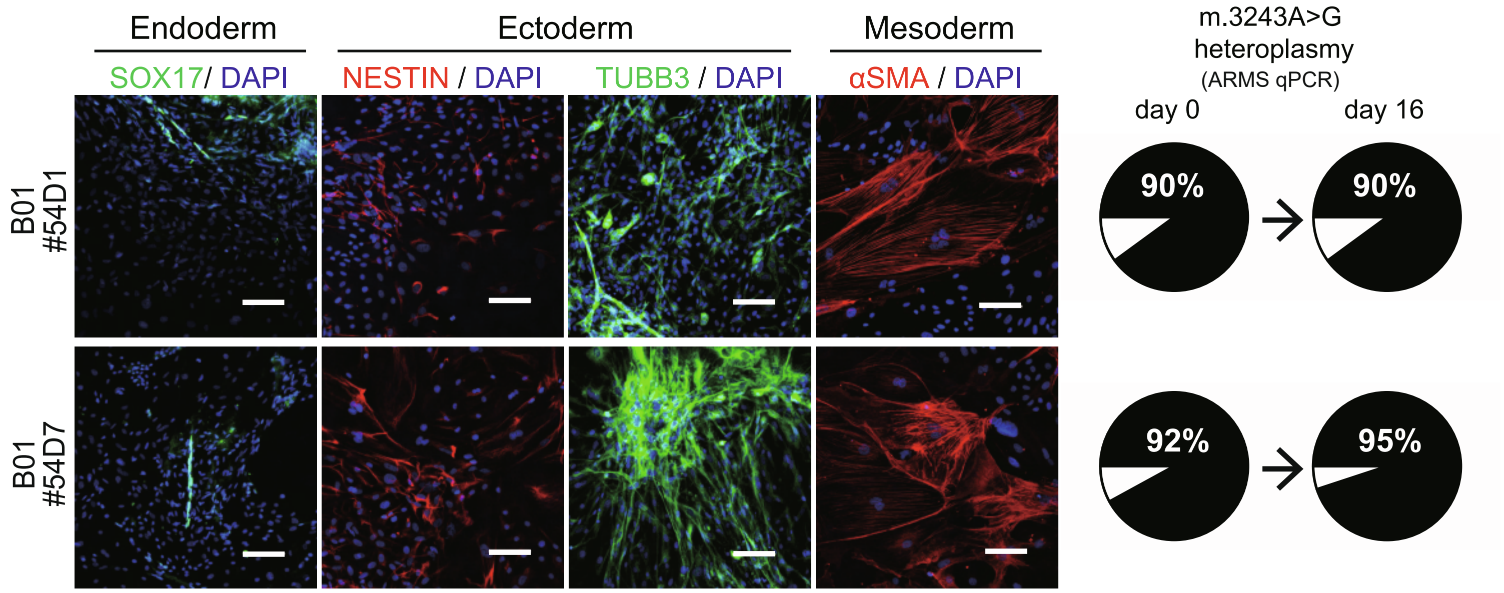

Figure S15

EB-mediated differentiation of #54D1- and #54D7-iPSCs with m.3243A>G mutation.

In vitro differentiation of two m.3243A>G-iPSC clones (B01_#54D1 and #54D7) into all three germ layers. Cells were labeled with anti-Sox17 (endoderm, green), anti-Nestin (ectoderm, red), anti-TUBB3 (ectoderm, green), and anti-αSMA (mesoderm, red) antibodies. Nuclei were stained with DAPI (blue). Scale bar, 100 µm. Pie charts at the right side show m.3243A>G heteroplasmy in undifferentiated iPSCs on day 0 and in differentiated cells on day 16, calculated by ARMS-qPCR.

Table S1: Primer list

Name	Sequence	Name	Sequence
Mito-1-2F	CATAGCACATTACAGTCAAATCCCTTCTCGTCCC	Mito-1-2R	ATTGCTAGGGTGGCGCTTCCAATTAGGTGC
mt3150-F	TACTTCACAAAGCGCCTTCC	mt3294-R	AGGAATTGAACCTCTGACTG
ACTB-F	CCAACCGCGAGAAGATGA	ACTB-R	TCCATCACGATGCCAGTG
rt-Oct3/4-F	GACAGGGGGAGGGGAGGAGCTAGG	rt-Oct3/4-R	CTTCCCTCCAACCAGTTGCCCCAAAC
rt-Nanog-F	CAGCCCCGATTCTTCCACCAGTCCC	rt-Nanog-R	CGGAAGATTCCCAGTCGGGTTCACC
rt-Sox2-F	GGGAAATGGGAGGGGTGCAAAAGAGG	rt-Sox2-R	TTGCGTGAGTGTGGATGGGATTGGTG
rt-Klf4-F	ACGATCGTGGCCCCGGAAAAGGACC	rt-Klf4-R	TGATTGTAGTGCTTTCTGGCTGGGCTCC
rt-L-Myc-F	GTGAGTCCCCCACCTGTAGA	rt-L-Myc-R	TTAGTAGCCAGTGAGGTATGCAATTC
rt c-Myc-F	GCGTCCTGGGAAGGGAGATCCGGAGC	rt c-Myc-R	TTGAGGGGCATCGTCGCGGGAGGCTG
rt-Lin28-F	CACCATGGGCTCCGTGTCCAACCAGCAG	rt-Lin28-R	TCAATTCTGTGCCTCCGGGAGCAGGGTAGG
MT-CYTB-F	TGCAACTATAGCAACAGCCTTCA	MT-CYTB-R	GAACTAGGTCTGTCCCAATGTATGG
FBXO15-F	GCCAGGAGGTCTTCGCTGTA	FBXO15-R	AATGCACGGCTAGGGTCAAA
pEP4-SF1	TTCCACGAGGGTAGTGAACC	pEP4-SR1	TCGGGGGTGTTAGAGACAAC
hFbx15-2F	GCCAGGAGGTCTTCGCTGTA	hFbx15-2R	AATGCACGGCTAGGGTCAAA
XmaI-A3243_F	TTAcccgggTATTATACCCACACCCACCC	XmaI-A3243AWT_R3	TTACCCGGGtgaacctctgactgtaaagttttaagttttatgcgattaccgggcT
		XmaI-A3243G_R3	TTACCCGGGtgaacctctgactgtaaagttttaagttttatgcgattaccgggcC
BamHI_Fok1_fwd	ACCAACAGAAGGATCCCCGA	K1-Fok1_rev	ATTTTCcttGACATAACGTTGCATCTC
K1-Fok2_fwd	TATGTCaagGAAAATCAGACCAGGAAC	R2K3-Fok2_rev	TTCGTctttctATTCAACCGTGTGAGCTG
R2K3-Fok3_fwd	GAATagaaagACGAACTGCAATGGTGCG	SexAI_Fok3_rev	ACTTTCCACACCTGGTTGCT
ARMS-A3243G(WT)_F1	CAGGGTTTGTTAAGATGGCAtA	ARMS-A3243G_R1	TGGCCATGGGTATGTTGTTA
ARMS-A3243G(MUT)_F1	CAGGGTTTGTTAAGATGGCAtG		
